# STT3A-mediated FCN3 N-glycosylation promotes Treg cell activation to drive hepatocellular carcinoma progression via Wnt/β-catenin

**DOI:** 10.1007/s13402-025-01159-1

**Published:** 2026-01-06

**Authors:** Hongli Zhang, Peng Zhang, Tian Gong, Chengsheng Zhang, Zhijian Wang, Yong Zhao

**Affiliations:** 1 Department of Medical Genetics, The First Affiliated Hospital of Nanchang University, Jiangxi Medical College, Nanchang, Jiangxi 330006 China; 2 Center for Molecular Diagnosis and Precision Medicine, The First Affiliated Hospital of Nanchang University, Jiangxi Medical College, Nanchang, Jiangxi 330006 China; 3 Department of Pain Medicine, The First Affiliated Hospital of Nanchang University, Jiangxi Medical College, Nanchang, Jiangxi 330006 China; 4 Jiangxi Key Laboratory of Trauma, Burn and Pain Medicine, The First Affiliated Hospital of Nanchang University, Jiangxi Medical College, Nanchang, Jiangxi 330006 China; 5 Key Laboratory of Neuropathic Pain, Healthcare Commission of Jiangxi Province, The First Affiliated Hospital of Nanchang University, Jiangxi Medical College, Nanchang, Jiangxi 330006 China

**Keywords:** FCN3, N-glycosylation, STT3A, Regulatory T cells, Wnt/β-catenin signaling

## Abstract

**Background:**

Hepatocellular carcinoma (HCC) remains a leading cause of cancer mortality worldwide. While regulatory T (Treg) cells are known to contribute to HCC progression, the molecular mechanisms regulating their activation, particularly those involving post-translational modifications, remain poorly understood.

**Methods:**

This study employed a comprehensive approach combining clinical sample analysis, in vitro cell models (HepG2, Hep3B, HCC-LM3) and in vivo xenograft experiments. Genetic manipulation involved lentiviral-mediated knockdown or overexpression of FCN3 and STT3A. Techniques included immunofluorescence, co-immunoprecipitation, glycosylation validation, CCK-8, wound healing, Transwell and flow cytometry.

**Results:**

Clinical data revealed significant downregulation of FCN3 in HCC tissues, correlating with poor patient survival. Mechanistically, FCN3 suppressed Treg activation and HCC progression by inhibiting Wnt/β-catenin signaling through APC upregulation. However, the glycosyltransferase STT3A mediated N-glycosylation of FCN3 at Asn189, thereby disrupting the tumor-suppressive function of FCN3 in HCC. In mouse models, STT3A knockdown reduced tumor growth and decreased Treg infiltration. Additionally, the Treg cell-depleting agent diphtheria toxin could reverse the promoting effect of STT3A overexpression on HCC tumor growth.

**Conclusion:**

This research unveiled a novel STT3A-FCN3-β-catenin axis that drove HCC progression through glycosylation-dependent Treg activation. These findings provided new insights into immune evasion mechanisms and highlighted potential therapeutic opportunities for HCC.

**Supplementary Information:**

The online version contains supplementary material available at 10.1007/s13402-025-01159-1.

## Introduction

Hepatocellular carcinoma (HCC) is the leading cause of cancer-related mortality worldwide, yet current treatment options for advanced-stage patients remain suboptimal [1, 2]. Although significant progress has been made in immunotherapy in recent years, the immunosuppressive tumor microenvironment (TME) substantially compromises therapeutic efficacy in HCC [3, 4]. Regulatory T cells (Tregs), as crucial immunosuppressive components within the TME [5, 6], have been demonstrated to play a pivotal role in HCC progression [7, 8]. However, the molecular mechanisms governing Treg cell activation in HCC are still not fully understood.

The Wnt/β-catenin signaling pathway is frequently dysregulated in HCC and plays essential roles in multiple biological processes including proliferation, invasion, migration and TME modulation [9, 10]. In colorectal cancer, inhibition of Wnt/β-catenin signaling has been shown to suppress tumor growth by reducing Treg cell infiltration and sensitize cancer cells to PD-1 inhibitors [11]. Similarly, Lu et al. have reported that β-catenin blockade inhibits gastric cancer progression by suppressing Treg cell recruitment [12]. Ficolin-3 (FCN3), a member of the ficolin family and a component of the innate immune system, has been implicated in HCC development [13]. Previous studies have demonstrated that FCN3 suppresses HCC progression through multiple mechanisms including complement activation [14], induction of ribosomal stress [15] and promotion of ferroptosis [16]. Notably, our bioinformatics analysis predicts potential interactions between FCN3 and APC, a key component of the Wnt signaling pathway. Nevertheless, whether FCN3 regulates Treg cell activity through Wnt signaling remains unexplored.

N-glycosylation, the most common protein modification in the eukaryotic secretory pathway, significantly influences protein binding capacity and stability [17]. Aberrant N-glycosylation can disrupt cellular metabolism and signaling pathways, thereby promoting cancer progression [18, 19]. Compared with adjacent non-tumor tissues, HCC exhibits significantly dysregulated N-glycosylation patterns [20]. According to the UniProt database, FCN3 contains a conserved N-glycosylation site at asparagine 189 (Asn189) [21], while BioGRID interaction analysis further reveals that FCN3 interacts with multiple glycosylation-related proteins. The oligosaccharyltransferase (OST) complex, essential for N-linked protein glycosylation, catalyzes the transfer of preassembled oligosaccharides to asparagine residues [22]. The OST complex catalytic subunit A (STT3A) mediates N-glycosylation of numerous proteins [22, 23] and has been strongly associated with tumor progression [24–26]. Intriguingly, STT3A expression has been reported to correlate with Treg cell infiltration in HCC [27], although the underlying mechanisms remain elusive.

This study systematically investigates the role of FCN3 in HCC progression and Treg cell regulation, with a focus on STT3A-mediated N-glycosylation modifications. Our findings reveal a novel STT3A-FCN3-β-catenin signaling axis that promotes HCC progression by modulating Treg-mediated immunosuppression, thereby providing crucial insights into HCC immune evasion mechanisms and potential therapeutic targets.

## Materials and methods

### Clinical samples

Paired tumor and adjacent non-tumor liver tissues were collected from 4 HCC patients undergoing surgical resection at The First Affiliated Hospital of Nanchang University, Jiangxi Medical College, with written informed consent. Patients with preoperative treatments or other malignancies were excluded. All procedures were conducted in accordance with the ethical principles outlined in the Declaration of Helsinki.

### Animals

Male C57BL/6 mice (6–8 weeks old) obtained from Spiff Biotechnology (Beijing, China) were housed in the SPF-grade breeding environment, keeping temperature (21–23 °C) and humidity (60–65%). The mice had ad libitum access to food and water and were maintained on a 12 h light/dark cycle. After one week of acclimatization, experiments were conducted. All procedures were approved by the Ethics Committee of The First Affiliated Hospital of Nanchang University, Jiangxi Medical College.

### Animal model establishment and experimental procedures

Mice were subcutaneously injected with Hepa1-6 cells in the right flank to establish HCC xenograft models. In the knockdown studies, mice were divided into two groups. Mice received injection of Hepa1-6 cells transfected with shRNA negative control (sh-NC) in the sh-NC group, while those in the experimental group received injection of Hepa1-6 cells with STT3A knockdown (sh-STT3A). For overexpression experiments, three treatment conditions were established. Mice were administered with empty vector control (OE-NC)-transfected Hepa1-6 cells in the OE-NC control group. Mice received injection of STT3A-overexpressing (OE-STT3A) Hepa1-6 cells in the OE-STT3A group. The OE-STT3A + DT combination treatment group received STT3A-overexpressing cells followed by diphtheria toxin (DT, #D0564, Sigma-Aldrich, St. Louis, MO, USA) treatment. The DT regimen consisted of intraperitoneal injections at 6.25 µg/kg administered weekly for two consecutive weeks, with the first dose given one week after cell inoculation. All mice were humanely anesthetized and euthanized, whereupon tumor dimensions (images, volumes and weights) were documented and both tumor specimens and pulmonary tissues were harvested for subsequent analysis.

### Hematoxylin and eosin (H&E) staining

The collected lung tissues were fixed in 4% paraformaldehyde followed by dehydration and clearing. Tissues were then embedded in paraffin and sectioned at 4 μm thickness. For H&E staining, sections were deparaffinized with xylene substitute and rehydrated through decreasing ethanol concentrations. Nuclei were stained with hematoxylin (#G1004, Servicebio, Wuhan, Hubei, China) for 5 min followed by 1 min washing and 15 s differentiation. After 5 min of eosin (#G1001, Servicebio) counterstaining, sections were dehydrated, cleared and mounted with neutral balsam (#WG10004160, Servicebio). Stained sections were scanned and imaged using a Keyence BZ-X800 fluorescence microscope.

### Immunohistochemistry (IHC) analysis

Paraffin-embedded clinical tissue sections were deparaffinized and rehydrated. Antigen retrieval was performed by microwave heating in EDTA buffer (#P0085, Beyotime, Shanghai, China). After peroxidase blocking, sections were incubated with primary antibodies against FCN3 (#11867-1-AP, 1:200; Proteintech, Wuhan, Hubei, China) or STT3A (#12034-1-AP, 1:200; Proteintech) at 37 °C for 30 min. The PV-6000 universal detection system (Zhongshan Golden Bridge, Beijing, China) was used for secondary antibody incubation, followed by DAB (#G1212, Servicebio) development. Counterstaining was performed with hematoxylin for 2 min. Sections were examined using a Leica DM500 microscope.

### Cell culture and treatment

Human normal hepatocyte THLE-2 and HCC cell lines (HepG2, Hep3B, HCC-LM3) were purchased from Immocell Biotechnology (Xiamen, Fujian, China), cultured under standard conditions (37 °C, 5% CO_2_) in their respective media. THLE-2 specific medium was used for normal hepatocytes while DMEM (high glucose) supplemented with 10% FBS and 1% penicillin/streptomycin was employed for HCC cell lines. Preliminary screening identified Hep3B with low endogenous FCN3 expression and HCC-LM3 with high FCN3 expression as experimental models. For transfection experiments, cells at 70–80% confluency in 6-well plates were transfected with 2.5 µg pcDNA3.1(+) plasmid DNA using Lipo8000 transfection reagent (#C0533, Beyotime) according to the manufacturer’s protocol. Stable cell lines were established through lentiviral transduction including FCN3 wild-type (FCN3-WT), FCN3 N189Q mutant (FCN3-N189Q), sh-NC, FCN3 knockdown (sh-FCN3), sh-STT3A, OE-NC and FCN3 overexpression (OE-FCN3) models. Pharmacological interventions involved treatment with either the β-catenin activator LY2090314, which activates the Wnt/β-catenin signaling pathway by inhibiting GSK-3 activity [28], or the N-glycosylation inhibitor tunicamycin (TM).

To investigate the effects and mechanisms of FCN3 in HCC, the following experimental treatments were conducted: Hep3B cells were subjected to OE-NC, OE-FCN3, OE-FCN3 + DMSO or OE-FCN3 + LY2090314 groups, while HCC-LM3 cells received either sh-NC or sh-FCN3 treatments. For FCN3 glycosylation studies, Hep3B and HCC-LM3 cells with FCN3 knockout were transfected with Vector, FCN3-WT or FCN3-N189Q. Additionally, to examine the role of STT3A, Hep3B cells were divided into sh-NC and sh-STT3A groups. All genetic and pharmacological manipulations included appropriate control conditions.

### Co-culture system

Peripheral blood mononuclear cells (PBMCs, #CM-H182, Procell, Wuhan, Hubei, China) were cultured in complete PBMC medium (#CM-H158, Procell) for 48 h prior to co-culture experiments. HCC cells (transfected with respective constructs as described previously) were simultaneously prepared under standard conditions. For indirect co-culture, HCC cells were seeded in the upper chamber of Transwell inserts while PBMCs were plated in the lower chamber at a 1:1 ratio, allowing bidirectional cytokine communication without direct cell contact. The co-culture system was maintained for 96 h in a humidified CO₂ incubator at 37 °C with 5% CO₂. Post-incubation, PBMCs were collected for flow cytometry assessment of T cell activation.

### Immunofluorescence (IF) analysis

For clinical tissue samples, paraffin-embedded sections were deparaffinized and rehydrated, followed by antigen retrieval. After permeabilization with 0.2% Triton X-100 and blocking with 3% BSA, sections were incubated with β-catenin primary antibody (#51067-2-AP, 1:200; Proteintech) at 4 °C overnight. After thorough washing, the sections were then probed with Cy3-conjugated secondary antibody (#A0516, 1:200; Beyotime) for 1 h at room temperature. Nuclei were counterstained with DAPI (#G1012, Servicebio).

For cell-based assays, HCC cell lines (HepG2, Hep3B, HCC‑LM3) and normal hepatocytes were fixed with 4% paraformaldehyde and permeabilized with 0.1% Triton X-100. After blocking, cells were stained with β-catenin antibody (#51067-2-AP, 1:100; Proteintech) followed by FITC-conjugated secondary antibody (#A0568, 1:100; Beyotime). For FCN3 staining, HCC‑LM3 and Hep3B cells were similarly processed and probed with FCN3 primary antibody (#11867‑1‑AP, 1:200; Proteintech) overnight at 4 °C, followed by incubation with Cy3‑conjugated goat anti‑rabbit IgG (#A0516, 1:100; Beyotime). Nuclei were counterstained with DAPI (#C1006, Beyotime). Images were acquired using a Keyence BZ-X800 fluorescence microscope.

### Flow cytometry analysis

For apoptosis detection, cells were harvested at 80–90% confluence using EDTA-free trypsin and washed twice with ice-cold PBS. Cell pellets were resuspended in binding buffer (5 × 10⁶ cells/mL), then stained with 5 µL Annexin V-FITC and 5 µL propidium iodide from Annexin V-FITC apoptosis kit (#G1511, Servicebio) for 10 min at room temperature protected from light. Samples were diluted with binding buffer and analyzed immediately on a flow cytometer (#RMNNC-3000, Agilent, Santa Clara, CA, USA) to quantify apoptotic populations.

For Treg cell analysis, PBMCs from co-culture experiments were processed similarly with modifications for staining. After PBS washes, 5 × 10⁵ cells were stained with anti-CD4-FITC (#980802, BioLegend, San Diego, CA, USA), anti-CD25-APC (#985810, BioLegend) and anti-FOXP3-PE (#320107, BioLegend) antibodies in binding buffer for 30 min on ice. Following washes, cells were analyzed on a NovoCyte system (Agilent).

### Cell counting kit-8 (CCK-8) assay

Cell viability was assessed using the CCK-8 kit (#40203ES60, Yeasen, Shanghai, China) according to the manufacturer’s protocol. Briefly, cells were trypsinized and adjusted to a density of 1 × 10⁶ cells/mL. Cell suspensions were seeded into 96-well plates at 2,000 cells per well. After 24 h of incubation at 37 °C with 5% CO₂, 10 µL of CCK-8 reagent was added to each well followed by additional 1 h incubation under light-protected conditions. Absorbance was measured at 450 nm using a Multiskan FC microplate reader (Thermo Fisher Scientific, Waltham, MA, USA).

### Wound healing assay

Cell migration capacity was evaluated using a standardized scratch wound healing assay. The 6-well plates were pre-marked with three parallel reference lines on the outer bottom surface using a marker pen and ruler. Cells were seeded at 2 × 10⁵ cells/well and cultured overnight to form confluent monolayers. A uniform scratch wound was created in each well using a 200 µL pipette tip held perpendicular to the plate surface to ensure straight-edged wounds. After washing three times with PBS to remove dislodged cells, serum-free medium was added to eliminate proliferation interference. The plates were then incubated at 37 °C with 5% CO₂. Wound closure was monitored and photographed at 0 and 24 h time points using a Keyence BZ-X800 inverted fluorescence microscope. Migration distances were quantified using ImageJ software.

### Transwell assay

Cell invasion capacity was evaluated using Matrigel-coated Transwell chambers (#4395, BD Biosciences, San Jose, CA, USA). Matrigel matrix (#356234, BD Biosciences) was thawed overnight at 4 °C and diluted with serum-free medium. The diluted Matrigel solution was uniformly coated onto the upper chambers of 24-well Transwell plates and allowed to polymerize at 37 °C for 3 h. Cells were trypsinized, washed and resuspended in serum-free medium. 100 µL cell suspension was added to the upper chamber while 600 µL complete medium containing 10% FBS was added to the lower chamber. After 24 h incubation at 37 °C with 5% CO₂, non-invaded cells on the upper chamber surface were removed with cotton swabs. Invaded cells on the lower chamber were fixed with 4% paraformaldehyde for 30 min and stained with 0.1% crystal violet (#G1014, Servicebio) for 20 min. Transwell plates were washed three times with PBS, air-dried and photographed under a Keyence BZ-X800 inverted microscope. Invaded cells were counted using ImageJ software.

### Quantitative real-time fluorescence PCR (qRT-PCR)

Total RNA was extracted from HCC clinical samples, tumor tissues or cells using TRIzol (#G3013, Servicebio) and reverse-transcribed into cDNA with PrimeScript RT Kit (#RR037Q, Takara, Tokyo, Japan). qPCR was performed using TB Green Premix (#RR820Q, Takara) on a SLAN-96 S system (Hongshi Medical Technology, Shanghai, China). After each reaction was repeated three times, the mRNA levels of FCN3, FOXP3, CD25, APC, β-catenin and STT3A were calculated using 2^−∆∆CT^ method. The primers used in qRT-PCR were listed in Table 1.

**Table 1:** Primers of qRT-PCR

Gene	Forward sequence (5’-3’)	Reverse sequence (5’-3’)
FCN3 Human	TCCTCGGTGAGGTAGACCACTA	GCTGTTGCTTGAATCGTGGTCAG
FOXP3 Human	GCTGCAGCTCTCAACGGT	AGGCAAACATGCGTGTGAAC
FOXP3 Mouse	GGCAGAGAGGTATTGAGGGTG	CTTTCTTCTGTCTGGAGTGGC
CD25 Human	CGTCTGCAAAATGACCCACG	AGCACAACGGATGTCTCCTG
CD25 Mouse	AAGCCAAATCTGAGACGCCA	TTGGGCTTTCCAAACTGGGT
APC Human	AGACAGAATGGAGGTGCTGC	CTTCGAGGTGCAGAGTGTGT
APC Mouse	AGACAGAATGGAGGTGCTGC	CCCTGATCTGCCTTGCTTCA
β-catenin Human	GGCTACTCAAGCTGATTTGATGG	ATTGCACGTGTGGCAAGTTC
β-catenin Mouse	GGCGGCCGCGAGGTA	CTGGGATGCCACCAGACTTA
STT3A Human	CAGCTGCAAACCCTGAGAGA	ACAGGCTGGAAACCCACAAA
STT3A Mouse	TGAGGAGCATCCGTGAGGTA	CGGGCTGGAAACCAACAAAG
β-actin Human	CACCATTGGCAATGAGCGGTTC	AGGTCTTTGCGGATGTCCACGT
β-actin Mouse	TGAGCTGCGTTTTACACCCT	AAGTCAGTGTACAGGCCAGC

### Western blot (WB)

Protein lysates were extracted from tissues or cells using RIPA buffer supplemented with protease inhibitors. After centrifugation, supernatants were quantified by BCA assay (#BL521A, BioSharp, Guangzhou, Guangdong, China). Samples were separated on SDS-PAGE gels and transferred to PVDF membranes (#IPVH00010, Millipore, Billerica, MA, USA). After blocking with 5% skim milk for 2 h at room temperature, membranes were incubated overnight at 4 °C with primary antibodies against FCN3 (#ABIN521997, 1:2000; Antibodies Online, Aachen, Germany), FOXP3 (#22228-1-AP, 1:500; Proteintech), CD25 (#83896-1-RR, 1:1000; Proteintech), APC (#sc-9998, 1:500; Santa Cruz Biotechnology, Santa Cruz, CA, USA), β-catenin (#66379-1-Ig, 1:5000; Proteintech), phosphorylated β-catenin (p-β-catenin) (#80067-1-RR, 1:1000; Proteintech) or STT3A (#ab320831, 1:20000; Abcam, Cambridge, MA, USA). After PBST washes, HRP-conjugated secondary antibodies (anti-mouse, #AB0102 or anti-rabbit, #AB0101, 1:5000; Abways, Shanghai, China) were applied for 2 h at 37 °C. Protein bands were visualized using chemiluminescent HRP substrate (#WBKLS0100, Millipore) on a ChampChemi 910 system (SINSAGE, Beijing, China). β-actin (#66009-1-Ig, 1:20000; Proteintech) served as the loading control for whole‑cell lysates. For subcellular localization analysis, Lamin B1 (#12987‑1‑AP, 1:5000; Proteintech) was used as the nuclear loading control.

### Glycosylation analysis of FCN3

For N-glycosidase/O-glycosidase treatment, protein lysates from HCC-LM3 and Hep3B cells were extracted and subjected to deglycosylation using either PNGase F (#P0704S, New England BioLabs, Ipswich, MA, USA) for N-glycosylation removal or O-glycosidase (#P0733, New England BioLabs) for O-glycosylation removal following manufacturer’s instructions. WB analysis was then performed as described above. The mobility shift of FCN3 was analyzed to determine glycosylation patterns.

For TM inhibition assay, Hep3B cells were treated with 2 µg/mL TM (#B7417, ApexBio Technology, Houston, Texas, USA) or DMSO control for 24 h. Cell lysates were prepared and analyzed by WB following standard procedures. The band shift pattern of FCN3 was compared between TM-treated and control groups to confirm N-glycosylation modification.

### Site-directed mutagenesis of FCN3

To validate the glycosylation site of FCN3, primers synthesized by Tsingke Biotechnology (Beijing, China) were used to construct FCN3-WT and FCN3-N189Q plasmids. Following sequence verification, the FCN3-WT and FCN3-N189Q constructs were transfected into HCC cells and cultured for 24 h. The stability and function of FCN3 with Asn189 site mutations were analyzed by subsequent experiments. The sequences utilized were as follows: FCN3-WT: AGCTGGAAGACTTTAATGGTAACCGTACTTTCGCCCACTATGC; FCN3-N189Q: AGCTGGAAGACTTTAATGGTCAACGTACTTTCGCCCACTATGC.

### Co-immunoprecipitation (Co-IP) assay

Cell lysates were prepared using RIPA buffer containing protease inhibitors. For the assessment of interactions between FCN3 and STT3A, APC or β‑catenin, 200 µg protein per sample was incubated with antibody-conjugated Protein A/G magnetic beads (#P2179S, Beyotime) overnight at 4 °C with rotation. Specifically, anti‑FCN3 (#ABIN521997, Antibodies Online), anti‑APC (#sc‑9998, Santa Cruz Biotechnology), anti‑β‑catenin (#66379‑1‑Ig, Proteintech), or anti‑STT3A (#ab320831, Abcam) antibody was used for the respective immunoprecipitations. For STT3A‑FCN3 interaction analysis, either anti‑STT3A or anti‑FCN3 antibody‑coupled beads were employed. IgG (#P2179S-5, mouse or #P2179S-6, rabbit) isotype controls were included for specificity verification. After extensive washing, bound proteins were eluted and analyzed by WB following standard procedures.

### Enzyme-linked immunosorbent assay (ELISA)

Immunosuppressive cytokine levels were quantified using commercial ELISA kits (MultiSciences, Hangzhou, Zhejiang, China) including TGF-β1 ELISA kit (#EK981) and IL-10 ELISA kit (#EK210) according to the manufacturer’s protocol. For detection of secreted FCN3, the Human FCN3 ELISA Kit (#CSB‑E13104h, Cusabio, Wuhan, Hubei, China) was used following the supplier’s instructions. Briefly, 100 µL of standards or samples were added to pre-coated plates and incubated at 37 °C for 90 min. After incubation with biotinylated detection antibody (1 h, 37 °C) and HRP-streptavidin (30 min, 37 °C), TMB substrate was added for 15 min at 37 °C protected from light. Reactions were stopped with 50 µL stop solution, and absorbance was measured at 450 nm using a Multiskan FC microplate reader.

### Statistical analysis

Each assay was performed for 3 times. Data were analyzed by GraphPad Prism 8.0 (La Jolla, CA, USA) and expressed as mean ± standard deviation. Two-tailed Student’s *t* test were used for comparing two variables. One-way ANOVA test was used for multiple variable comparison. *P* < 0.05 was considered as a significant difference.

## Results

### Downregulation of FCN3 in HCC promoted Treg cell activation

**Fig. 1:**
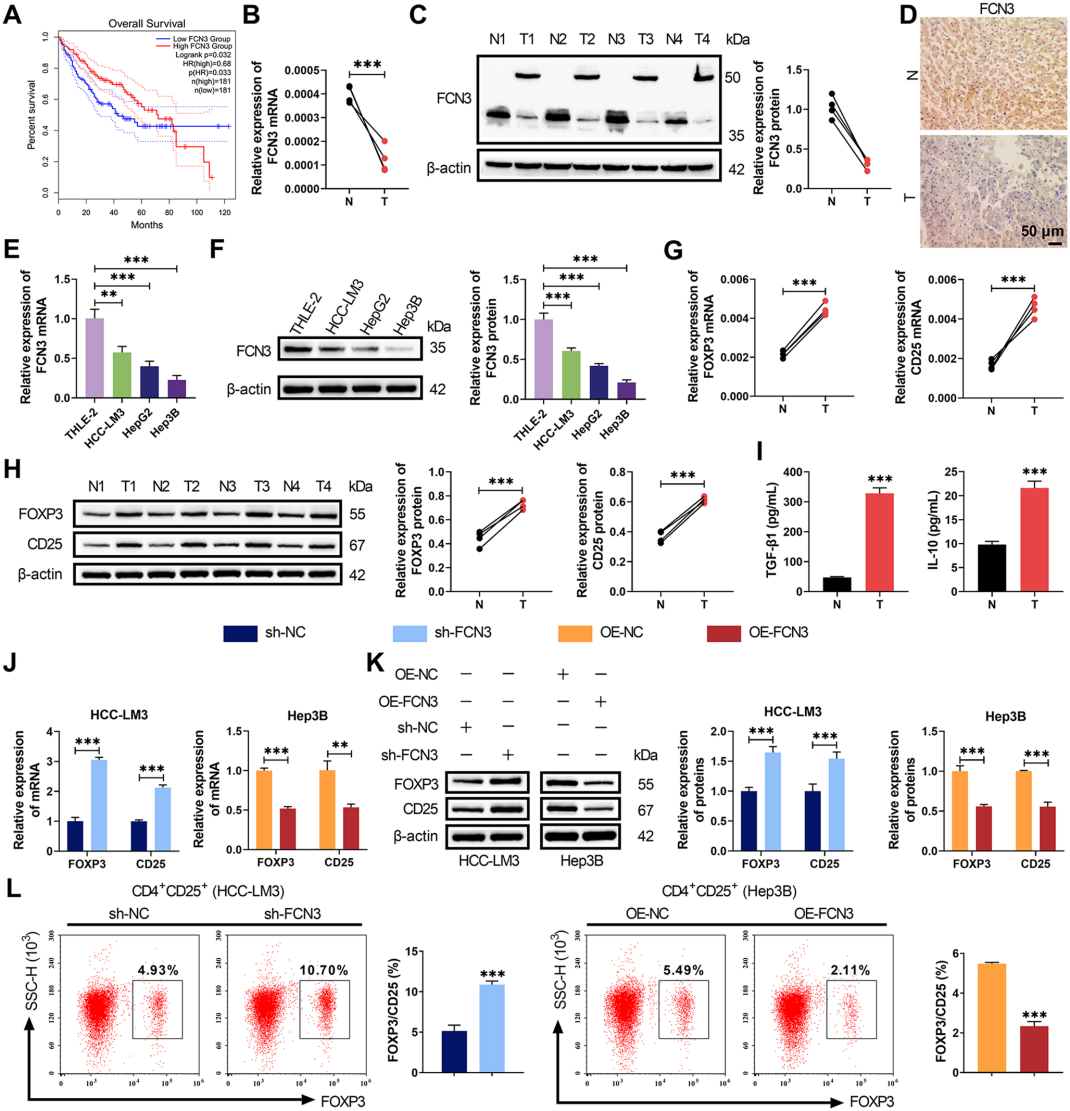
Low FCN3 expression in HCC affected Treg cell activation. (**A**) Kaplan-Meier survival analysis showing the correlation between FCN3 levels and overall survival of HCC patients. (**B**-**D**) qRT-PCR, WB and IHC analysis of FCN3 expression in tumor (**T**) and adjacent non-tumor (**N**) tissues from HCC patients. (**E**-**F**) The levels of FCN3 in normal hepatocyte THLE-2 and HCC cell lines (HepG2, Hep3B, HCC-LM3) were assessed by qRT-PCR and WB. (**G**-**H**) qRT-PCR and WB analysis demonstrating the mRNA and protein levels of Treg cell markers FOXP3 and CD25 in clinical samples. (**I**) ELISA quantification of TGF-β1 and IL-10 in HCC tumor and adjacent non-tumor tissues. (**J**-**K**) The levels of FOXP3 and CD25 in PBMCs co-cultured with FCN3-knockdown or FCN3-overexpressing HCC cells were evaluated by qRT-PCR and WB. (**L**) Flow cytometry analysis of CD4⁺CD25⁺FOXP3^+^ Treg cell proportion in PBMCs within the co-culture systems. ^***^p < 0.001, ^**^p < 0.01, ^*^p < 0.05 vs. N/ THLE-2/ sh-NC/ OE-NC

Patients with high FCN3 expression exhibited significantly longer overall survival compared to those with low FCN3 expression, suggesting a prognostic value of FCN3 in HCC (Fig. 1A). Clinical sample analysis revealed markedly reduced FCN3 expression in HCC tumor tissues compared to adjacent non-tumor tissues (Fig. 1B-D). Of note, WB analysis in clinical samples showed not only decreased FCN3 expression but also the presence of a higher molecular weight band in tumor tissues, indicative of glycosylated FCN3 (Fig. 1C). qRT-PCR and WB further confirmed lower FCN3 levels in HCC cell lines (HepG2, Hep3B, HCC-LM3) than in normal hepatocytes THLE-2 (Fig. 1E-F). Notably, HCC tissues showed elevated expression of Treg cell markers (FOXP3, CD25) and immunosuppressive cytokines (TGF-β1, IL-10) (Fig. 1G-I). To investigate FCN3’s role in Treg cell activation, co-culture systems with PBMCs were established using HCC-LM3 cells with FCN3 knockdown or Hep3B cells with FCN3 overexpression. The transfection efficiency was verified in Figure S1A-D. FCN3 knockdown in HCC-LM3 cells upregulated FOXP3 and CD25 expression and enhanced Treg cell activation, whereas FCN3 overexpression in Hep3B cells produced opposite effects (Fig. 1J-L). These findings demonstrated that reduced FCN3 expression in HCC promoted Treg cell activation.

### FCN3 overexpression suppressed Treg cell activation in HCC by inhibiting Wnt/β-catenin signaling

**Fig. 2:**
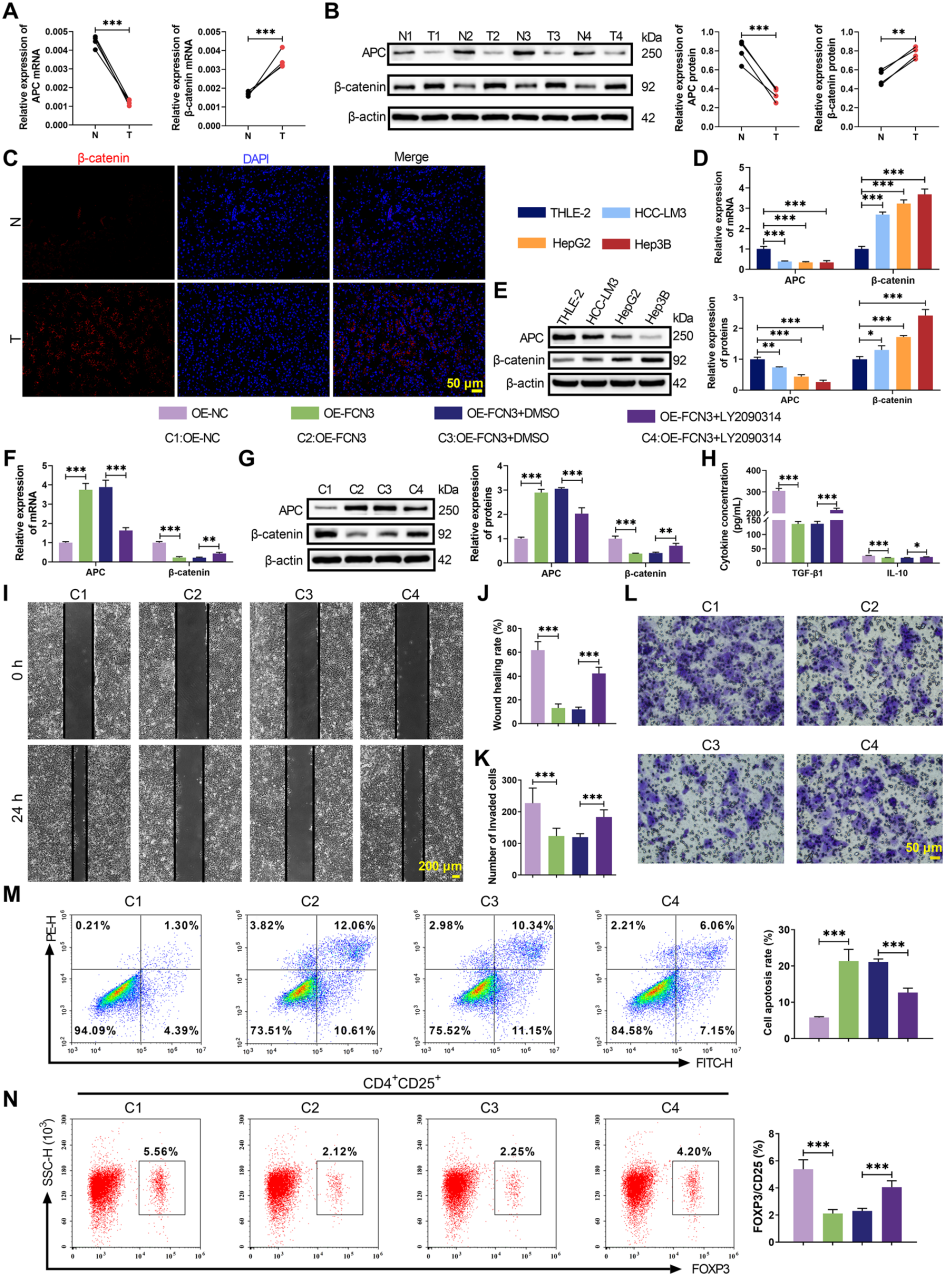
FCN3 regulated Wnt/β-catenin signaling to influence Treg activation. (**A**-**B**) qRT-PCR and WB analysis of APC and β-catenin in HCC tumor and adjacent non-tumor tissues. (**C**) Clinical sample IF staining showing β-catenin expression and localization with DAPI nuclear counterstain. (**D**-**E**) The levels of APC and β-catenin in HCC cell lines were assessed by qRT-PCR and WB. (**F**-**G**) qRT-PCR and WB evaluation of APC and β-catenin in Hep3B cells treated with OE-NC, OE-FCN3, OE-FCN3 + DMSO, or OE-FCN3 + LY2090314 (β-catenin activator). (**H**) The levels of TGF-β1 and IL-10 in cell culture supernatant of each group were examined by ELISA. (**I**-**M**) Wound healing, Transwell and flow cytometry assays evaluating the effect of OE-FCN3 and OE-FCN3 + LY2090314 on cell migration, invasion and apoptosis of Hep3B cells. (**N**) Flow cytometry was performed to analyze the effects of OE-FCN3 and OE-FCN3 + LY2090314 treatment in Hep3B cells on the proportion of CD4⁺CD25⁺FOXP3^+^ Treg cells in Hep3B-PBMC co-cultures. ^***^p < 0.001, ^**^p < 0.01, ^*^p < 0.05 vs. N/ THLE-2/ OE-NC/ OE-FCN3 + DMSO

Compared to adjacent non-tumor tissues, HCC specimens exhibited increased β-catenin and decreased APC expression (Fig. 2A-C). Similarly, HCC cell lines showed lower APC and higher β-catenin levels than normal hepatocytes (Fig. 2D-E, S2E). GO analysis and GeneMANIA prediction indicated a positive correlation between FCN3 and APC, a key regulator of Wnt pathway activation (Figure S2A, S2C). Moreover, correlation analysis in clinical HCC samples revealed a positive relationship between FCN3 and APC protein expression, while an inverse association was observed between FCN3 and β-catenin (Figure S2B). CO‑IP assay in normal hepatocytes showed no direct physical interaction between FCN3 and either APC or β‑catenin (Figure S2D). To elucidate the impact of FCN3 on Wnt/β-catenin signaling and HCC cells, FCN3-overexpressing Hep3B cells were treated with or without the β-catenin activator LY2090314. FCN3 overexpression suppressed β-catenin and restored APC expression in Hep3B cells, which were reversed by LY2090314 treatment (Fig. 2F-G, S2F). ELISA confirmed that FCN3 overexpression reduced TGF-β1 and IL-10 secretion in Hep3B cells, which was partially counteracted by LY2090314 (Fig. 2H). Functional assays demonstrated that LY2090314 attenuated FCN3 overexpression-mediated the suppression of cell viability, migration, and invasion and the enhancement of apoptosis (Fig. 2I-M, S2G). Co-culture experiments further revealed that FCN3 overexpression in Hep3B cells reduced the expression of FOXP3 and CD25 and the activation of Treg cells, which were impeded by LY2090314 (Fig. 2N, S2H-I). These results indicated that FCN3 overexpression-mediated inhibition of Wnt/β-catenin signaling modulated HCC cell functions and Treg cell activation.

### Enhanced N-glycosylation at Asn189 of FCN3 in HCC

**Fig. 3:**
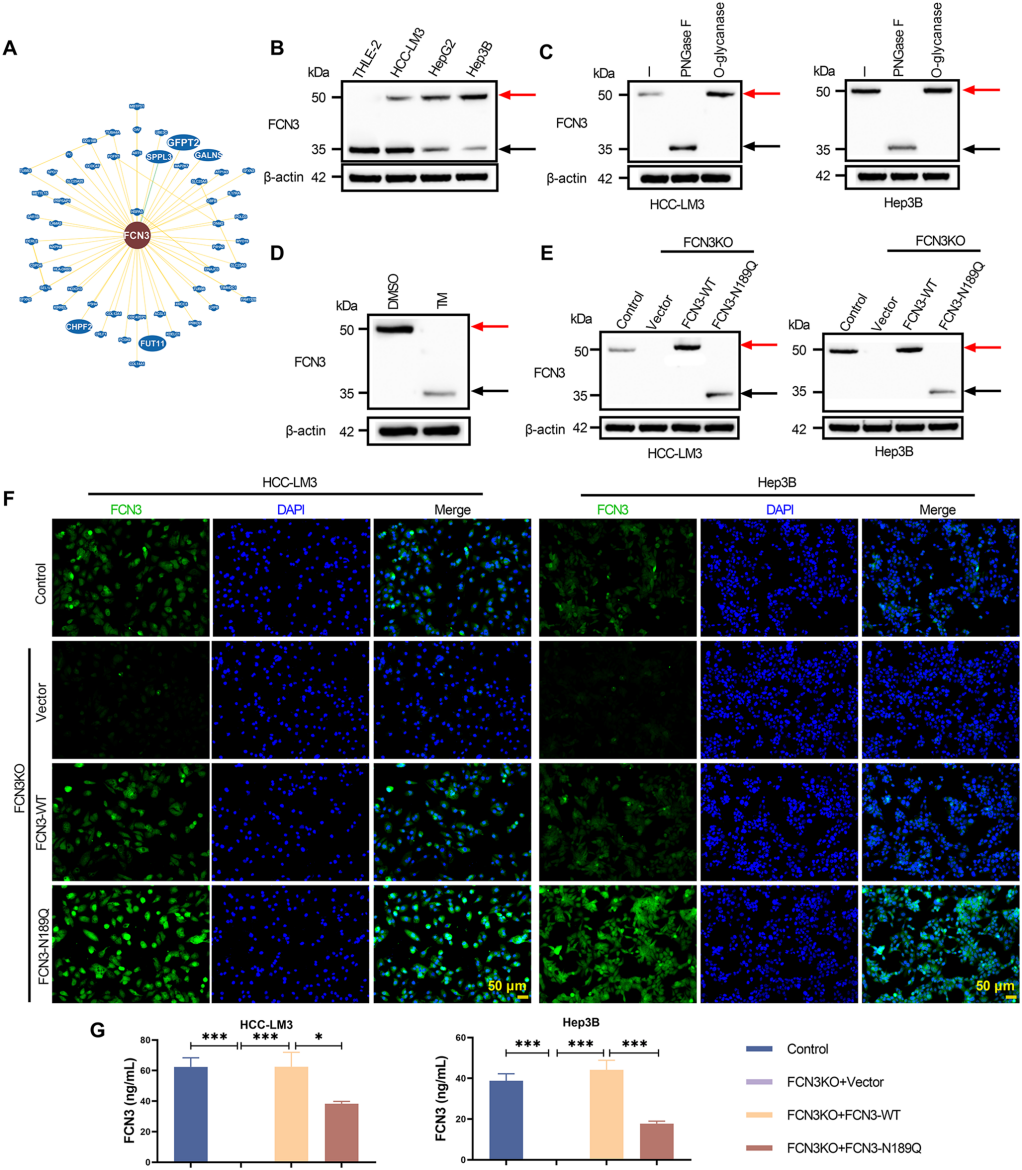
Enhanced N-glycosylation of FCN3 in HCC. (**A**) Protein-protein interaction network obtained from BioGRID Database exhibited the connection between FCN3 and glycosylation-related proteins. (**B**) WB assay confirmed FCN3 band shift in HCC cell lysates, showing FCN3 galactosylated modification. (**C**) WB analysis of HCC cell lysates treated with PNGase F or O-Glycanase was performed to verify that FCN3 glycosylation was N-linked rather than O-linked. (**D**) FCN3 band shift in Hep3B cells treated with TM or DMSO was analyzed by WB. (**E**) WB analysis of FCN3 protein was performed in FCN3-knockdown HCC cells transfected with either Vector, FCN3-WT, or FCN3-N189Q. (**F**-**G**) IF staining and ELISA quantification showed the effects of the Asn189 mutation on FCN3 intracellular levels and its secretion into the culture supernatant, respectively

Protein interaction network analysis revealed associations between FCN3 and multiple glycosylation-related proteins (Fig. 3A). WB showed altered FCN3 banding patterns in HCC cells compared to normal hepatocytes (Fig. 3B), suggesting potential glycosylation modifications of FCN3. Treatment with PNGase F (but not O-Glycanase) eliminated the band shift in HCC-LM3 and Hep3B cells, confirming N-glycosylation of FCN3 in HCC (Fig. 3C). This was further supported by suppressed FCN3 glycosylation in Hep3B cells treated with the N-glycosylation inhibitor TM (Fig. 3D). Moreover, mutation of the Asn189 site abolished FCN3 glycosylation in both HCC-LM3 and Hep3B cells (Fig. 3E), identifying Asn189 as the critical glycosylation site. IF staining revealed stronger intracellular FCN3 signal in the FCN3‑N189Q group compared to FCN3‑WT (Fig. 3F). Correspondingly, ELISA of culture supernatants showed lower secreted FCN3 level in the FCN3‑N189Q group (Fig. 3G). These results suggested that N-glycosylation at Asn189 promoted FCN3 extracellular secretion.

### N-glycosylation of FCN3 at Asn189 activated Wnt/β-catenin signaling, promoted Treg cell activation and drove HCC progression

**Fig. 4:**
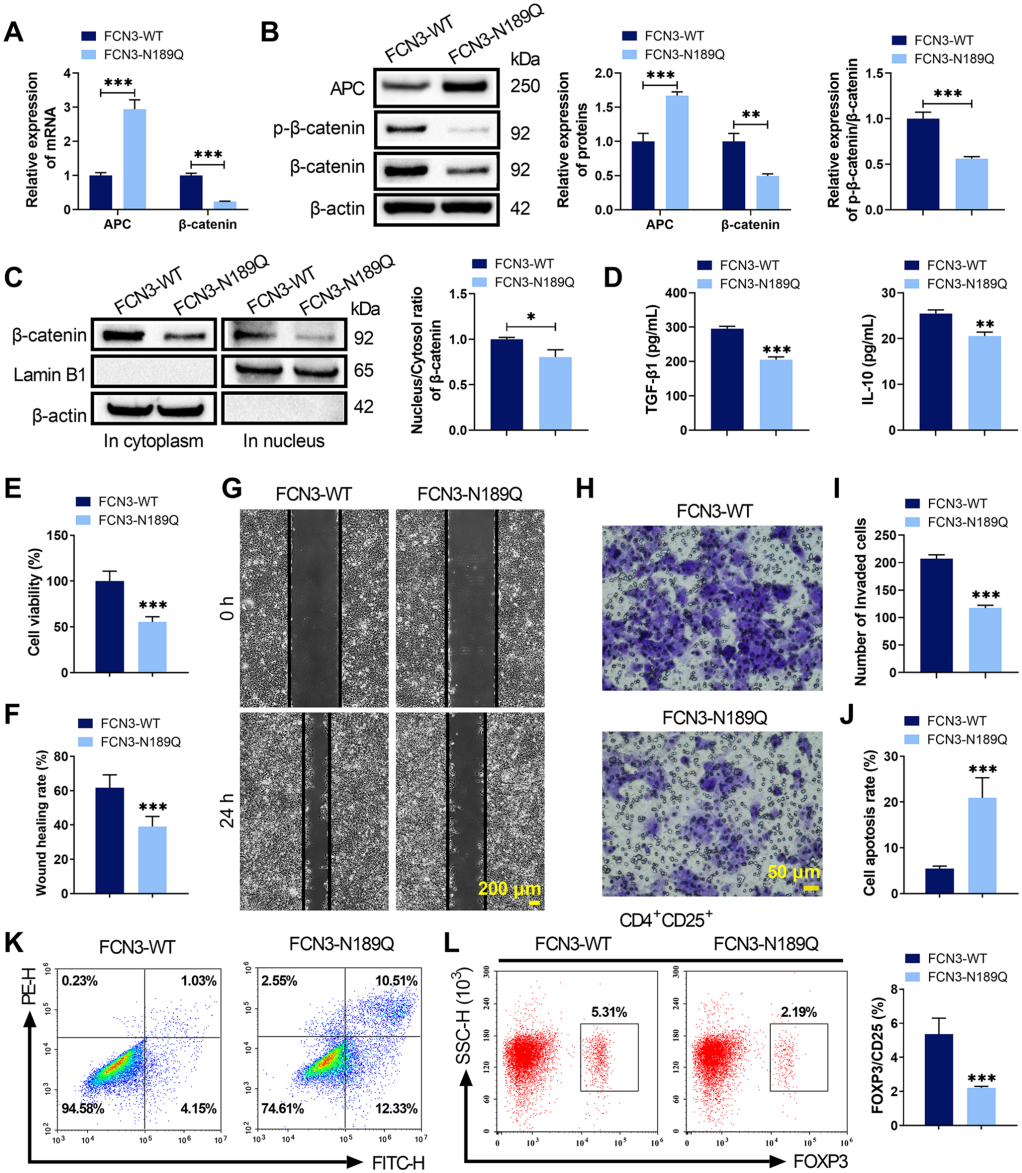
N-glycosylated FCN3 influenced Wnt/β-catenin signaling and Treg activation. (**A**) qRT-PCR analysis of APC and β-catenin mRNA levels in Hep3B cells transfected with FCN3-WT or FCN3-N189Q. (**B**) WB analysis showing the levels of APC, β-catenin, and p-β-catenin. (**C**) Subcellular localization of β-catenin was analyzed by WB. (**D**) ELISA was conducted to assess the levels of TGF-β1 and IL-10 in cell culture supernatant of Hep3B cells transfected with FCN3-WT or FCN3-N189Q. (**E**-**K**) The effects of FCN3 glycosylation on cell viability, migration, invasion and apoptosis in Hep3B cells were respectively evaluated by CCK-8, wound healing, Transwell and flow cytometry assays. (**L**) Flow cytometry demonstrating the impact of FCN3 glycosylation on the proportion of CD4⁺CD25⁺FOXP3^+^ Treg cells in Hep3B-PBMC co-cultures. ^***^p < 0.001, ^**^p < 0.01, ^*^p < 0.05 vs. FCN3-WT

To investigate the functional significance of FCN3 glycosylation at Asn189, Hep3B cells were transfected with either FCN3-WT or FCN3-N189Q. Compared to FCN3-WT, the N189Q mutant led to a significant reduction in β-catenin, p‑β‑catenin, and the p‑β‑catenin/β‑catenin ratio while increasing APC expression (Fig. 4A-B). Furthermore, WB assay showed that the N189Q mutation markedly reduced β‑catenin nuclear translocation (Fig. 4C). The N189Q mutant also decreased TGF-β1 and IL-10 secretion compared to FCN3‑WT (Fig. 4D). Functional assays demonstrated that Hep3B cells with N189Q-mutated FCN3 exhibited reduced viability, migration and invasion, along with increased apoptosis (Fig. 4E-K). Furthermore, co-culture of Hep3B and PBMCs revealed that N189Q-mutated FCN3 caused lower Treg cell activation than FCN3-WT (Fig. 4L). These results indicated that N-glycosylation of FCN3 at Asn189 facilitated Wnt/β-catenin signaling activation, β‑catenin nuclear translocation and Treg cell activation, thereby promoting HCC progression.

### STT3A drove Wnt signaling activation and Treg cell activation in HCC by promoting N-glycosylation of FCN3

**Fig. 5:**
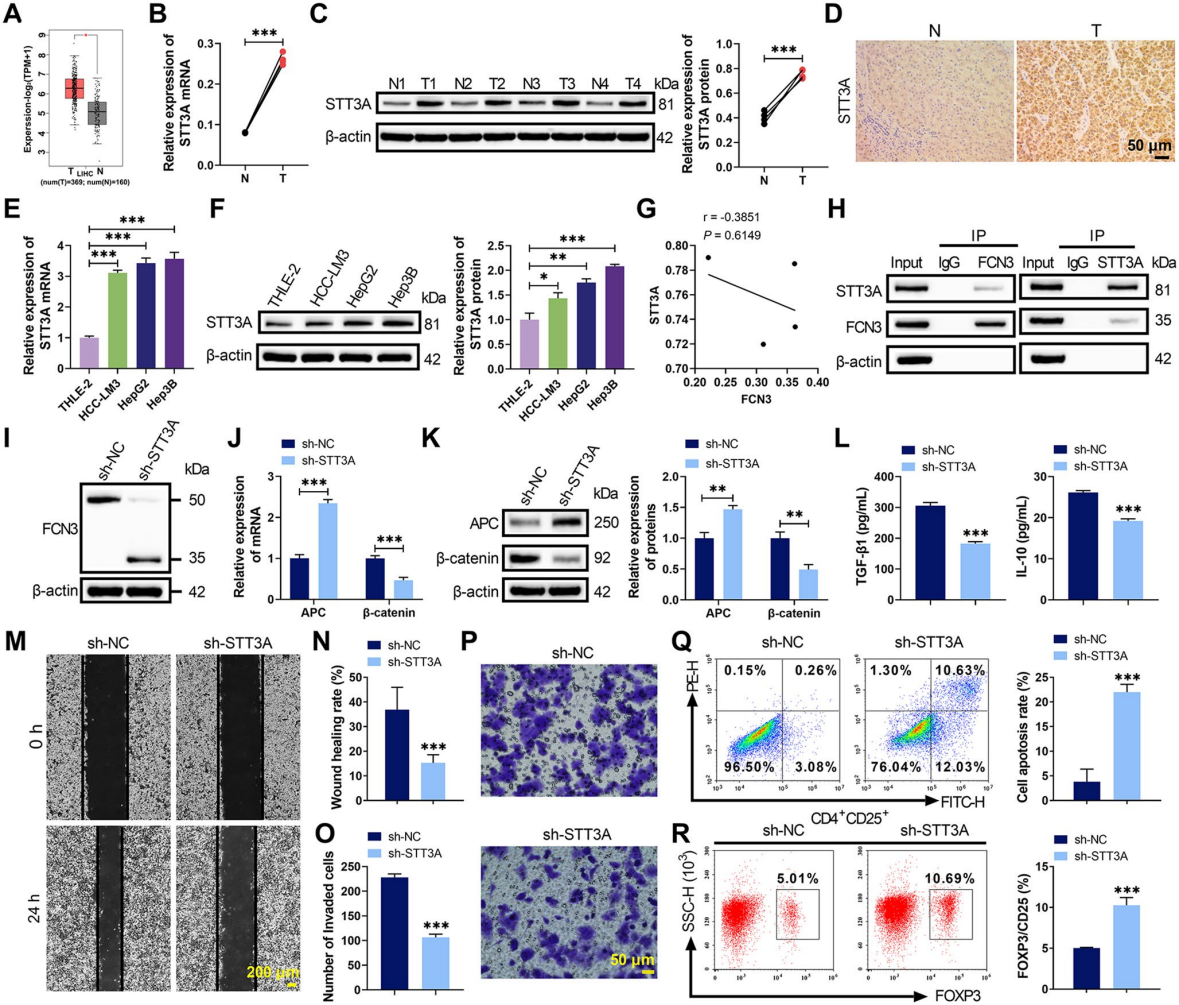
STT3A involved in HCC progression via promoting FCN3 N-glycosylation. (**A**) Bioinformatics analysis revealing STT3A expression in normal and HCC liver tissues. (**B**-**D**) qRT-PCR, WB and IHC assays were performed to assess STT3A levels in HCC tumor and adjacent non-tumor tissues. (**E**-**F**) The mRNA and protein levels of STT3A in normal hepatocyte and HCC cell lines were analyzed by qRT-PCR and WB. (**G**) Correlation analysis between STT3A protein expression and FCN3 protein expression in tumor tissues from HCC patients. (**H**) Co-IP assay verified STT3A-FCN3 interaction. (**I**) WB analysis of FCN3 glycosylation in Hep3B cells transfected with sh-STT3A or sh-NC. (**J**-**K**) The effects of sh-STT3A on the expression of APC and β-catenin in Hep3B cells were examined by qRT-PCR and WB. (**L**) ELISA showing TGF-β1 and IL-10 levels in Hep3B cell supernatants following sh-STT3A or sh-NC treatment. (**M**-**Q**) Wound healing, Transwell and flow cytometry assays were conducted to evaluate the effects of sh-STT3A on Hep3B cell migration, invasion and apoptosis. (**R**) The influence of sh-STT3A on the proportion of CD4⁺CD25⁺FOXP3^+^ Treg cells in Hep3B-PBMC co-cultures was assessed by flow cytometry. ^***^p < 0.001, ^**^p < 0.01, ^*^p < 0.05 vs. N/ THLE-2/ sh-NC

Bioinformatics analysis revealed a complex regulatory network among STT3A, FCN3, APC and β-catenin, along with significantly elevated STT3A levels in HCC (Fig. 5A, S3A). Clinical sample and cell line experiments confirmed higher STT3A mRNA and protein levels in HCC tissues (Fig. 5B-D) and cell lines (Fig. 5E-F) compared to normal controls. Correlation analysis in clinical tumor tissues further showed that STT3A protein levels were inversely associated with total FCN3 expression (Fig. 5G), suggesting a link between STT3A expression and FCN3 regulation. Co-IP verified direct interaction between STT3A and FCN3 (Fig. 5H). To determine whether STT3A regulated Wnt signaling and Treg cells through FCN3 glycosylation, STT3A knockdown was conducted and verified in Hep3B cells (Figure S3B-D). STT3A knockdown reduced FCN3 glycosylation (Fig. 5I), suppressed β-catenin, enhanced APC expression (Fig. 5J-K, S3E) and decreased TGF-β1 and IL-10 secretion (Fig. 5L). Additionally, STT3A knockdown impaired Hep3B cell viability, migration, and invasion (Fig. 5M-P, S3F), while promoting its apoptosis (Fig. 5Q). Co-culture experiments showed that STT3A knockdown reduced Treg cell activation and decreased FOXP3 and CD25 expression (Fig. 5R, S3G-H). These findings demonstrated that STT3A knockdown inhibited Wnt signaling and Treg cell activation in HCC by suppressing N-glycosylation of FCN3.

### STT3A accelerated HCC progression by promoting Treg cell activation in vivo

**Fig. 6:**
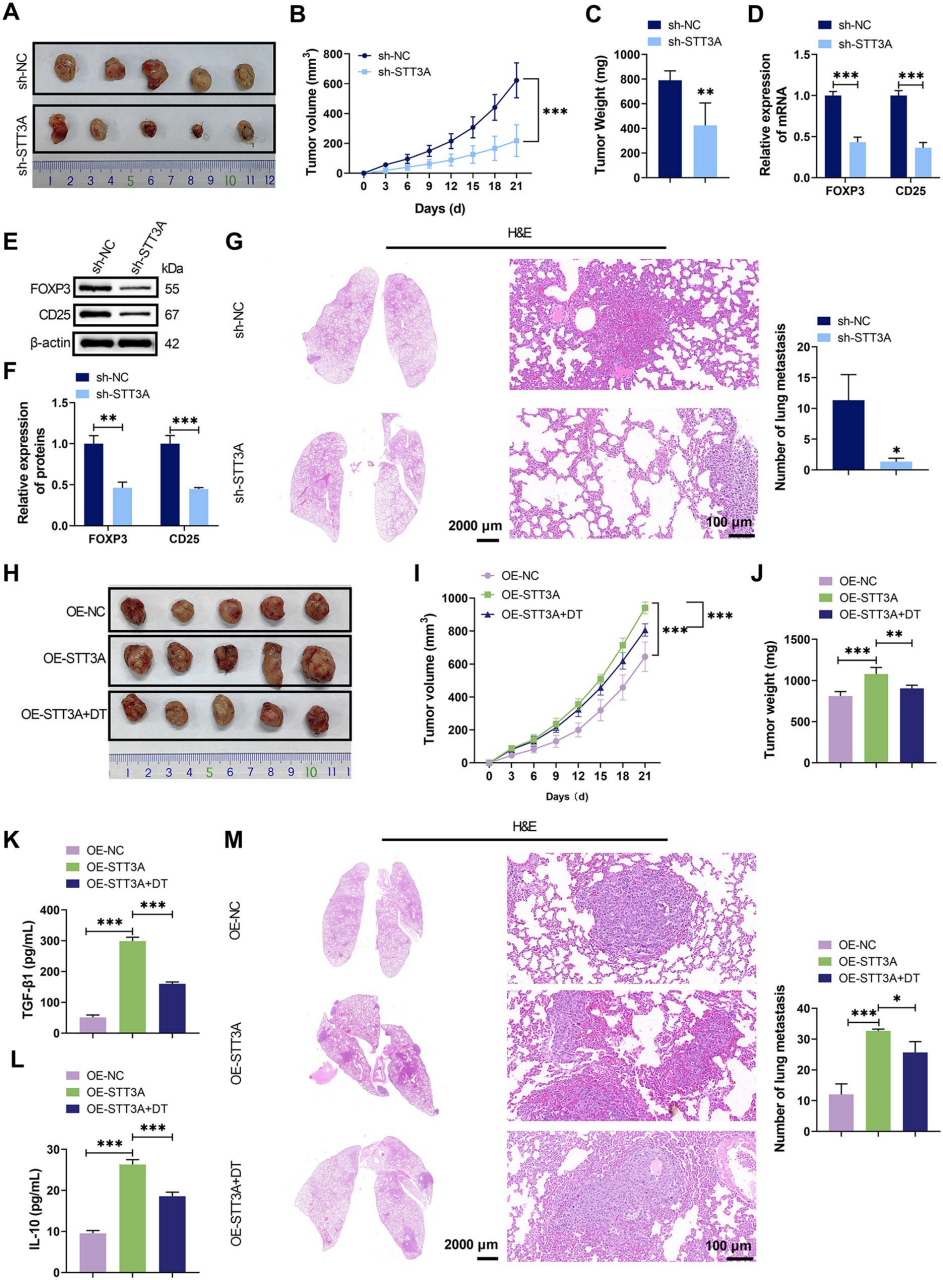
STT3A promoted HCC development by regulating Treg cell activation in vivo. (**A**) Representative tumors of C57BL/6 mice at day 21 post-inoculation of Hepa1-6 cells with sh-STT3A or sh-NC transfection. (**B**-**C**) Tumor volume and body weight recorded during 21 days post Hepa1-6 cell inoculation demonstrated the effect of sh-STT3A on HCC tumor growth. (**D**-**F**) qRT-PCR and WB analysis of FOXP3 and CD25 in tumor tissues from HCC mice with sh-STT3A or sh-NC. (**G**) H&E-stained lung sections showing the impact of sh-STT3A on the number of metastatic nodules in HCC mice. (**H**-**J**) Tumor growth evaluation (images, volume, weight) in mice receiving Hepa1-6 cells with OE-NC, OE-STT3A, or OE-STT3A + DT. (**K**-**L**) ELISA assessing the influence of OE-STT3A and OE-STT3A + DT on TGF-β1 and IL-10 in HCC mouse tumor tissues. (**M**) Assessment of lung metastasis in HCC mice with OE-NC, OE-STT3A, or OE-STT3A + DT treatment using H&E staining. ^***^p < 0.001, ^**^p < 0.01, ^*^p < 0.05 vs. sh-NC/ OE-NC/ OE-STT3A

Subcutaneous xenograft models in male C57BL/6 mice were established by injecting Hepa1-6 cells transfected with sh-NC or sh-STT3A constructs, with knockdown efficiency verified in Figure S4A-B. STT3A knockdown significantly reduced tumor volume and weight (Fig. 6A-C) and decreased FOXP3 and CD25 expression in tumor tissues (Fig. 6D-F). Analysis of lung tissue sections from HCC mice showed fewer metastatic nodules in the sh-STT3A group compared to sh-NC group (Fig. 6G). To examine the role of activated Treg cells in STT3A-driven HCC progression, mice injected with STT3A-overexpressing Hepa1-6 cells (overexpression efficiency verified in Figure S4C-D) were administered DT to deplete Treg cells. STT3A overexpression promoted tumor growth, increased TGF-β1 and IL-10 levels, and enhanced pulmonary metastasis, all of which were significantly reversed by DT treatment (Fig. 6H-M). These results demonstrated that STT3A exacerbated HCC progression by promoting Treg cell activation.

## Discussion

Utilizing HCC clinical samples, animal models and cell lines, this study innovatively elucidated the mechanism by which STT3A-mediated N-glycosylation of FCN3 promoted HCC progression through Wnt/β-catenin-dependent Treg cell activation. These findings significantly advanced our understanding of HCC immunosuppression and provided novel potential therapeutic targets for overcoming immunotherapy resistance.

The identification of FCN3 as a suppressor of Treg activation in this study offered crucial insights into HCC immunobiology. While previous studies have established the roles of FCN3 in complement activation and iron metabolism in HCC [14, 16], this work was the first to demonstrate that FCN3 inhibited HCC progression by regulating Treg cell activation. Notably, the suppressive effects of FCN3 overexpression on both Treg activation and HCC cell growth were reversed by the β-catenin agonist LY2090314, indicating that FCN3 reduced Treg activation by inhibiting the Wnt/β-catenin pathway. This finding aligns with reports showing that blocking Wnt/β-catenin signaling can inhibit tumor progression in triple-negative breast cancer, colorectal cancer, gastric cancer and clear cell renal cell carcinoma by suppressing Treg cell activation and infiltration [12, 29–31]. The present research was the first to identify FCN3 as an endogenous blocker of the Wnt/β-catenin pathway in HCC that inhibited Treg activation and reduced immunosuppressive cytokines (TGF-β1 and IL-10), thereby expanding current knowledge. While previous studies have reported that FCN3 can interact with proteins such as pro-IR and influence their processing [16], our Co-IP assay revealed no direct physical interaction between FCN3 and APC or β-catenin in normal hepatocytes, suggesting that FCN3 may regulate APC expression indirectly, possibly at the transcriptional level. Although the precise intermediary mechanisms remain to be fully elucidated, this finding underscores the complexity of FCN3-mediated regulation within the Wnt signaling cascade. Furthermore, these results in the present study also provided a mechanistic explanation for the previously established but poorly understood association between Wnt/β-catenin activation and immunosuppression in HCC [32].

The glycosylation-dependent regulation of FCN3 function represented another major finding of this study. It was demonstrated that N-glycosylation at Asn189 significantly altered the ability of FCN3 to modulate Wnt/β-catenin signaling, Treg activation and immunosuppressive factors. This discovery further substantiated the critical role of protein glycosylation in cancer immunity [33, 34]. Particularly noteworthy was the observation that N-glycosylation disrupted the tumor-suppressive function of FCN3, suggesting that HCC cells might actively exploit this post-translational modification to create an immunosuppressive microenvironment. Our data further revealed that N‑glycosylation at Asn189 promoted the secretion of FCN3 into the extracellular space, whereas its inhibition via the N189Q mutation enhanced intracellular accumulation of FCN3. This observation aligns with previous reports indicating that glycosylation facilitates protein secretion [35, 36]. Furthermore, prior studies have confirmed that secreted or exogenous FCN3 exerts limited effects on cancer cell phenotypes [37], while FCN3 primarily promotes HCC cell death through its intracellular functions [16]. Therefore, N‑glycosylation of FCN3 may not only impair its function but also reduce its intracellular retention, thereby attenuating its tumor‑suppressive activity. As an N-glycosyltransferase, STT3A has been shown to drive tumor immune evasion and immunosuppression in HCC through mediating PD-L1 glycosylation [38, 39]. This investigation firstly proposed and validated STT3A as the key glycosyltransferase mediating FCN3 glycosylation in HCC. Moreover, STT3A promoted Wnt pathway activation and Treg cell activation in HCC through facilitating N-glycosylation of FCN3. These findings provided mechanistic support for the previously reported correlation between STT3A levels and Treg infiltration in HCC [27].

In HCC animal models, STT3A knockdown not only significantly inhibited tumor growth and metastasis but also suppressed β-catenin activation and Treg cell activation. This dual effect offered new perspectives for overcoming current limitations in HCC immunotherapy. Recent studies have shown that small-molecule inhibitors targeting STT3A can effectively inhibit tumor cell N-glycosylation and enhance NK cell-mediated antitumor activity [40, 41], thus providing direct translational support for our findings. In colorectal cancer, the β-catenin inhibitor iCRT14 has demonstrated synergistic antitumor effects when combined with Treg depletion therapy [42]. This suggests that STT3A inhibitors could be combined with existing immunotherapies to enhance treatment efficacy by simultaneously blocking tumor-intrinsic glycosylation modifications and alleviating Treg-mediated immunosuppression. Given the limited response rates to current immune checkpoint inhibitors in HCC [43], therapeutic strategies targeting the STT3A-FCN3-β-catenin axis may provide new treatment options for advanced HCC patients.

Several important questions warrant further investigation. First, the precise structural consequences of FCN3 glycosylation, particularly how Asn189 modification affects its interaction with APC, require further investigation. Second, potential crosstalk between FCN3 glycosylation and other post-translational modifications including phosphorylation and ubiquitination in regulating Wnt signaling deserves exploration. Third, although this work focused on Tregs, the impact of the STT3A-FCN3-β-catenin axis on other immune cell populations such as CD8^+^ T cells and NK cells should be examined. In conclusion, this study identified STT3A-mediated FCN3 glycosylation as a crucial mechanism driving HCC immunosuppression through Wnt/β-catenin-dependent Treg activation. These findings not only deepened our understanding of HCC immune evasion but also suggested novel therapeutic intervention strategies. The STT3A-FCN3-β-catenin axis represented a promising target for combination therapies in HCC.

## Conclusion

Our findings established STT3A-mediated FCN3 glycosylation as a novel mechanism driving HCC immunosuppression through β-catenin-dependent Treg activation. This work revealed the dual role of FCN3 as both a tumor suppressor and immunomodulator, whose function was modulated by N-glycosylation at Asn189. The STT3A-FCN3 axis represented a promising therapeutic target to simultaneously inhibit oncogenic Wnt/β-catenin signaling and enhance anti-tumor immunity. Future studies should explore clinical translation of STT3A-targeting agents and their integration with existing immunotherapies in HCC.

## Supplementary Information

Below is the link to the electronic supplementary material.

Supplementary Material 1

Supplementary Material 2

## Data Availability

No datasets were generated or analysed during the current study.

## Abbreviations

Asn189: Asparagine 189
CCK-8: Cell counting kit-8
Co-IP: Co-immunoprecipitation
DT: Diphtheria toxin
ELISA: Enzyme-linked immunosorbent assay
FCN3: Ficolin-3
FCN3-N189Q: FCN3 N189Q mutant
FCN3-WT: Wild-type FCN3
H&E: Hematoxylin and eosin
HCC: Hepatocellular carcinoma
IF: Immunofluorescence
IHC: Immunohistochemistry
OE-NC: Empty vector control
OE-FCN3: FCN3 overexpression
OST: Oligosaccharyltransferase
PBMCs: Peripheral blood mononuclear cells
p-β-catenin: phosphorylated β-catenin
qRT-PCR: Quantitative real-time fluorescence PCR
sh-FCN3: FCN3 knockdown
sh-NC: Non-targeting control
sh-STT3A: STT3A knockdown
STT3A: OST complex catalytic subunit A
TM: Tunicamycin
TME: Tumor microenvironment
Tregs: Regulatory T cells
WB: Western blot

## References

[1] B. Alawyia, C. Constantinou, Current treatment options in oncology 24, 711–724 (2023) 10.1007/s11864-023-01098-9 10.1007/s11864-023-01098-9 37103744

[2] B. Foglia, C. Turato, S. Cannito, Int. J. Mol. Sci. **24** (2023). 10.3390/ijms241512224 10.3390/ijms241512224 PMC10419038 37569600

[3] C. Chen, Z. Wang, Y. Ding, Y. Qin, Front. Immunol. **14** (2023). 113330810.3389/fimmu.2023.1133308 10.3389/fimmu.2023.1133308 PMC9950271 36845131

[4] Y. Liu, Z. Xun, K. Ma, S. Liang, X. Li, S. Zhou, L. Sun, Y. Liu, Y. Du, X. Guo, T. Cui, H. Zhou, J. Wang, D. Yin, R. Song, S. Zhang, W. Cai, F. Meng, H. Guo, B. Zhang, D. Yang, R. Bao, Q. Hu, J. Wang, Y. Ye, L. Liu, J. Hepatol. **78**, 770–782 (2023). 10.1016/j.jhep.2023.01.011 36708811 10.1016/j.jhep.2023.01.011

[5] J.H. Kang, R. Zappasodi, Trends Cancer. **9**, 911–927 (2023). 10.1016/j.trecan.2023.07.015 37598003 10.1016/j.trecan.2023.07.015 PMC13390109

[6] F. Shan, A. Somasundaram, T.C. Bruno, C.J. Workman, D.A.A. Vignali, Trends Cancer. **8**, 944–961 (2022). 10.1016/j.trecan.2022.06.008 35853825 10.1016/j.trecan.2022.06.008 PMC9588644

[7] X. Luo, W. Huang, S. Li, M. Sun, D. Hu, J. Jiang, Z. Zhang, Y. Wang, Y. Wang, J. Zhang, Z. Wu, X. Ji, D. Liu, X. Chen, B. Zhang, H. Liang, Y. Li, B. Liu, S. Wang, X. Xu, Y. Nie, K. Wu, D. Fan, L. Xia, Advanced science (Weinheim, Baden-Wurttemberg, Germany) **11**, e2310304 (2024) 10.1002/advs.202310304 10.1002/advs.202310304 PMC11423149 39072947

[8] H. Liu, Cellular and molecular gastroenterology and hepatology 19, 101478 (2025) 10.1016/j.jcmgh.2025.101478 10.1016/j.jcmgh.2025.101478 PMC12009096 39999950

[9] C. Xu, Z. Xu, Y. Zhang, M. Evert, D.F. Calvisi, X. Chen, J. Clin. Investig. **132** (2022). 10.1172/jci154515 10.1172/JCI154515 PMC8843739 35166233

[10] Y. Zhu, Y. He, R. Gan, Cells. **13** (2024). 10.3390/cells13231990 10.3390/cells13231990 PMC11640042 39682738

[11] M. Feng, J.Q. Jin, L. Xia, T. Xiao, S. Mei, X. Wang, X. Huang, J. Chen, M. Liu, C. Chen, S. Rafi, A.X. Zhu, Y.X. Feng, D. Zhu, Sci. Adv. **5**, eaau5240 (2019). 10.1126/sciadv.aau5240 31086813 10.1126/sciadv.aau5240 PMC6506245

[12] L. Ji, W. Qian, L. Gui, Z. Ji, P. Yin, G.N. Lin, Y. Wang, B. Ma, W.Q. Gao, Cancer Res. **80**, 2004–2016 (2020). 10.1158/0008-5472.Can-19-3074 32156780 10.1158/0008-5472.CAN-19-3074

[13] L. Sun, S. Yu, C. Dong, Z. Wu, H. Huang, Z. Chen, Z. Wu, X. Yin, Front. Genet. **13**, 913398 (2022). 10.3389/fgene.2022.913398 35928441 10.3389/fgene.2022.913398 PMC9343789

[14] G. Zheng, L. Wu, H. Bouamar, M. Cserhati, Y.C. Chiu, C.S. Hinck, Ł. Wieteska, C.R. Zeballos Torrez, R. Hu, A. Easley, Y. Chen, A.P. Hinck, F.G. Cigarroa, L.Z. Sun, Life Sci. **357**, 123103 (2024). 10.1016/j.lfs.2024.123103 39357793 10.1016/j.lfs.2024.123103 PMC13173836

[15] D. Ma, P. Liu, J. Wen, Y. Gu, Z. Yang, J. Lan, H. Fan, Z. Liu, D. Guo, Int. J. Biol. Sci. **19**, 362–376 (2023). 10.7150/ijbs.69784 36632465 10.7150/ijbs.69784 PMC9830510

[16] Y. Yuan, J. Xu, Q. Jiang, C. Yang, N. Wang, X. Liu, H.L. Piao, S. Lu, X. Zhang, L. Han, Z. Liu, J. Cai, F. Liu, S. Chen, J. Liu, J. Experimental Clin. Cancer Research: CR. **43**, 133 (2024). 10.1186/s13046-024-03047-2 10.1186/s13046-024-03047-2 PMC11067213 38698462

[17] P.M. Couto, C.M.A. Guardia, F.L. Couto, C.A. Labriola, M.S. Labanda, J.J. Caramelo, FASEB Journal: Official Publication Federation Am. Soc. Experimental Biology. **38**, e23782 (2024). 10.1096/fj.202302267R 10.1096/fj.202302267R PMC11307252 38934375

[18] Y. Lin, D.M. Lubman, Acta Pharm. Sinica B **14**, 1098–1110 (2024). 10.1016/j.apsb.2023.10.014 10.1016/j.apsb.2023.10.014 PMC10935144 38486989

[19] M. Hu, R. Zhang, J. Yang, C. Zhao, W. Liu, Y. Huang, H. Lyu, S. Xiao, D. Guo, C. Zhou, J. Tang, Cell death & disease **14**, 222 (2023) 10.1038/s41419-023-05733-z 10.1038/s41419-023-05733-z PMC10060418 36990999

[20] A. DelaCourt, A. Black, P. Angel, R. Drake, Y. Hoshida, A. Singal, D. Lewin, B. Taouli, S. Lewis, M. Schwarz, M.I. Fiel, A.S. Mehta, Mol. Cancer Research: MCR. **19**, 1868–1877 (2021). 10.1158/1541-7786.Mcr-21-0348 34380744 10.1158/1541-7786.MCR-21-0348 PMC8802325

[21] Nucleic acids research 51, D523-d531, (2023) 10.1093/nar/gkac1052 10.1093/nar/gkac1052 PMC9825514 36408920

[22] M. Ma, R. Dubey, A. Jen, G.V. Pusapati, B. Singal, E. Shishkova, K.A. Overmyer, V. Cormier-Daire, J. Fedry, L. Aravind, J.J. Coon, R. Rohatgi, Science (New York, N.Y.) **386**, 667–672 (2024) 10.1126/science.adp7201 10.1126/science.adp7201 PMC7617332 39509507

[23] H. Lu, C.S. Fermaintt, N.A. Cherepanova, R. Gilmore, N. Yan, M.A. Lehrman, Proc. Natl. Acad. Sci. U.S.A. **115**, 9557–9562 (2018). 10.1073/pnas.1806034115 30181269 10.1073/pnas.1806034115 PMC6156661

[24] C. Shi, Y. Wang, M. Wu, Y. Chen, F. Liu, Z. Shen, Y. Wang, S. Xie, Y. Shen, L. Sang, Z. Zhang, Z. Gao, L. Yang, L. Qu, Z. Yang, X. He, Y. Guo, C. Pan, J. Che, H. Ju, J. Liu, Z. Cai, Q. Yan, L. Yu, L. Wang, X. Dong, P. Xu, J. Shao, Y. Liu, X. Li, W. Wang, R. Zhou, T. Zhou, A. Lin, Nat. Commun. **13**, 6951 (2022). 10.1038/s41467-022-34346-x 36376293 10.1038/s41467-022-34346-x PMC9663433

[25] J. Wang, H.M. Zhang, G.H. Zhu, L.L. Zhao, J. Shi, Z.T. Dai, J.P. Li, X.R. Li, F. Sun, Y. Wu, S.Y. Chen, H.N. Li, X.H. Liao, Y. Xiang, Acta Pharmacol. Sin. **46**, 1097–1110 (2025). 10.1038/s41401-024-01419-0 39668180 10.1038/s41401-024-01419-0 PMC11950364

[26] D. Wang, S. Wu, J. He, L. Sun, H. Zhu, Y. Zhang, S. Liu, X. Duan, Y. Wang, T. Xu, J. Experimental Clin. Cancer Research: CR. **42**, 222 (2023). 10.1186/s13046-023-02758-2 10.1186/s13046-023-02758-2 PMC10472690 37658376

[27] S. Lin, Y. Cao, K. Zhu, C. Yang, X. Zhu, H. Zhang, R. Zhang, J. Hepatocellular Carcinoma. **10**, 1749–1765 (2023). 10.2147/jhc.S417407 37841372 10.2147/JHC.S417407 PMC10575065

[28] J. Zhang, E. Wang, Q. Li, Y. Peng, H. Jin, S. Naseem, B. Sun, S. Park, S. Choi, X. Li, Int. J. Biol. Macromol. **275**, 133639 (2024). 10.1016/j.ijbiomac.2024.133639 38969042 10.1016/j.ijbiomac.2024.133639

[29] X. Wang, M. Feng, T. Xiao, B. Guo, D. Liu, C. Liu, J. Pei, Q. Liu, Y. Xiao, R. Rosin-Arbesfeld, Y. Shi, Y. Zhou, M. Yang, Y.X. Feng, Y. Jiang, Z. Shao, K. Yu, D. Zhu, Oncogene. **40**, 2982–2997 (2021). 10.1038/s41388-021-01756-y 33767438 10.1038/s41388-021-01756-y

[30] T. Deng, Y. Hou, G. Lin, C. Feng, K. Liu, W. Chen, W. Wei, L. Huang, X. Dai, Pharmaceutics 15, (2023) 10.3390/pharmaceutics15030944 10.3390/pharmaceutics15030944 PMC10053243 36986805

[31] Z. Che, W. Jin, Y. Wu, H. Li, P. Liang, Sci. Rep. **14**, 27053 (2024). 10.1038/s41598-024-78713-8 39511359 10.1038/s41598-024-78713-8 PMC11543667

[32] W. Xu, C. Nie, H. Lv, B. Chen, J. Wang, S. Wang, J. Zhao, Y. He, X. Chen, Front. Immunol. **13**, 1010554 (2022). 10.3389/fimmu.2022.1010554 36275697 10.3389/fimmu.2022.1010554 PMC9582750

[33] J.G. Rodrigues, H.O. Duarte, C.A. Reis, J. Gomes, Biochem. Soc. Trans. **49**, 843–854 (2021). 10.1042/bst20200763 33704376 10.1042/BST20200763

[34] H. Läubli, L. Borsig, Front. Immunol. **10**, 2120 (2019). 10.3389/fimmu.2019.02120 31552050 10.3389/fimmu.2019.02120 PMC6743365

[35] J. Benicky, M. Sanda, Z. Brnakova Kennedy, R. Goldman, J. Biol. Chem. **294**, 16816–16830 (2019). 10.1074/jbc.RA119.009989 31558607 10.1074/jbc.RA119.009989 PMC6851326

[36] Y. Li, D. Shi, F. Yang, X. Chen, Y. Xing, Z. Liang, J. Zhuang, W. Liu, Y. Gong, J. Jiang, Y. Wei, FEBS Lett. **593**, 719–731 (2019). 10.1002/1873-3468.13358 30873590 10.1002/1873-3468.13358

[37] H. Jang, Y. Jun, S. Kim, E. Kim, Y. Jung, B.J. Park, J. Lee, J. Kim, S. Lee, J. Kim, Cell Death Dis. **12**, 407 (2021). 10.1038/s41419-021-03675-y 33859174 10.1038/s41419-021-03675-y PMC8050313

[38] X. Guo, T. Cui, L. Sun, Y. Fu, C. Cheng, C. Wu, Y. Zhu, S. Liang, Y. Liu, S. Zhou, X. Li, C. Ji, K. Ma, N. Zhang, Q. Chu, C. Xing, S. Deng, J. Wang, Y. Liu, L. Liu, Cell Death Differ. **32**, 944–958 (2025). 10.1038/s41418-024-01432-0 39690246 10.1038/s41418-024-01432-0 PMC12089503

[39] H.X. Shi, C. Liang, C.Y. Yao, Z.X. Gao, J. Qin, J.L. Cao, M.Z. Zhang, Y.Y. Li, M.Q. Wang, H. Sun, S.Q. Xie, D. Fang, Cell. Communication Signaling: CCS. **20**, 175 (2022). 10.1186/s12964-022-00981-6 36348350 10.1186/s12964-022-00981-6 PMC9644467

[40] D. Zhang, J. Xie, F. Sun, R. Xu, W. Liu, J. Xu, X. Huang, G. Zhang, Cancer Lett. **589**, 216819 (2024). 10.1016/j.canlet.2024.216819 38522775 10.1016/j.canlet.2024.216819

[41] M. Baro, H. Lee, V. Kelley, R. Lou, C. Phoomak, K. Politi, C.J. Zeiss, M. Van Zandt, J.N. Contessa, Cell. Chem. Biology. **32**, 839–853e836 (2025). 10.1016/j.chembiol.2025.05.005 10.1016/j.chembiol.2025.05.005 PMC13107401 40494352

[42] C. Wang, J. Yan, P. Yin, L. Gui, L. Ji, B. Ma, W.Q. Gao, Oncoimmunology. **9**, 1809947 (2020). 10.1080/2162402x.2020.1809947 32939327 10.1080/2162402X.2020.1809947 PMC7470182

[43] F. Bicer, C. Kure, A.A. Ozluk, B.F. El-Rayes, M. Akce, Curr. Oncol. (Toronto Ont). **30**, 9789–9812 (2023). 10.3390/curroncol30110711 10.3390/curroncol30110711 PMC10670350 37999131

