# Supplementary material for: STT3A-mediated FCN3 N-glycosylation promotes Treg cell activation to drive hepatocellular carcinoma progression via Wnt/β-catenin
Source: Cell Oncol (Dordr). 2026 Jan 6;49(1):16. doi: 10.1007/s13402-025-01159-1 (PMC12774965; doi:10.1007/s13402-025-01159-1)

**Figure S1** Validation of FCN3 knockdown and overexpression efficiency. (A-B) qRT-PCR and WB analysis of FCN3 mRNA and protein levels in HCC-LM3 cells following FCN3 knockdown. (C-D) qRT-PCR and WB verification of FCN3 overexpression in Hep3B cells. ^***^*p* < 0.001, ^**^*p* < 0.01, ^*^*p* < 0.05 vs sh-NC/ OE-NC.

**Figure S2** FCN3 regulated Treg cell activation by inhibiting Wnt/β-catenin signaling. (A) Scatter plot of GO analysis showing the correlation between FCN3 and APC expression levels in normal liver tissues. (B) Correlation analysis of FCN3 and APC or β-catenin protein expression in clinical HCC tumor tissues. (C) Protein-protein interaction network was predicted by GeneMANIA (https://genemania.org), demonstrating potential functional associations between FCN3, APC, and Wnt signaling pathway components. (D) CO-IP assay in normal hepatocyte THLE‑2 showing no direct binding between FCN3 and APC or β‑catenin. (E) IF analysis of β-catenin expression and localization in normal hepatocyte THLE-2 and HCC cell lines (HepG2, Hep3B, HCC-LM3). (F-G) The effects of OE-FCN3 and OE-FCN3+LY2090314 treatment on β-catenin expression and cell viability of Hep3B cells were respectively evaluated by IF analysis and CCK-8 assay. (H‑I) qRT-PCR and WB analysis showing the impact of OE-FCN3 and OE-FCN3+LY2090314 treatment in Hep3B cells on the levels of Treg cell markers FOXP3 and CD25 in Hep3B-PBMC co-cultures. ^***^*p* < 0.001, ^**^*p* < 0.01, ^*^*p* < 0.05 vs OE-NC/ OE-FCN3+DMSO.

**Figure S3** STT3A knockdown suppressed HCC progression by regulating Wnt signaling and Treg cell activation. (A) Protein interaction network predicted by GeneMANIA (https://genemania.org) illustrating the complex regulatory relationships among STT3A, FCN3, APC, and β-catenin. (B-D) qRT-PCR and WB validation of STT3A knockdown efficiency in Hep3B cells. (E) The effect of sh-STT3A on β-catenin in Hep3B cells was assessed by IF analysis. (F) CCK-8 assay measuring cell viability of Hep3B cells after transfection with sh-STT3A or sh-NC. (G-H) Following sh-STT3A or sh-NC transfection, Treg cell markers FOXP3 and CD25 levels in Hep3B cells were quantified via qRT-PCR and WB analysis. ^***^*p* < 0.001, ^**^*p* < 0.01, ^*^*p* < 0.05 vs sh-NC.

**Figure S4** Validation of STT3A knockdown and overexpression efficiency in Hepa1-6 cells. (A-D) qRT-PCR and WB analysis of STT3A mRNA and protein levels in Hepa1-6 cells following sh-STT3A or OE-STT3A. ^***^*p* < 0.001, ^**^*p* < 0.01, ^*^*p* < 0.05 vs sh-NC/ OE-NC.

**Figure S1**


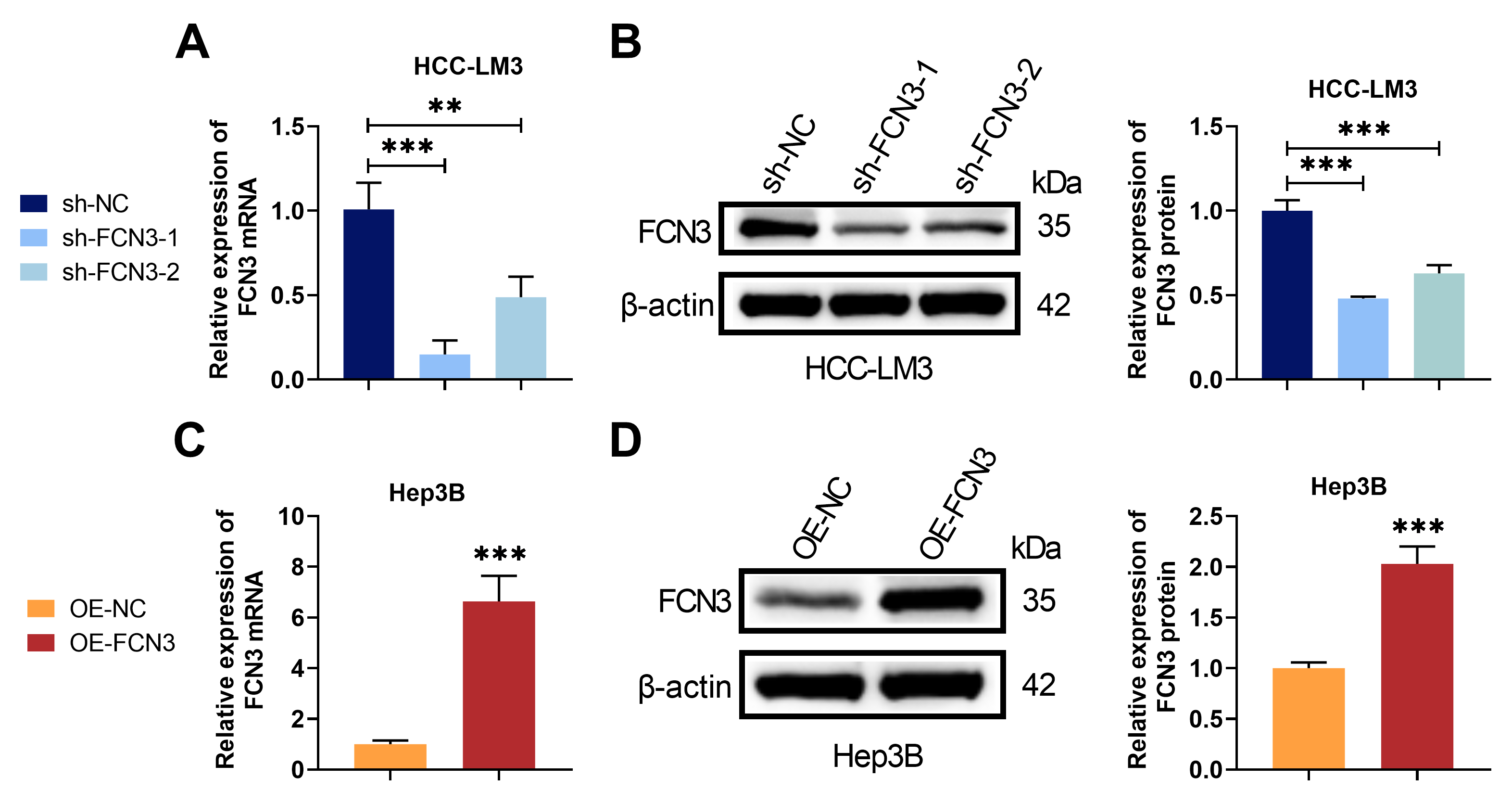


**Revised Figure S2**


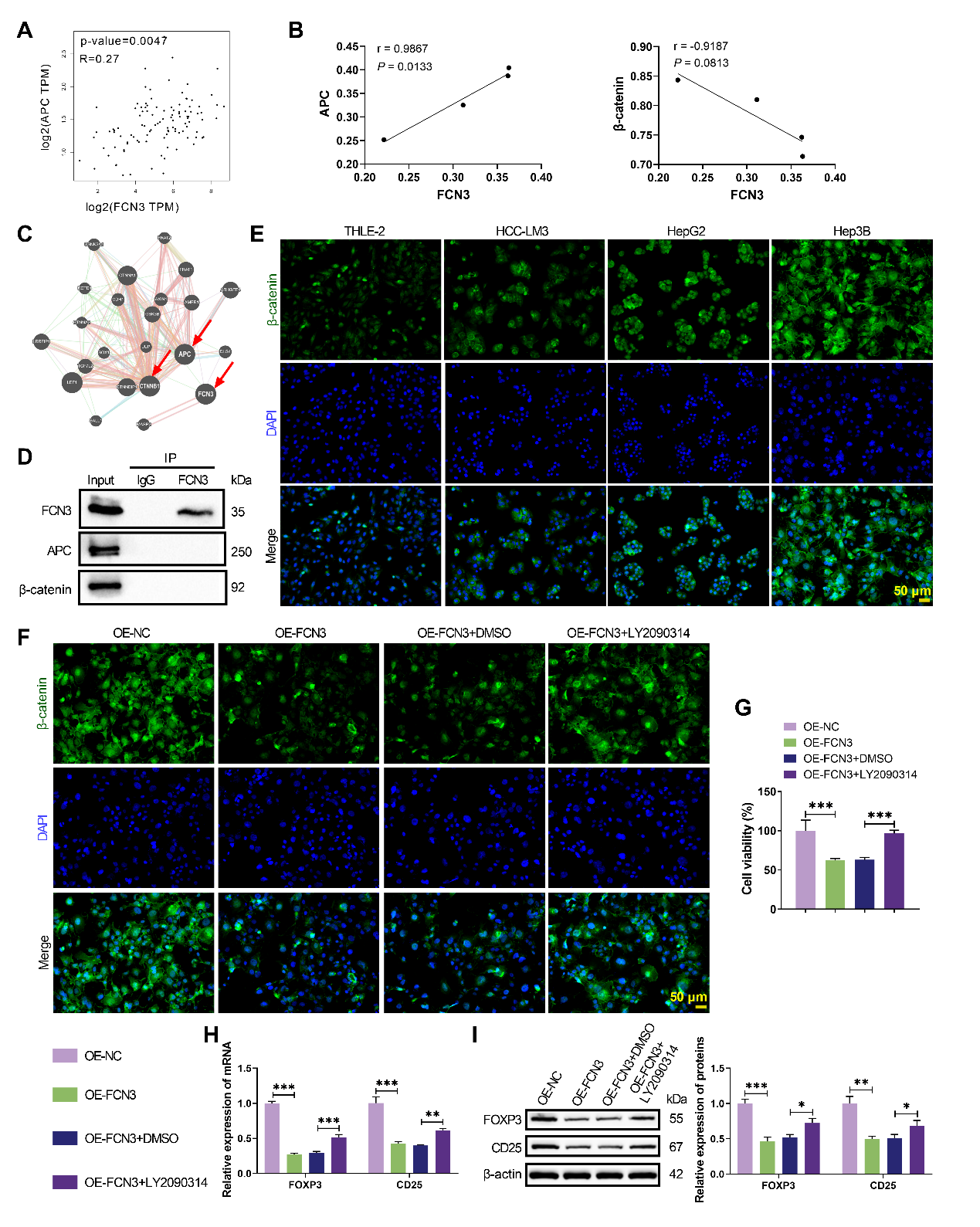


**Revised Figure S3**


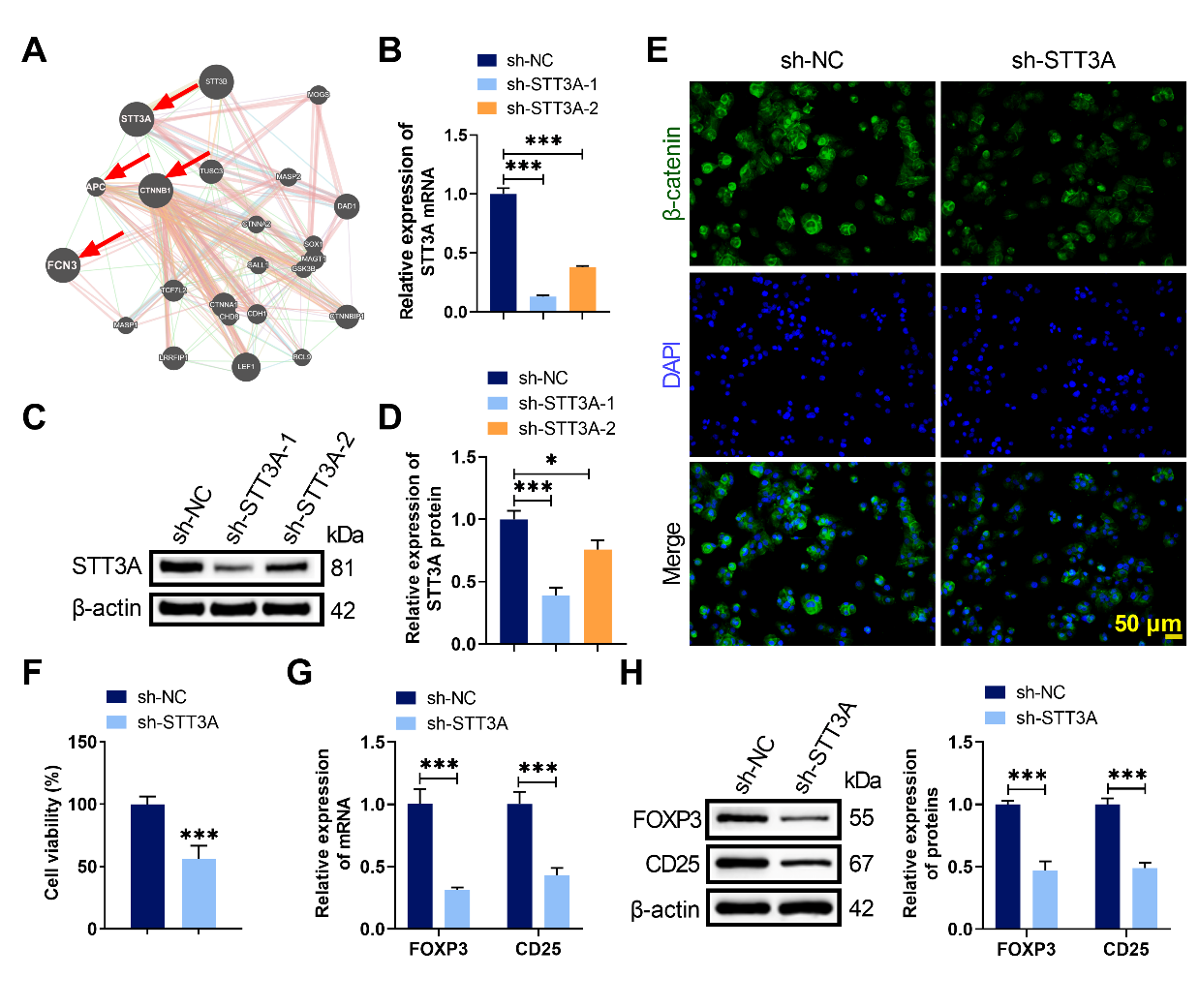


**Figure S4**


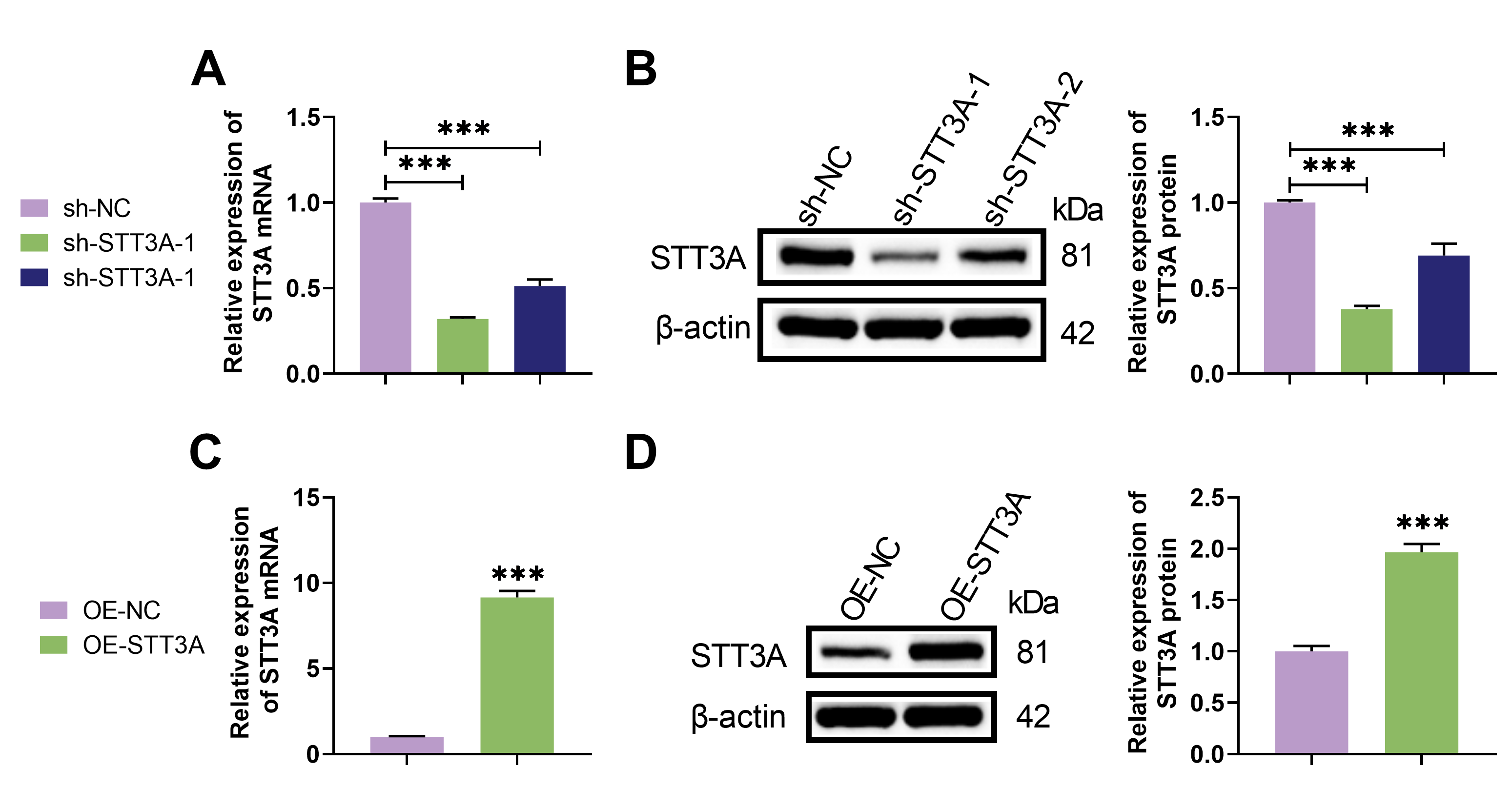

Supplement: Supplementary file 1 — Supplementary Material 1 [file 13402_2025_1159_MOESM1_ESM.docx]
