# Supplementary material for: STT3A-mediated FCN3 N-glycosylation promotes Treg cell activation to drive hepatocellular carcinoma progression via Wnt/β-catenin
Source: Cell Oncol (Dordr). 2026 Jan 6;49(1):16. doi: 10.1007/s13402-025-01159-1 (PMC12774965; doi:10.1007/s13402-025-01159-1)
Supplement: Supplementary file 2 — Supplementary Material 2 [file 13402_2025_1159_MOESM2_ESM.pptx]

## Slide 1
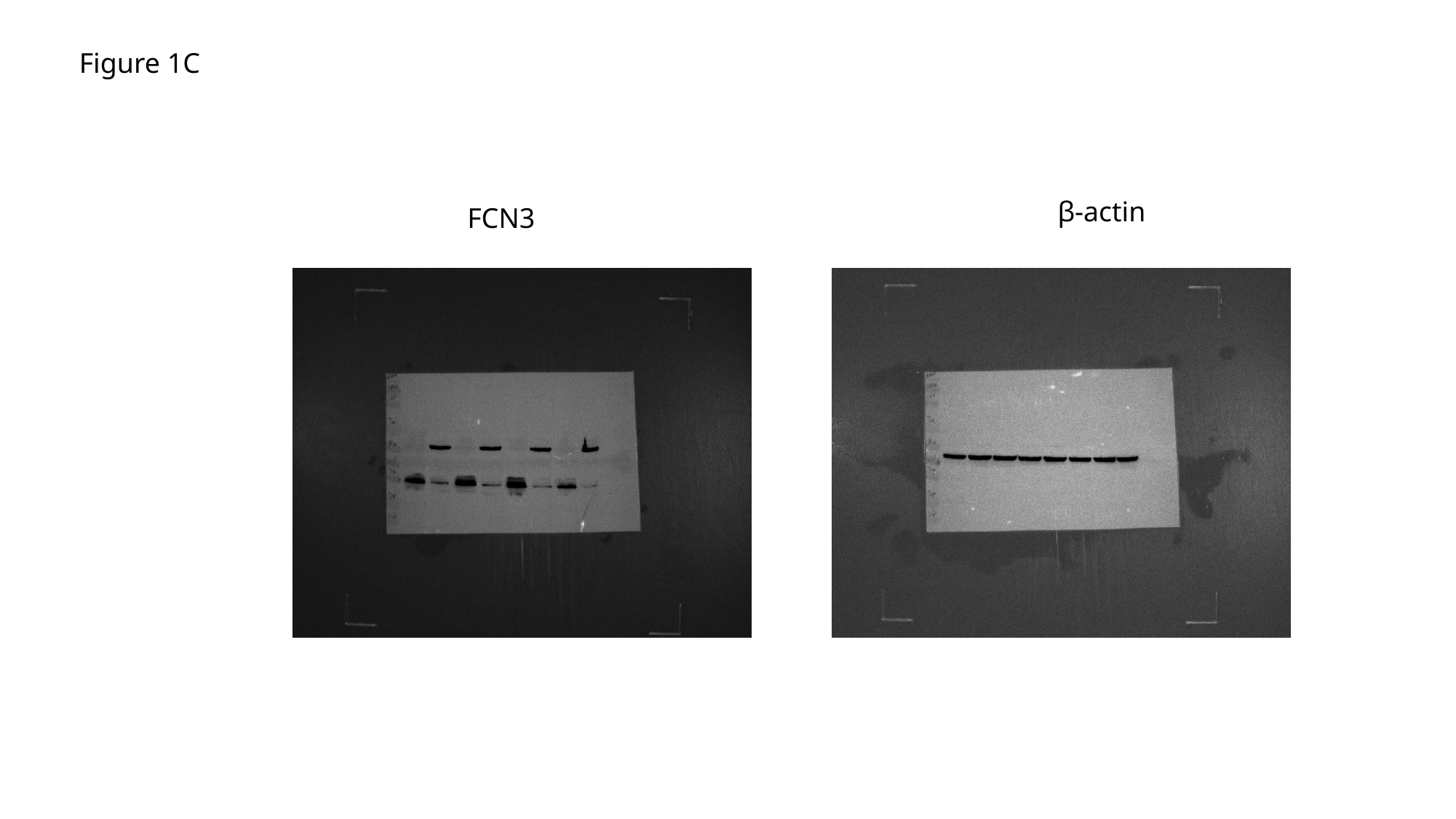

Figure 1C
β-actin
FCN3

## Slide 2
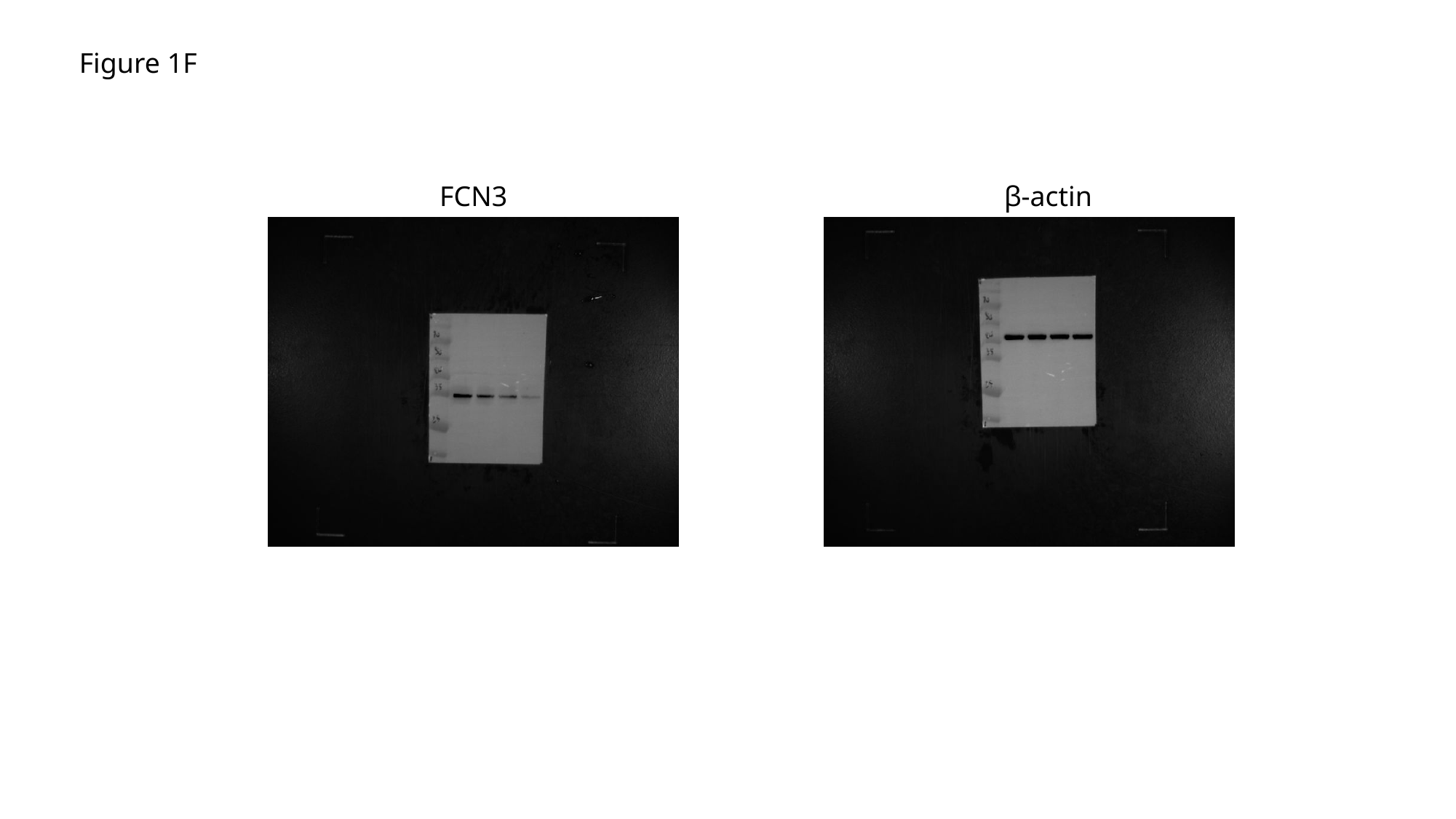

Figure 1F
FCN3
β-actin

## Slide 3
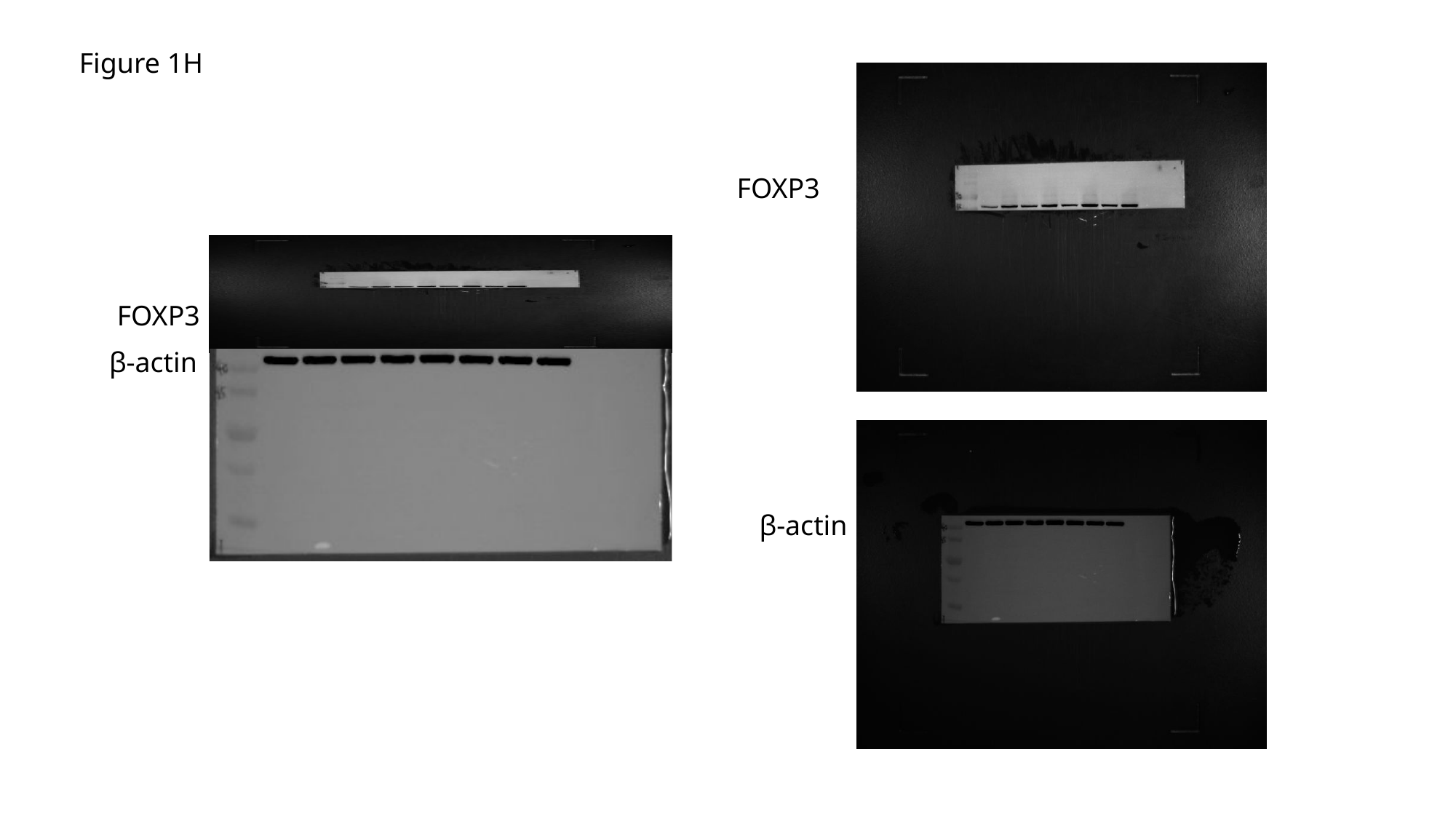

Figure 1H
FOXP3
FOXP3
β-actin
β-actin

## Slide 4
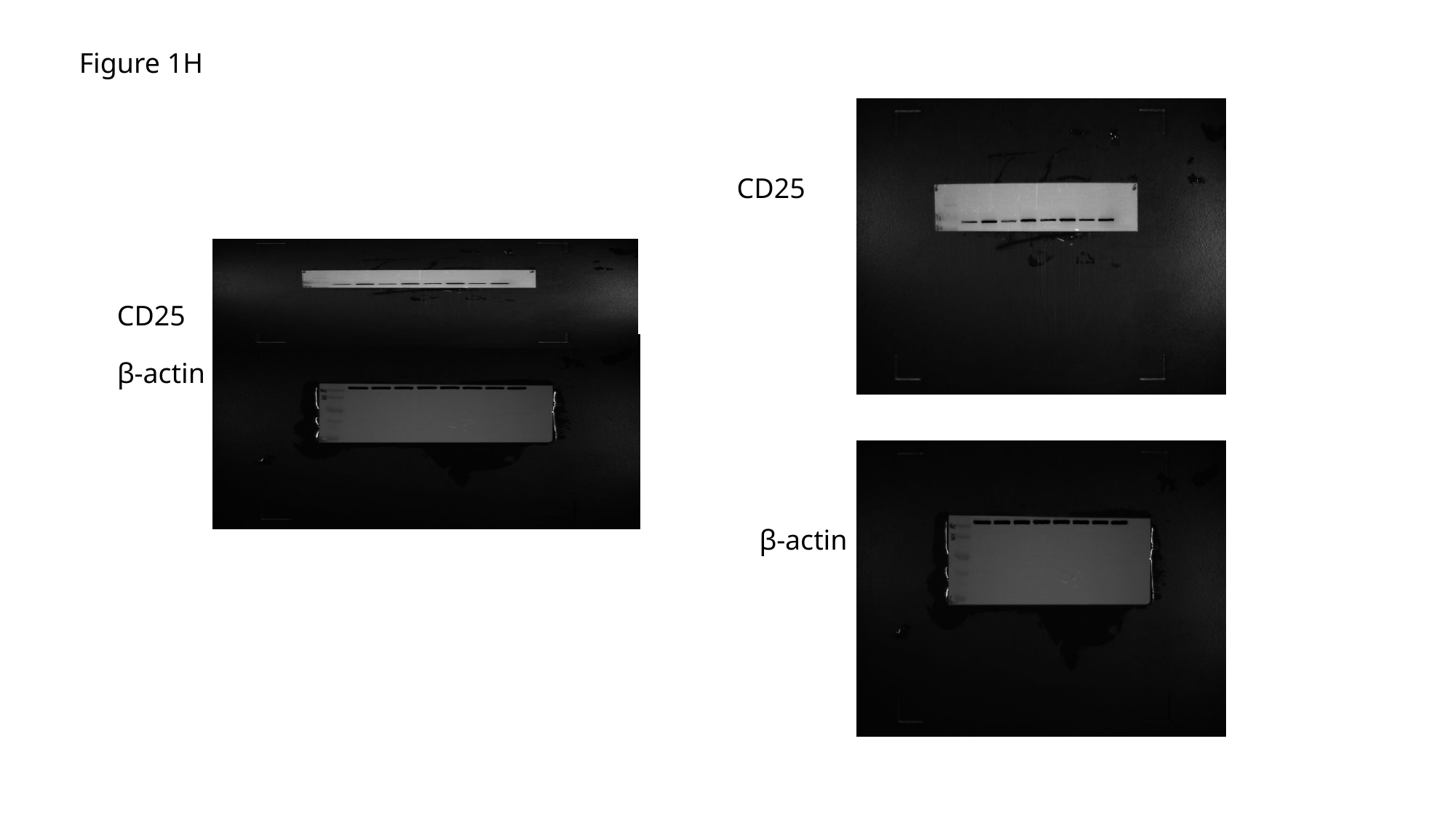

Figure 1H
CD25
CD25
β-actin
β-actin

## Slide 5
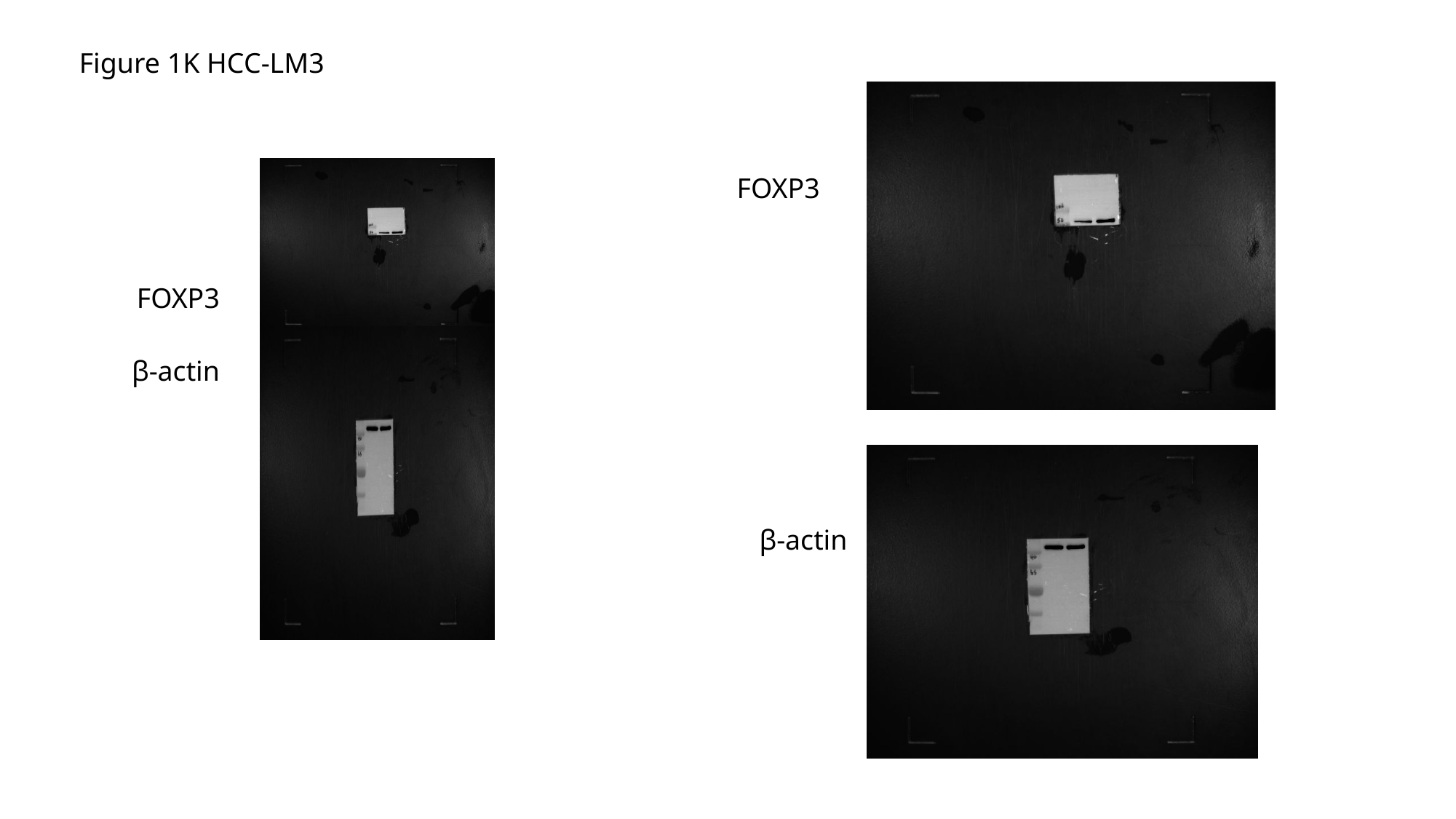

Figure 1K HCC-LM3
FOXP3
FOXP3
β-actin
β-actin

## Slide 6
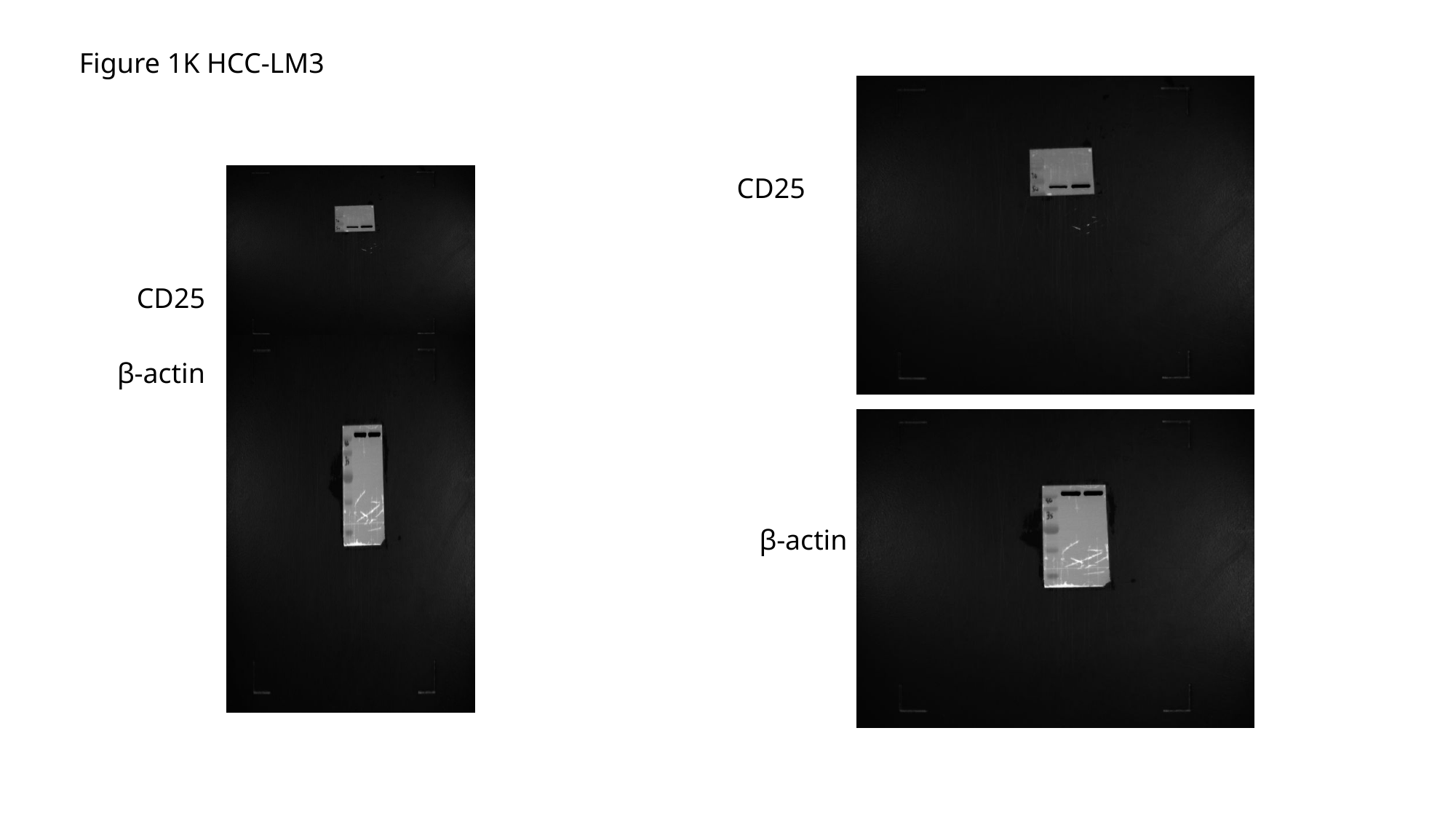

Figure 1K HCC-LM3
CD25
CD25
β-actin
β-actin

## Slide 7
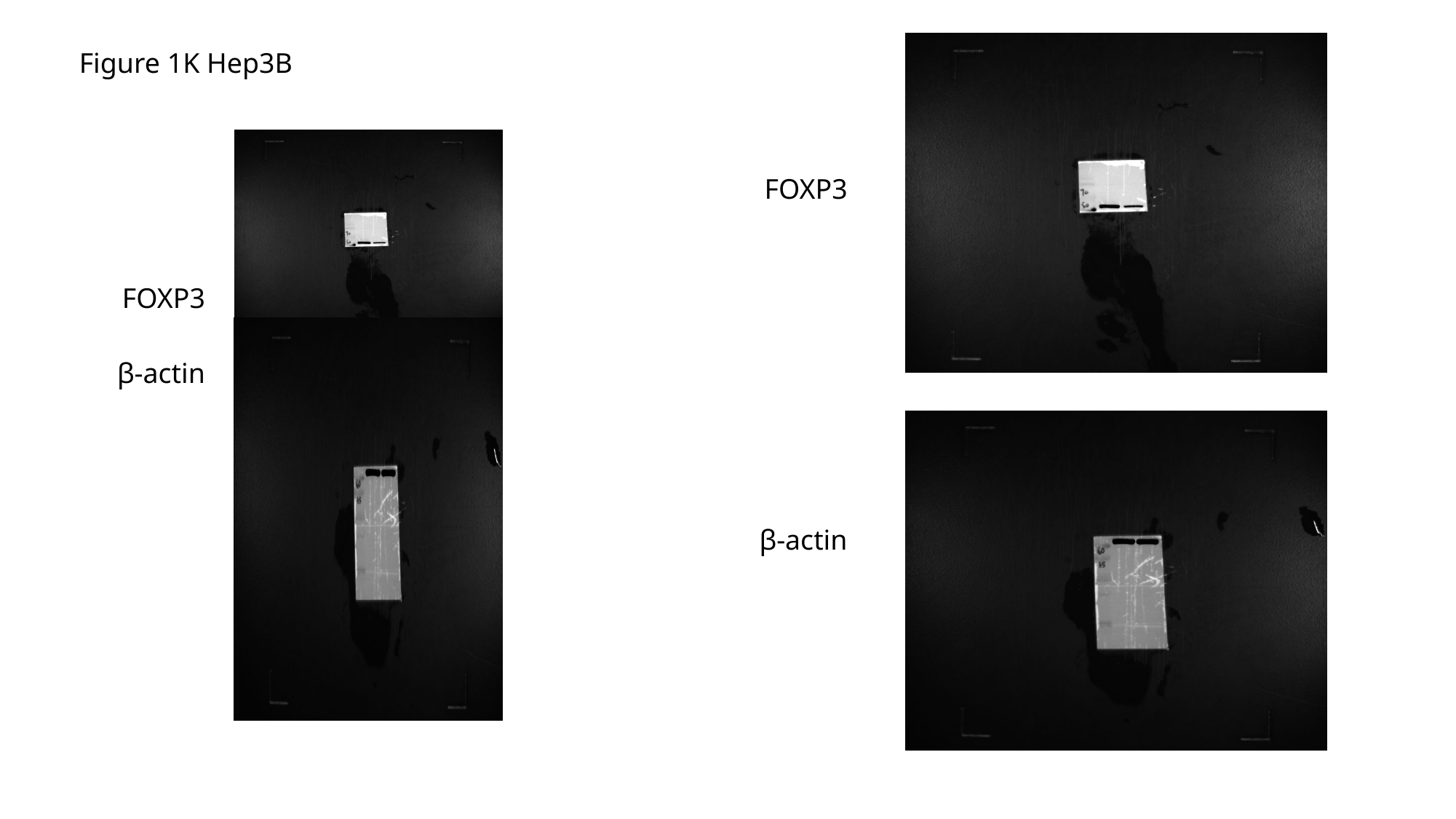

Figure 1K Hep3B
FOXP3
FOXP3
β-actin
β-actin

## Slide 8
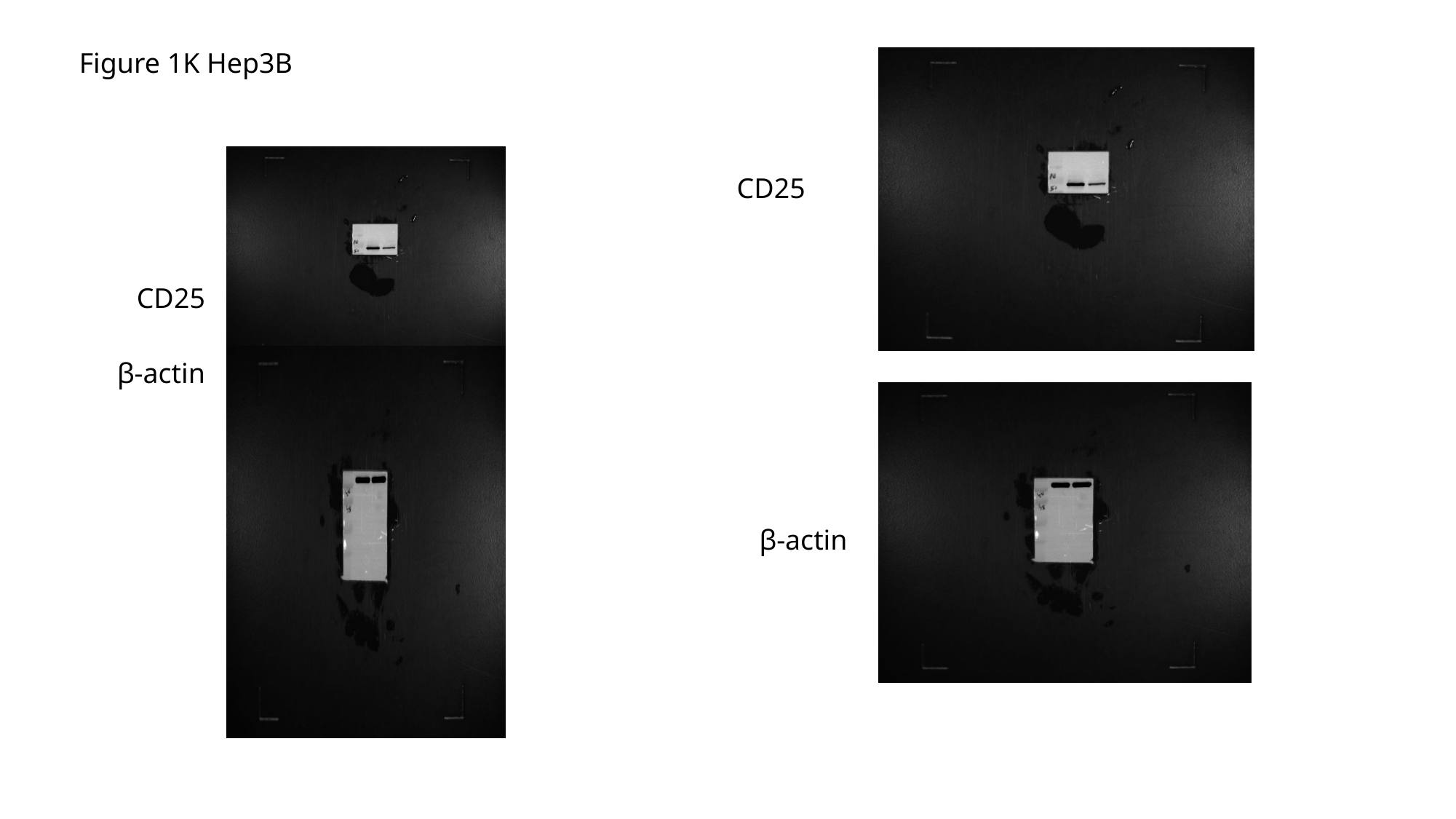

Figure 1K Hep3B
CD25
CD25
β-actin
β-actin

## Slide 9
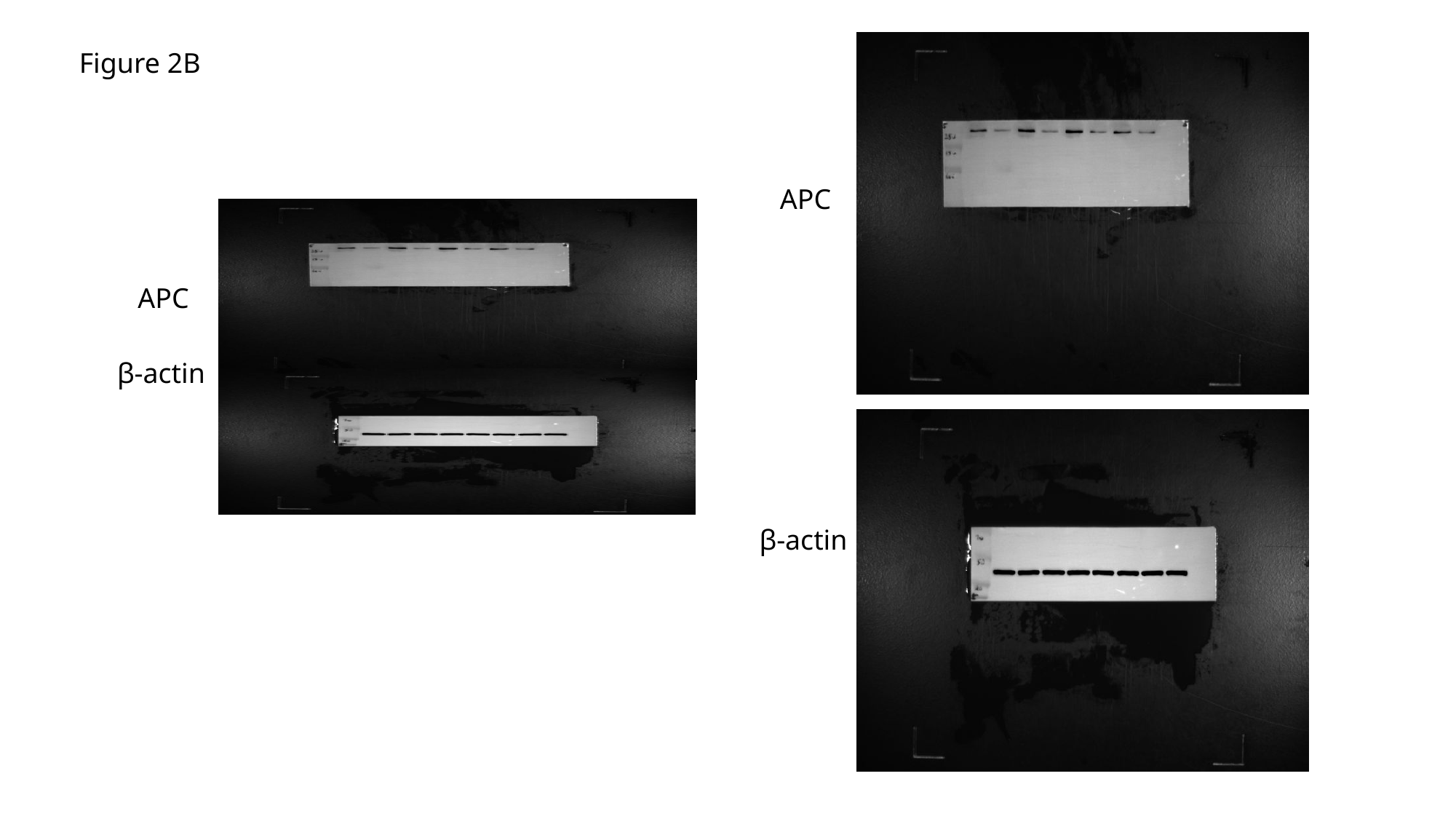

Figure 2B
APC
APC
β-actin
β-actin

## Slide 10
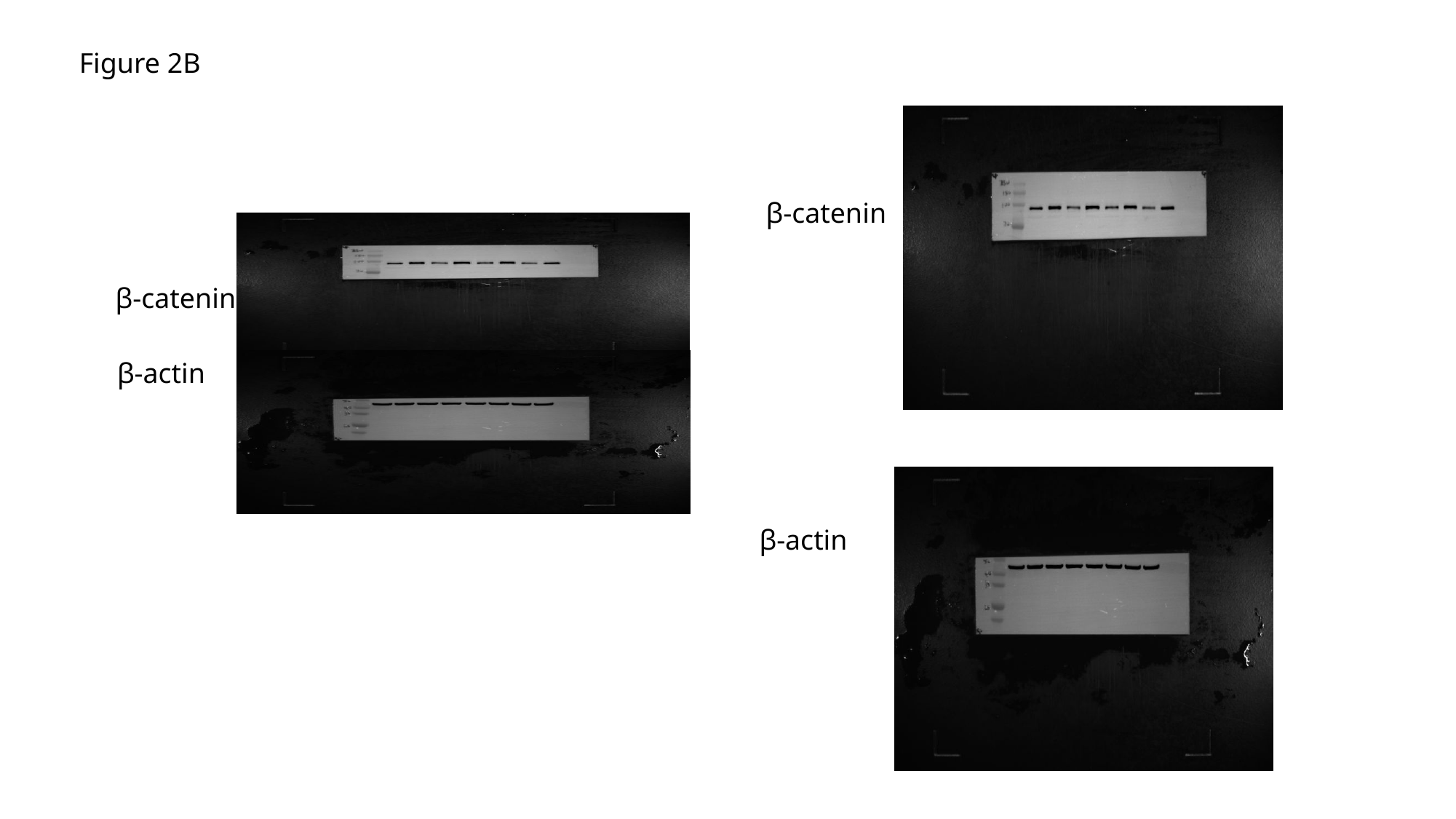

Figure 2B
β-catenin
β-catenin
β-actin
β-actin

## Slide 11
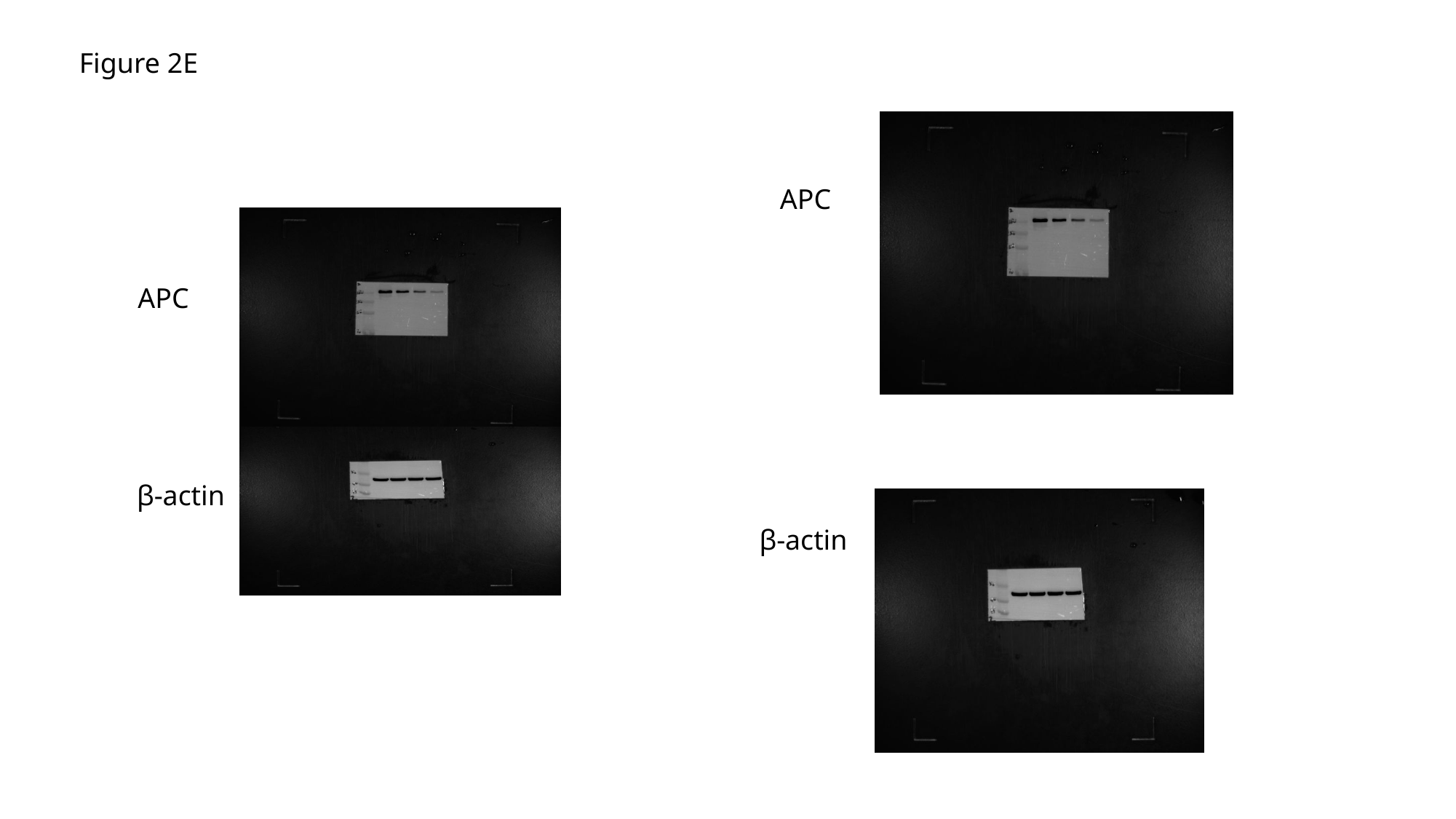

Figure 2E
APC
APC
β-actin
β-actin

## Slide 12
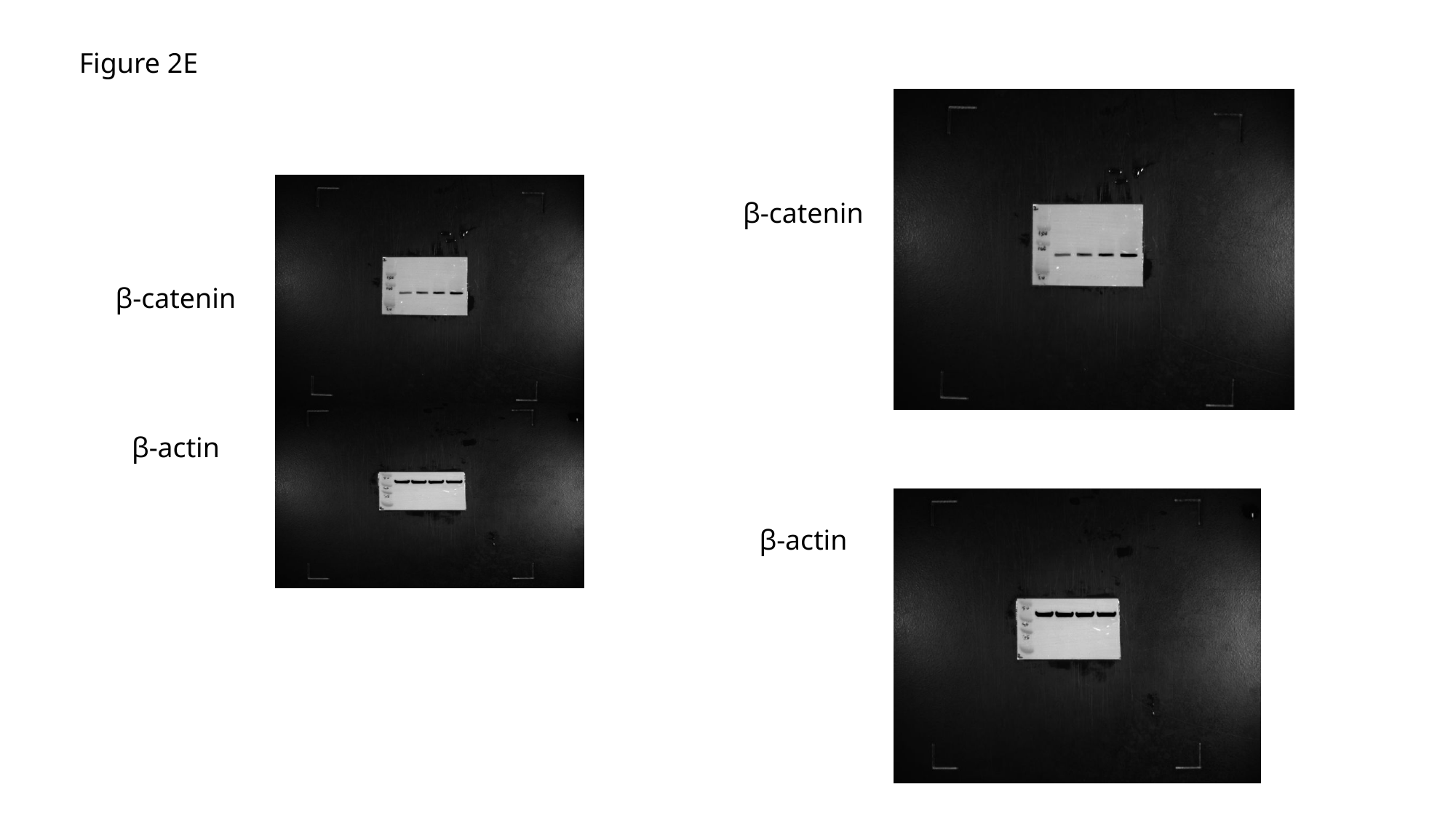

Figure 2E
β-catenin
β-catenin
β-actin
β-actin

## Slide 13
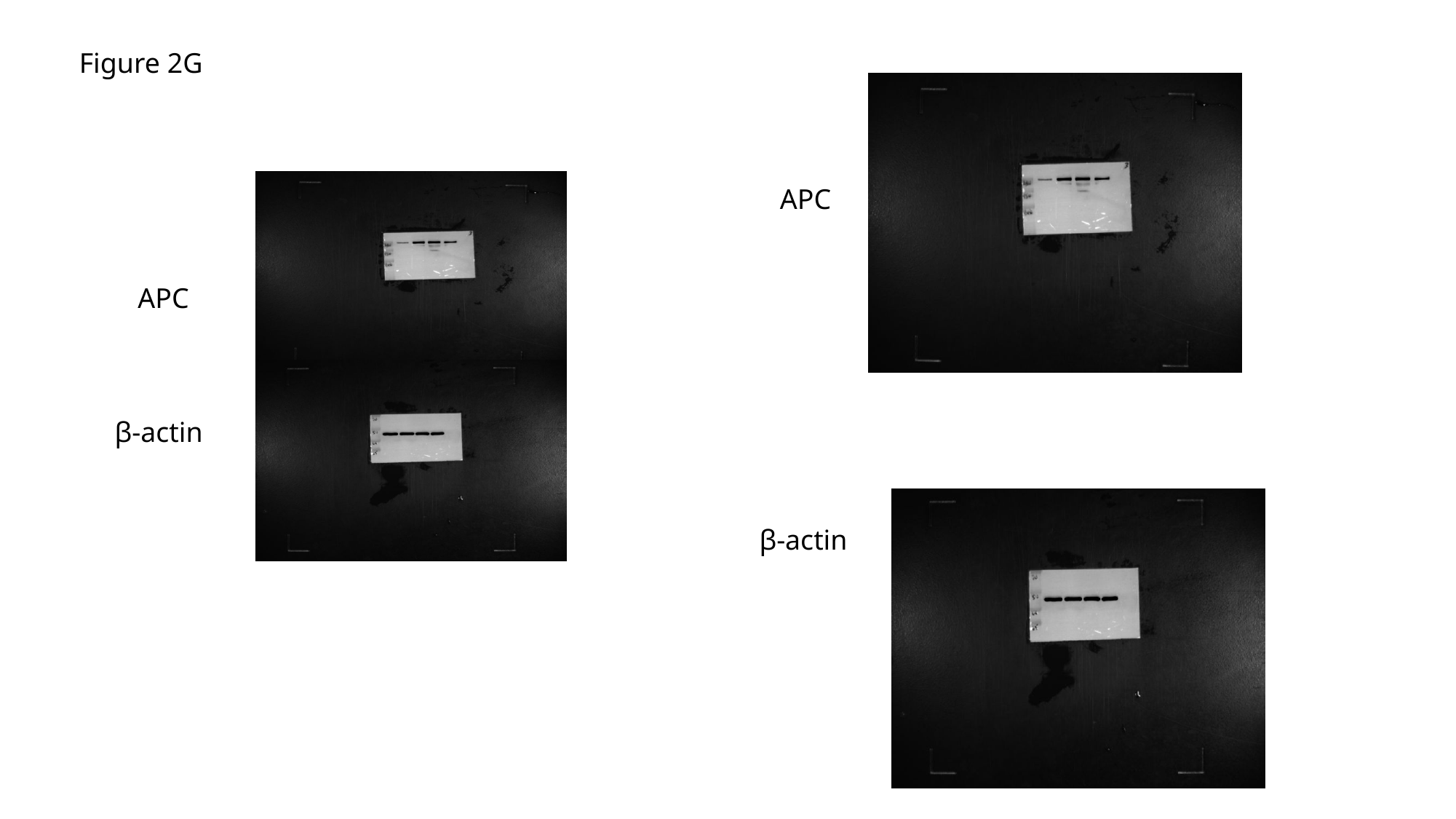

Figure 2G
APC
APC
β-actin
β-actin

## Slide 14
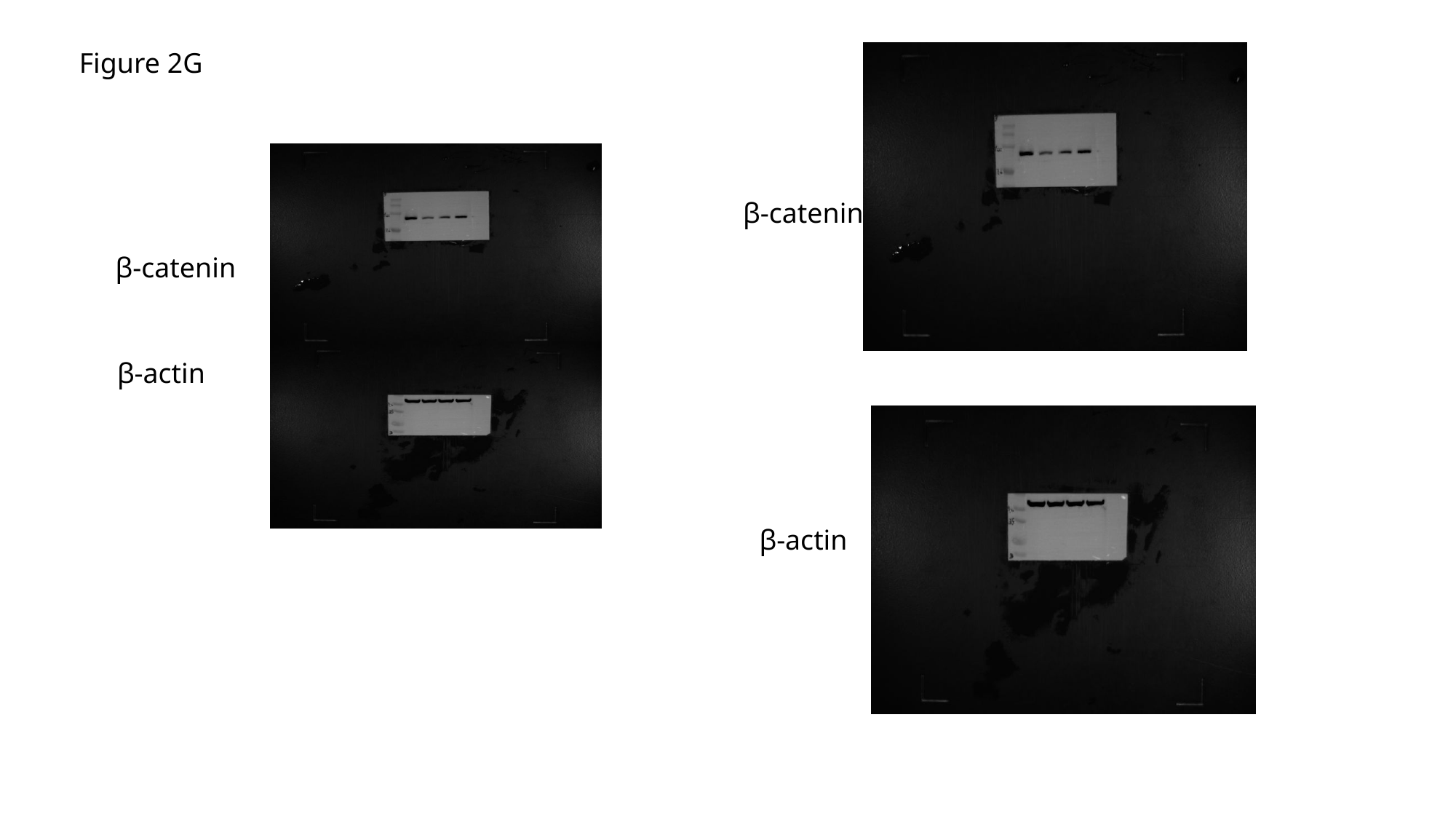

Figure 2G
β-catenin
β-catenin
β-actin
β-actin

## Slide 15
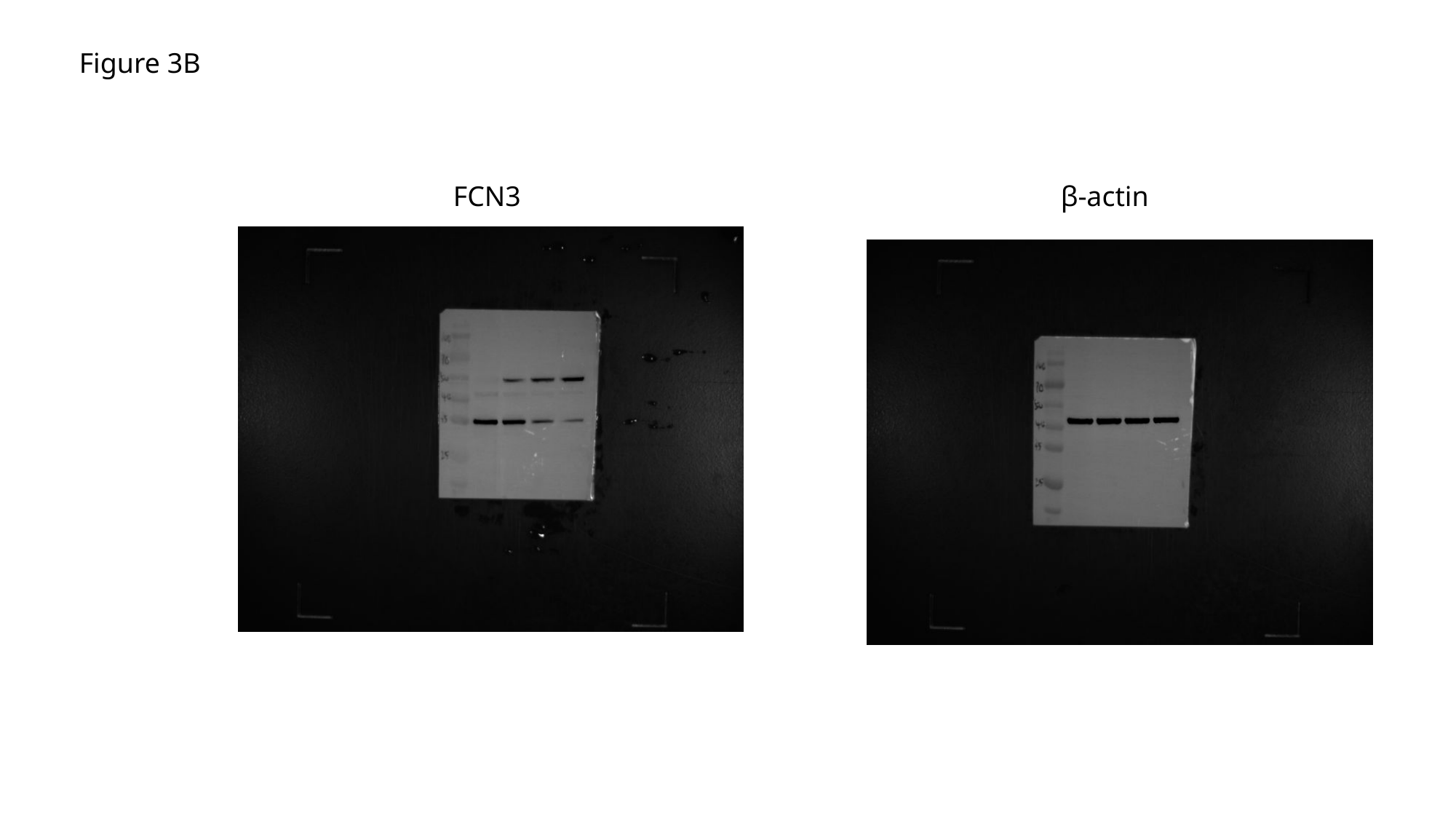

Figure 3B
FCN3
β-actin

## Slide 16
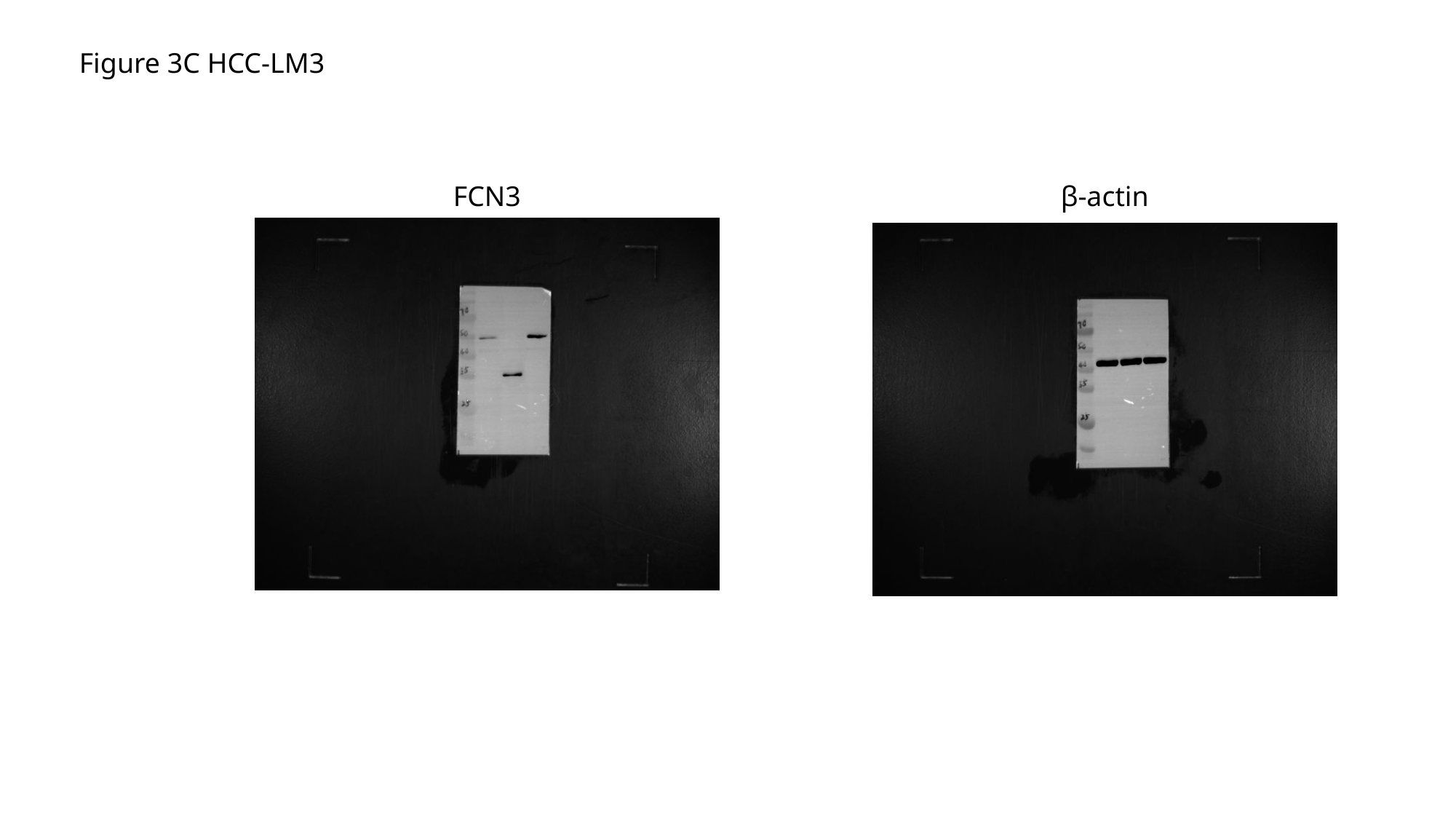

Figure 3C HCC-LM3
FCN3
β-actin

## Slide 17
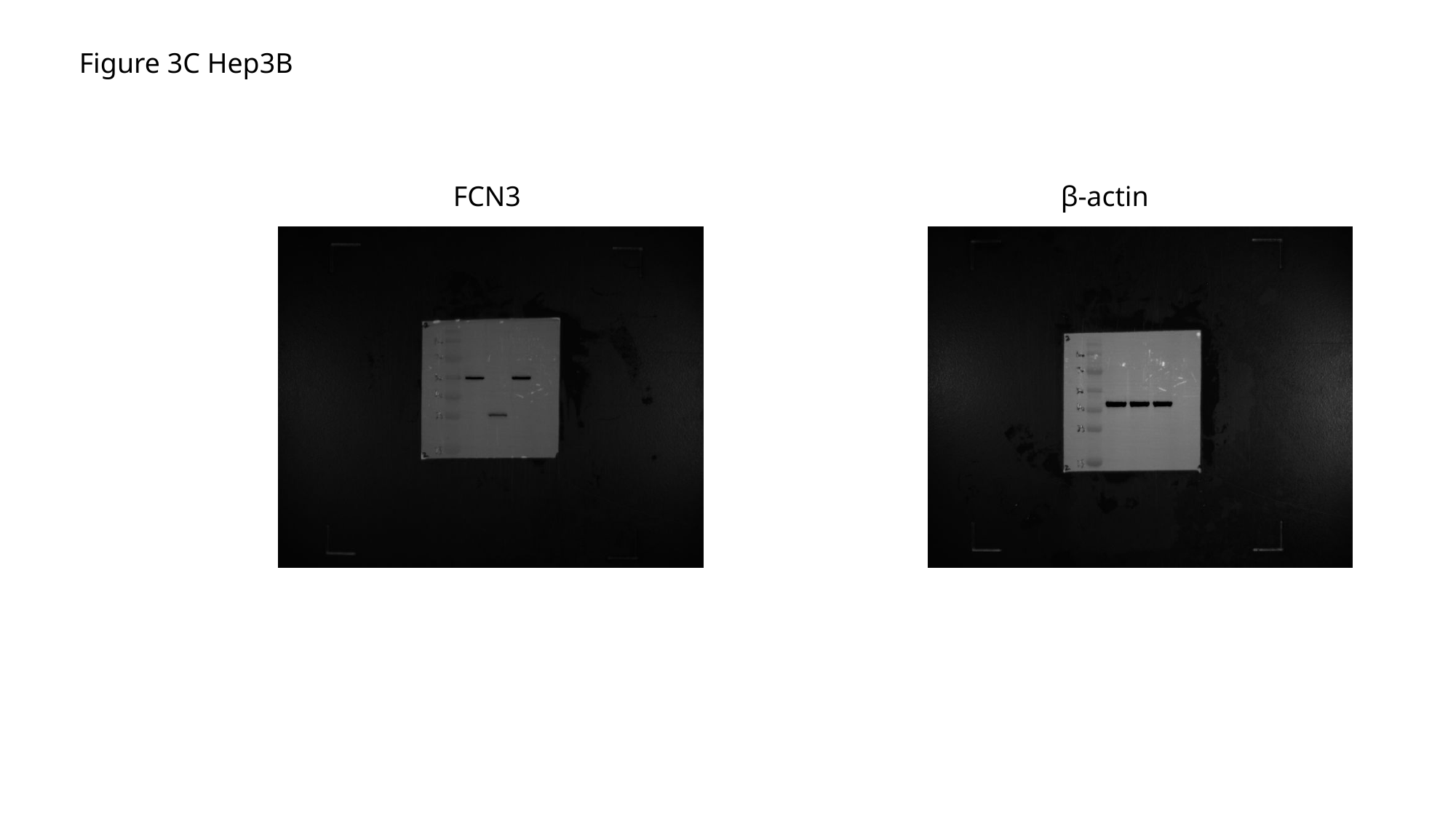

Figure 3C Hep3B
FCN3
β-actin

## Slide 18
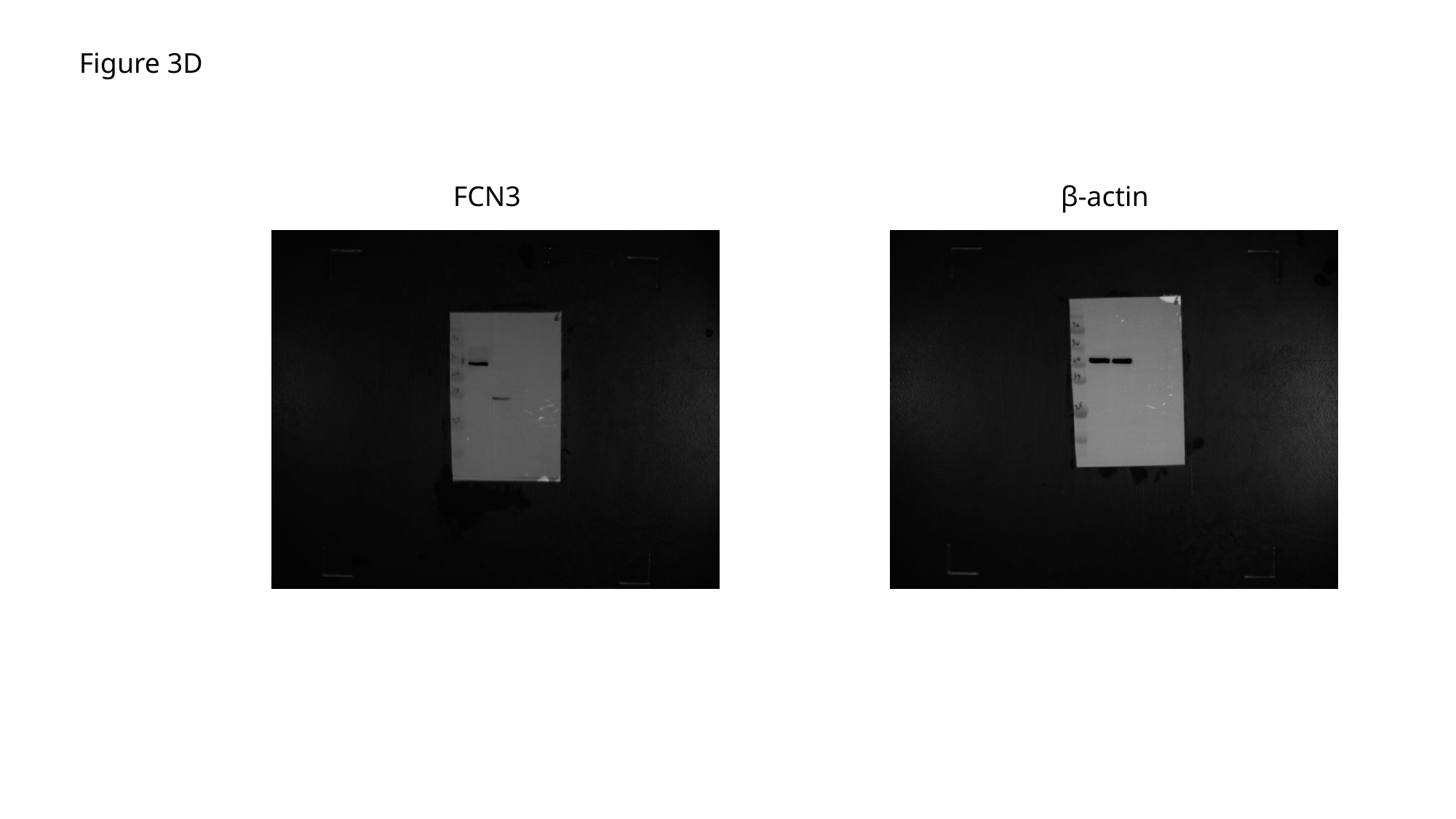

Figure 3D
FCN3
β-actin

## Slide 19
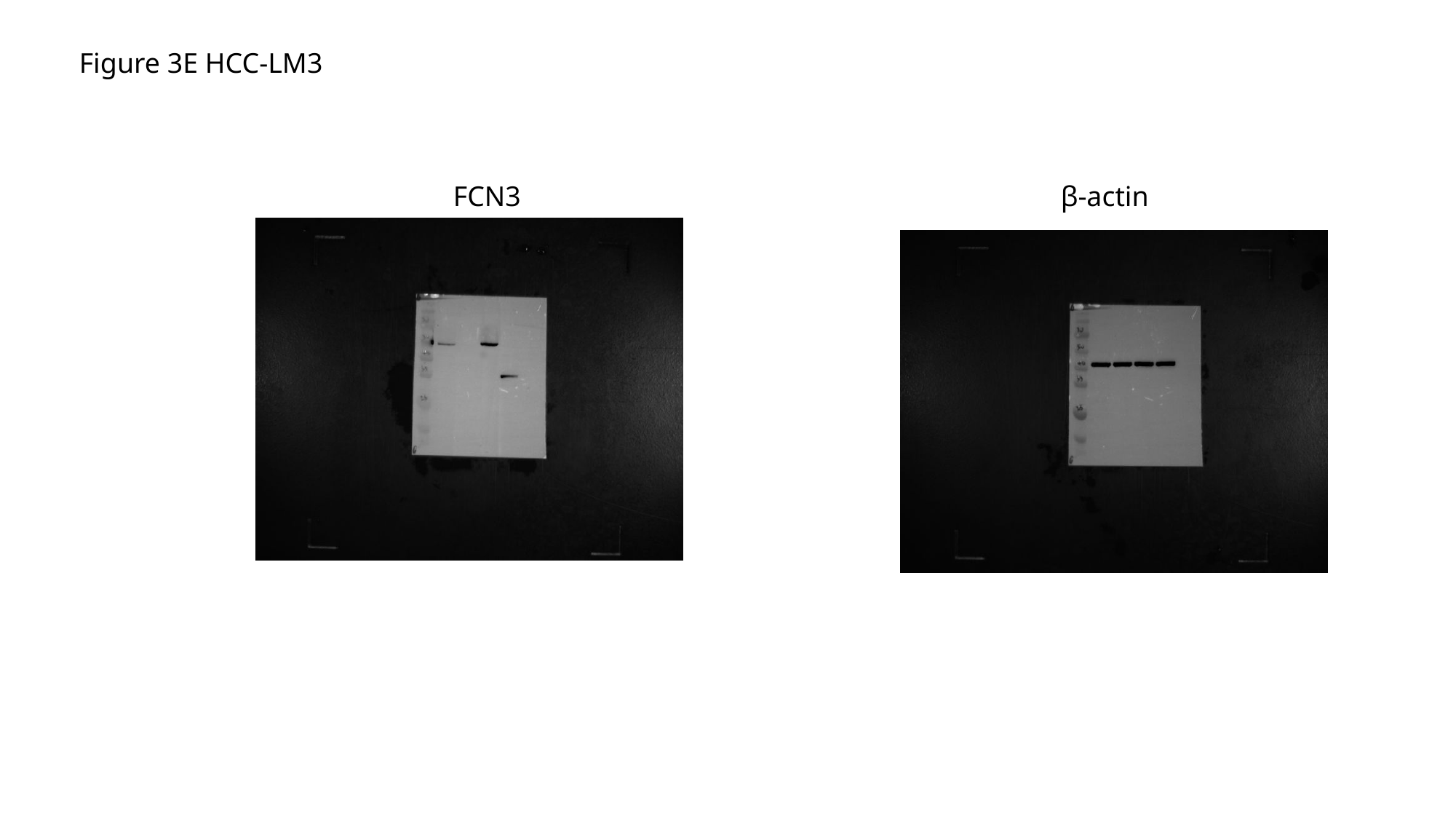

Figure 3E HCC-LM3
FCN3
β-actin

## Slide 20
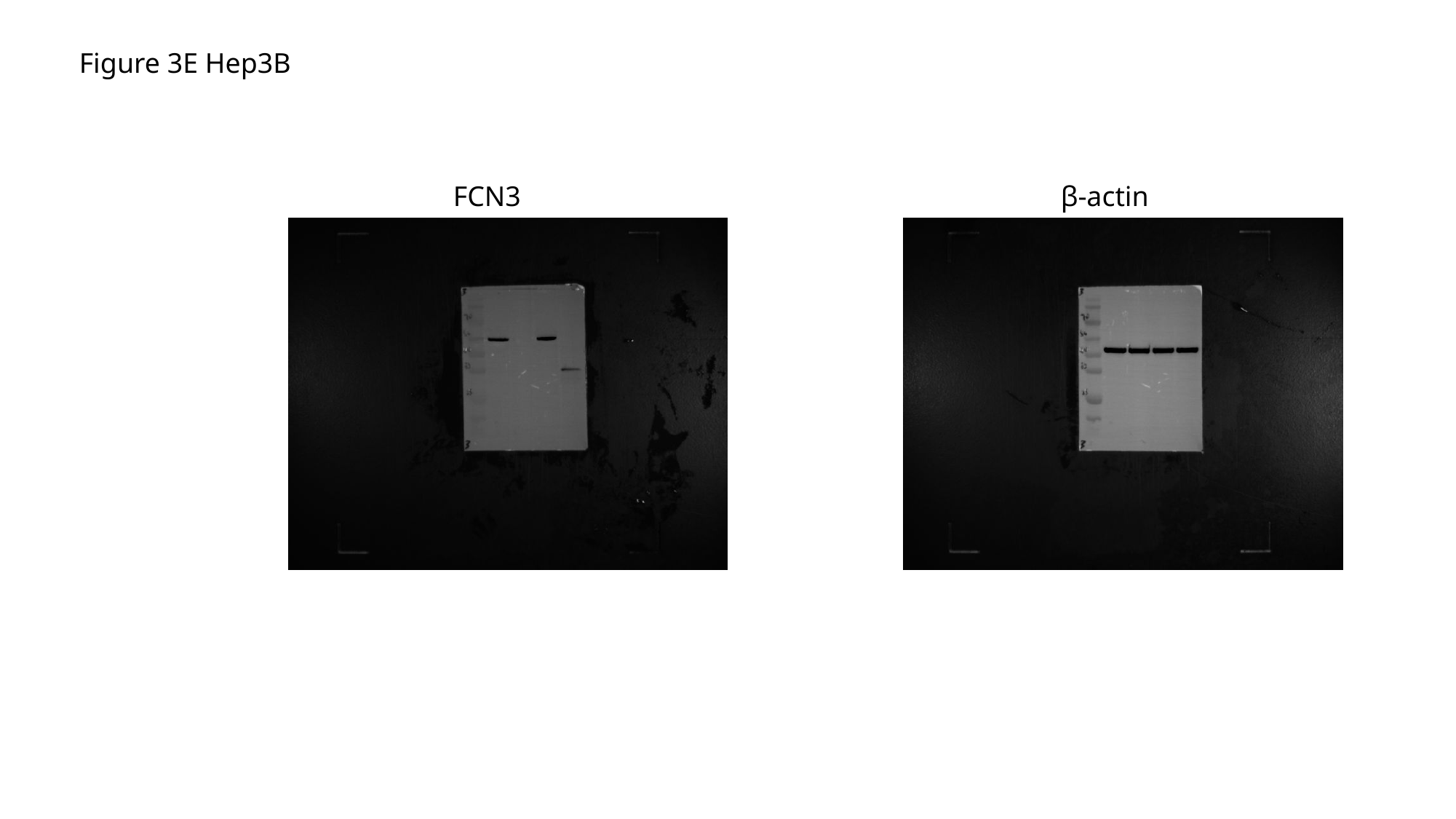

Figure 3E Hep3B
FCN3
β-actin

## Slide 21
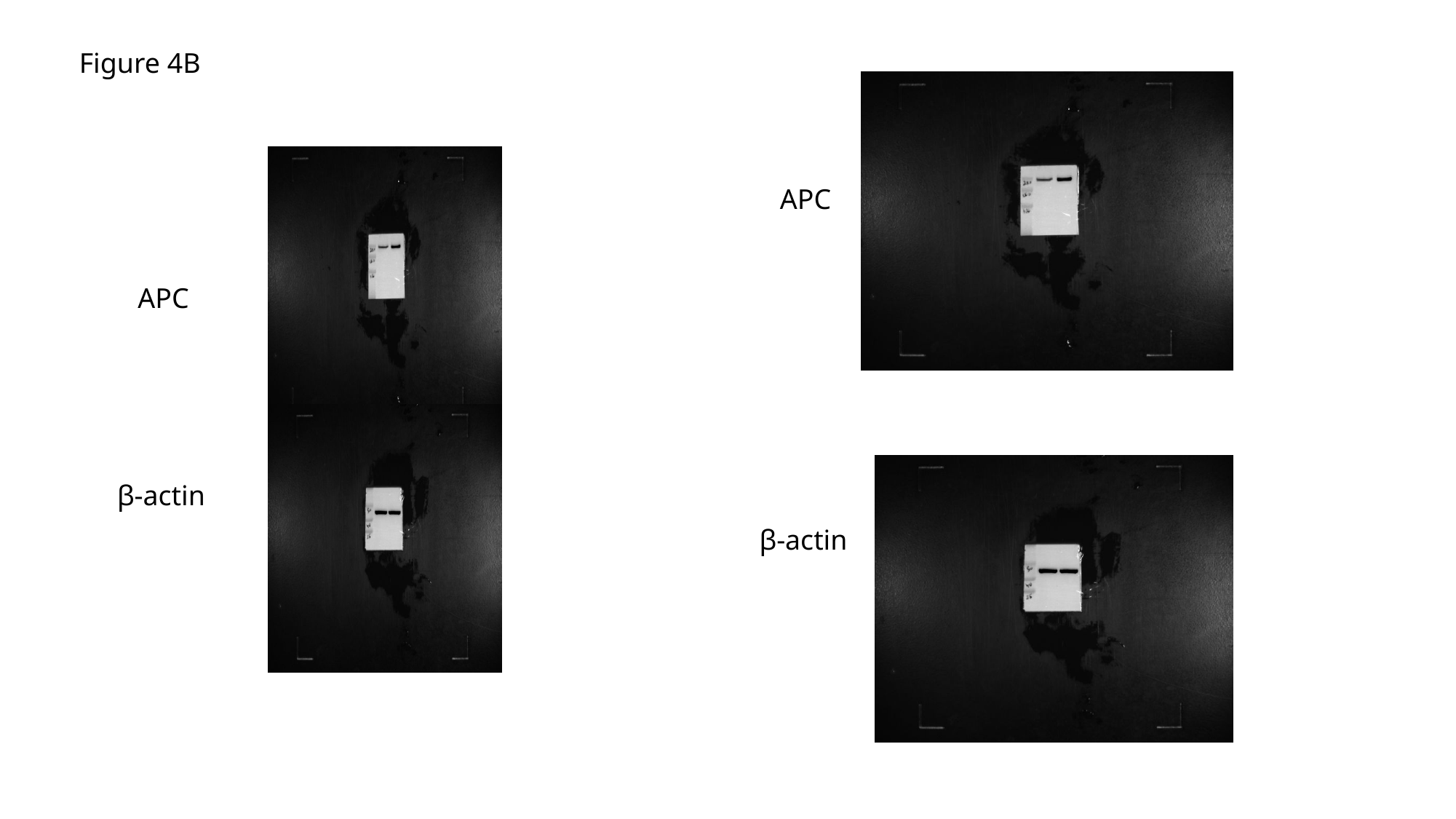

Figure 4B
APC
APC
β-actin
β-actin

## Slide 22
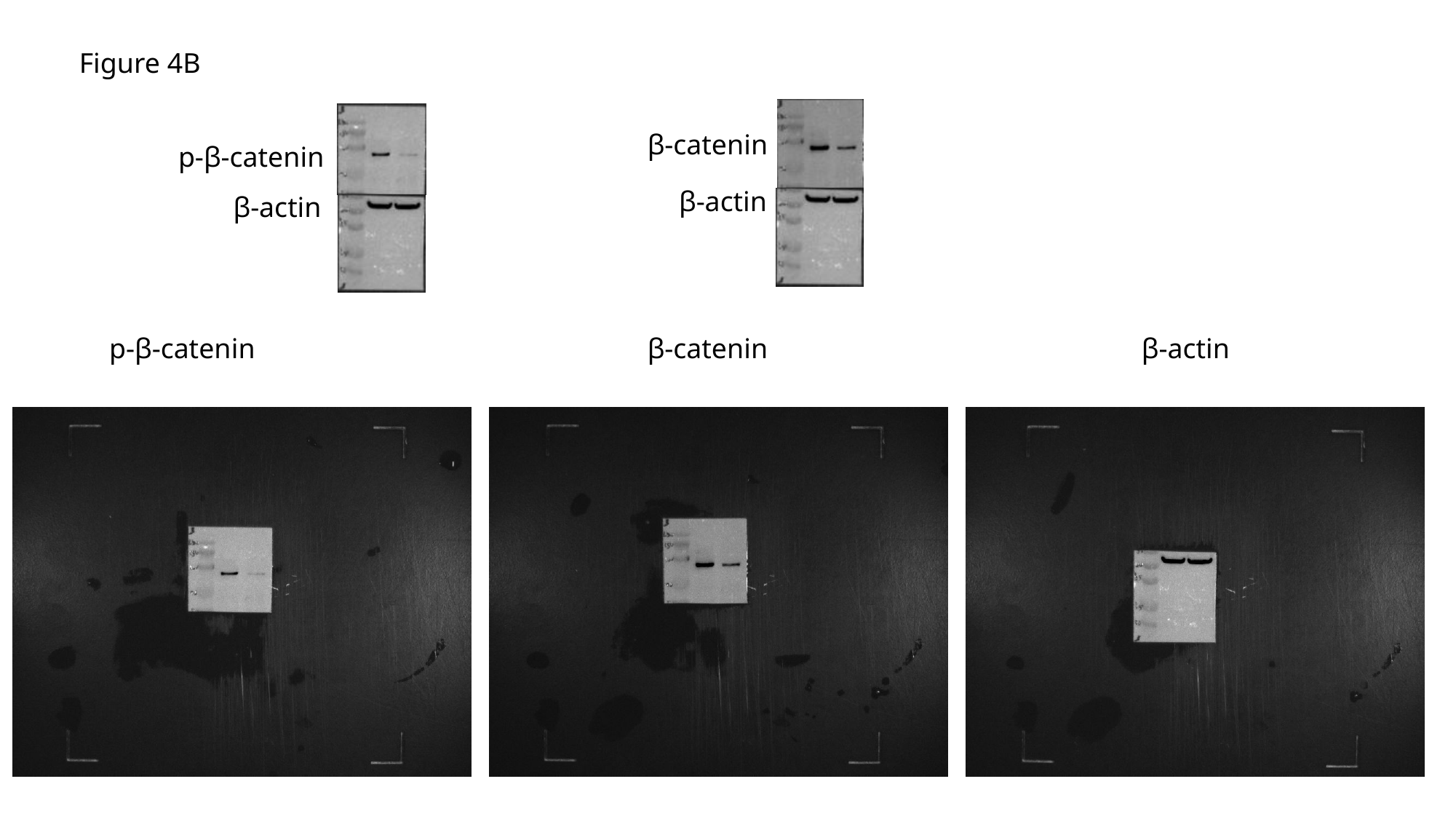

Figure 4B
β-catenin
p-β-catenin
β-actin
β-actin
p-β-catenin
β-catenin
β-actin

## Slide 23
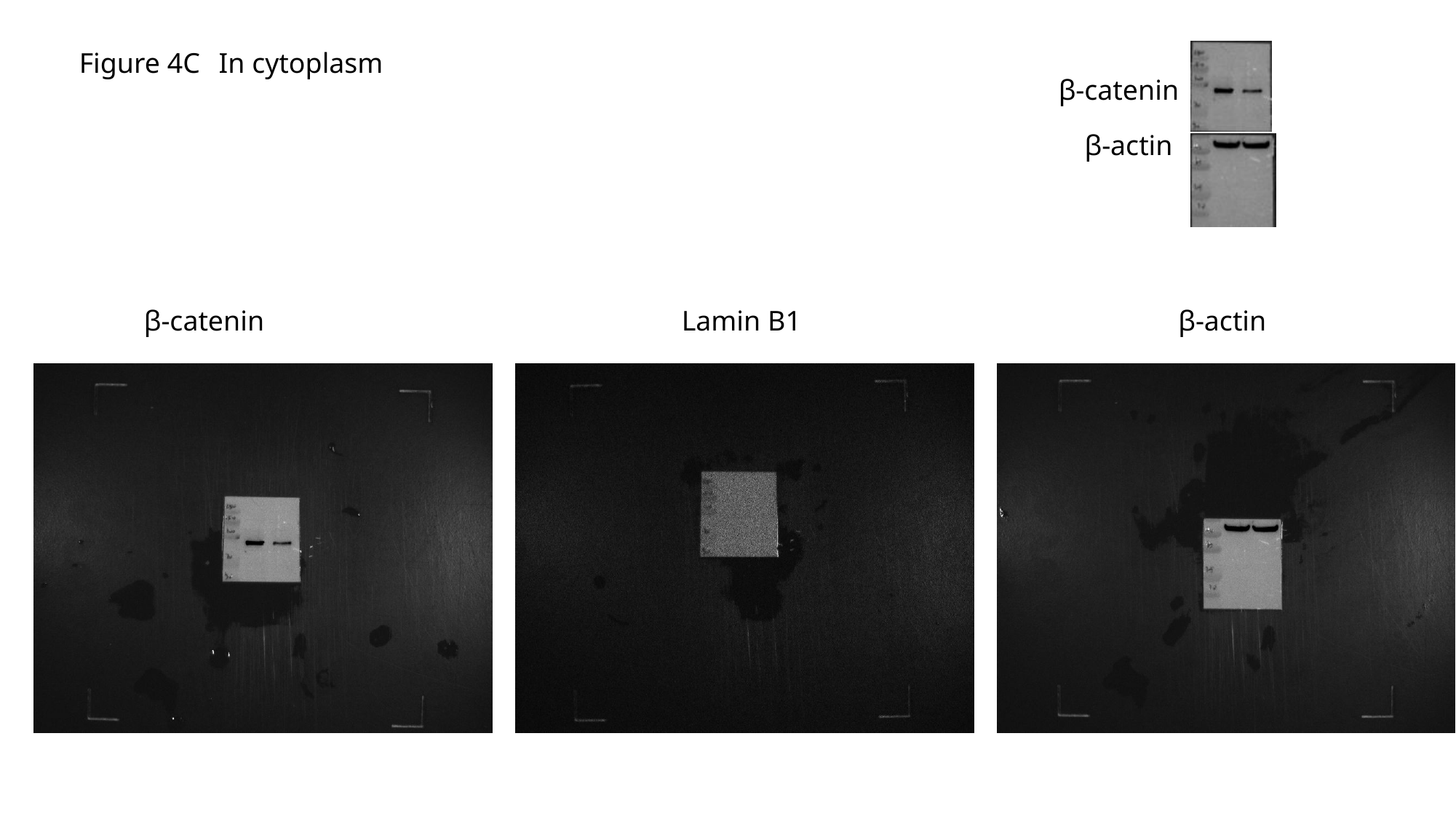

Figure 4C
In cytoplasm
β-catenin
β-actin
β-catenin
Lamin B1
β-actin

## Slide 24
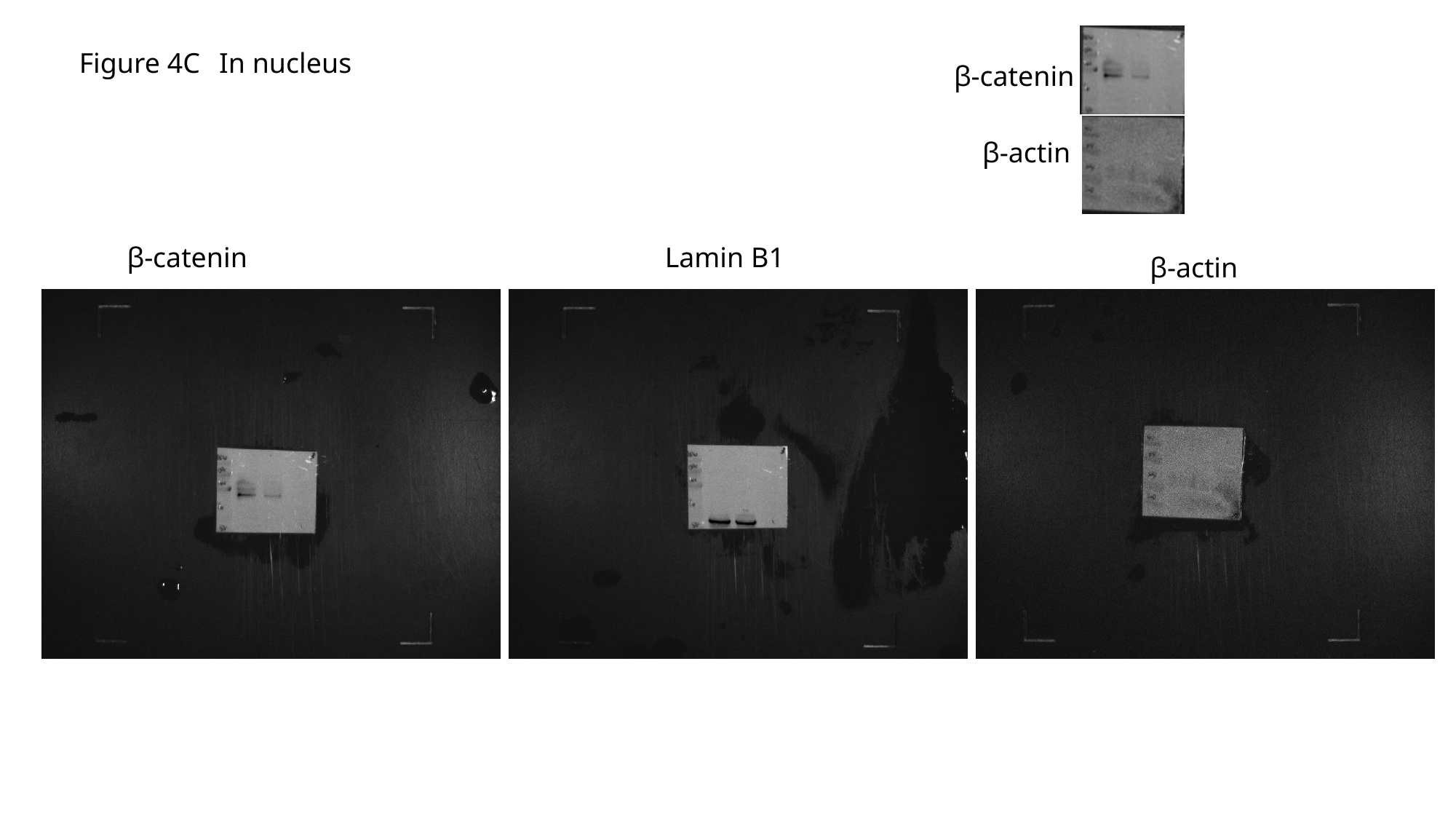

Figure 4C
In nucleus
β-catenin
β-actin
β-actin
β-catenin
Lamin B1
β-actin

## Slide 25
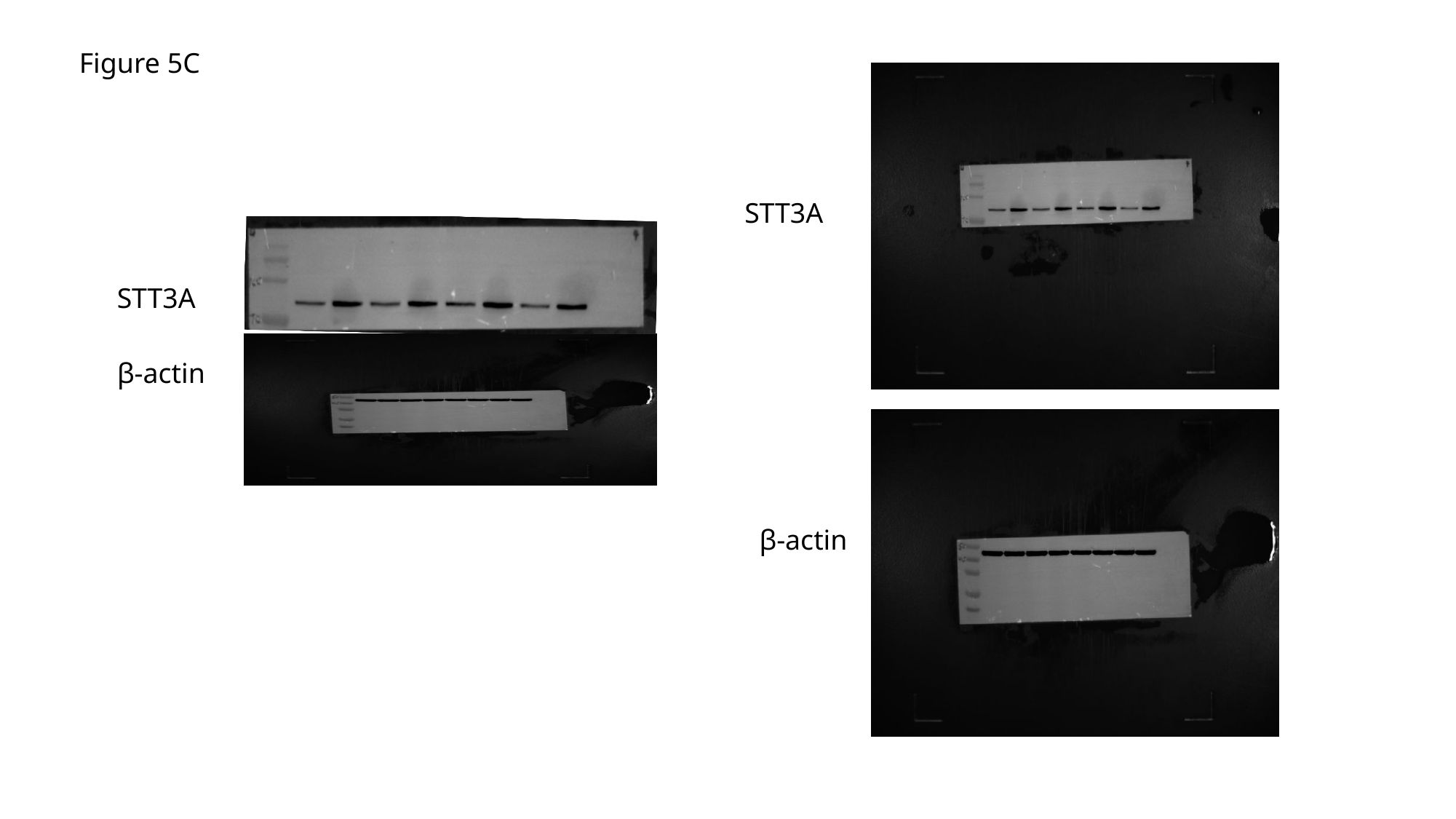

Figure 5C
STT3A
STT3A
β-actin
β-actin

## Slide 26
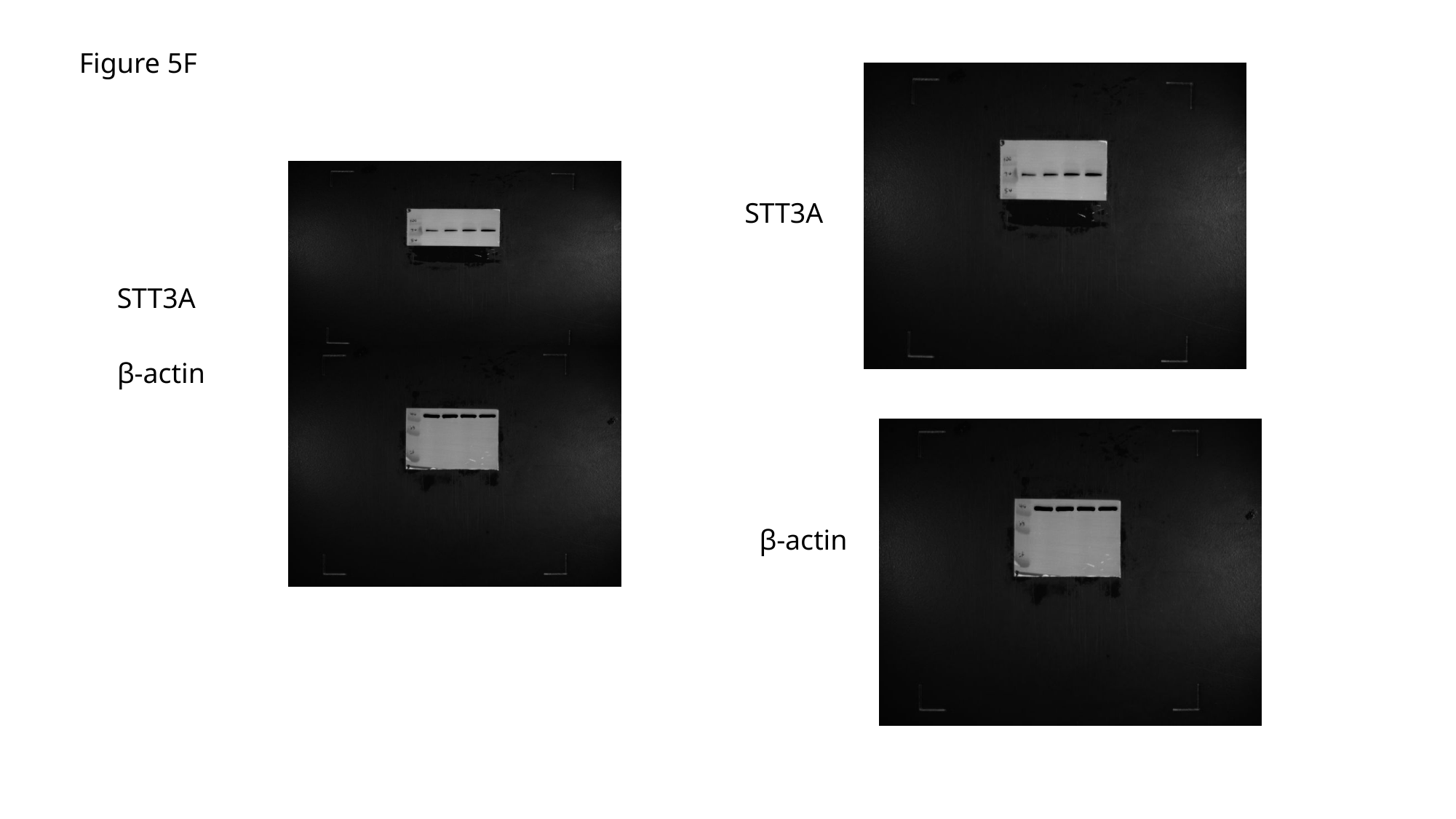

Figure 5F
STT3A
STT3A
β-actin
β-actin

## Slide 27
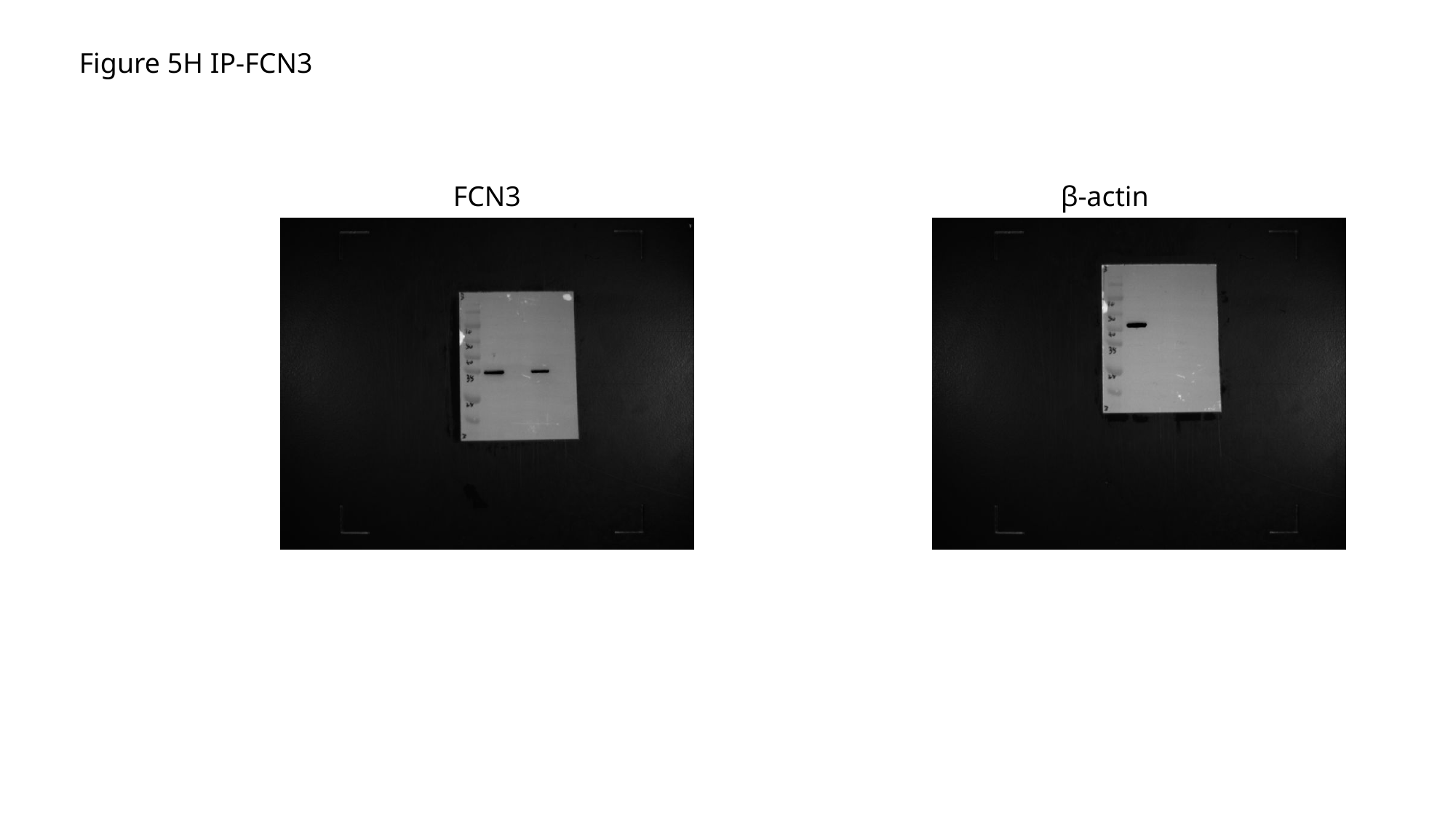

Figure 5H IP-FCN3
FCN3
β-actin

## Slide 28
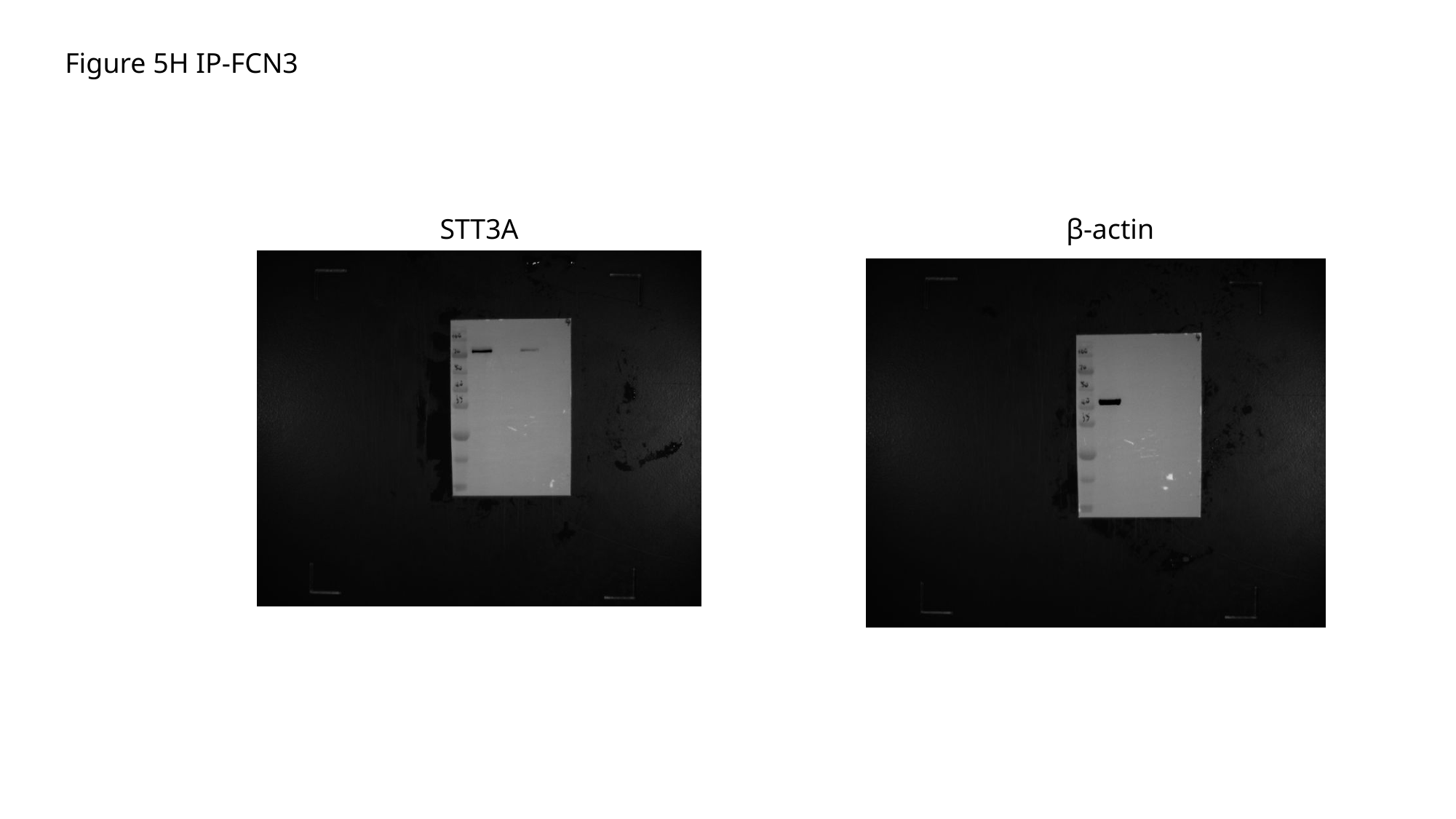

Figure 5H IP-FCN3
STT3A
β-actin

## Slide 29
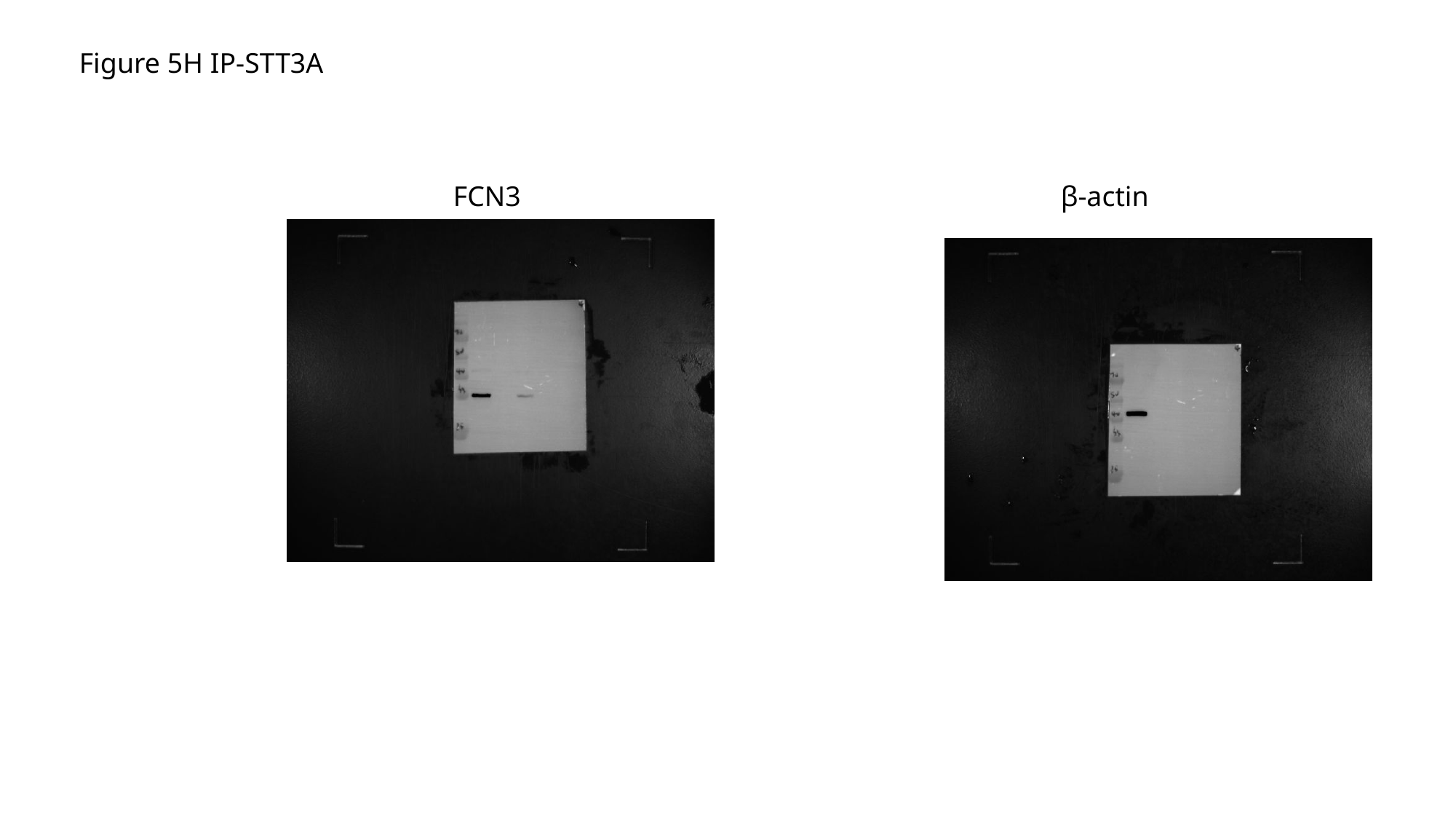

Figure 5H IP-STT3A
FCN3
β-actin

## Slide 30
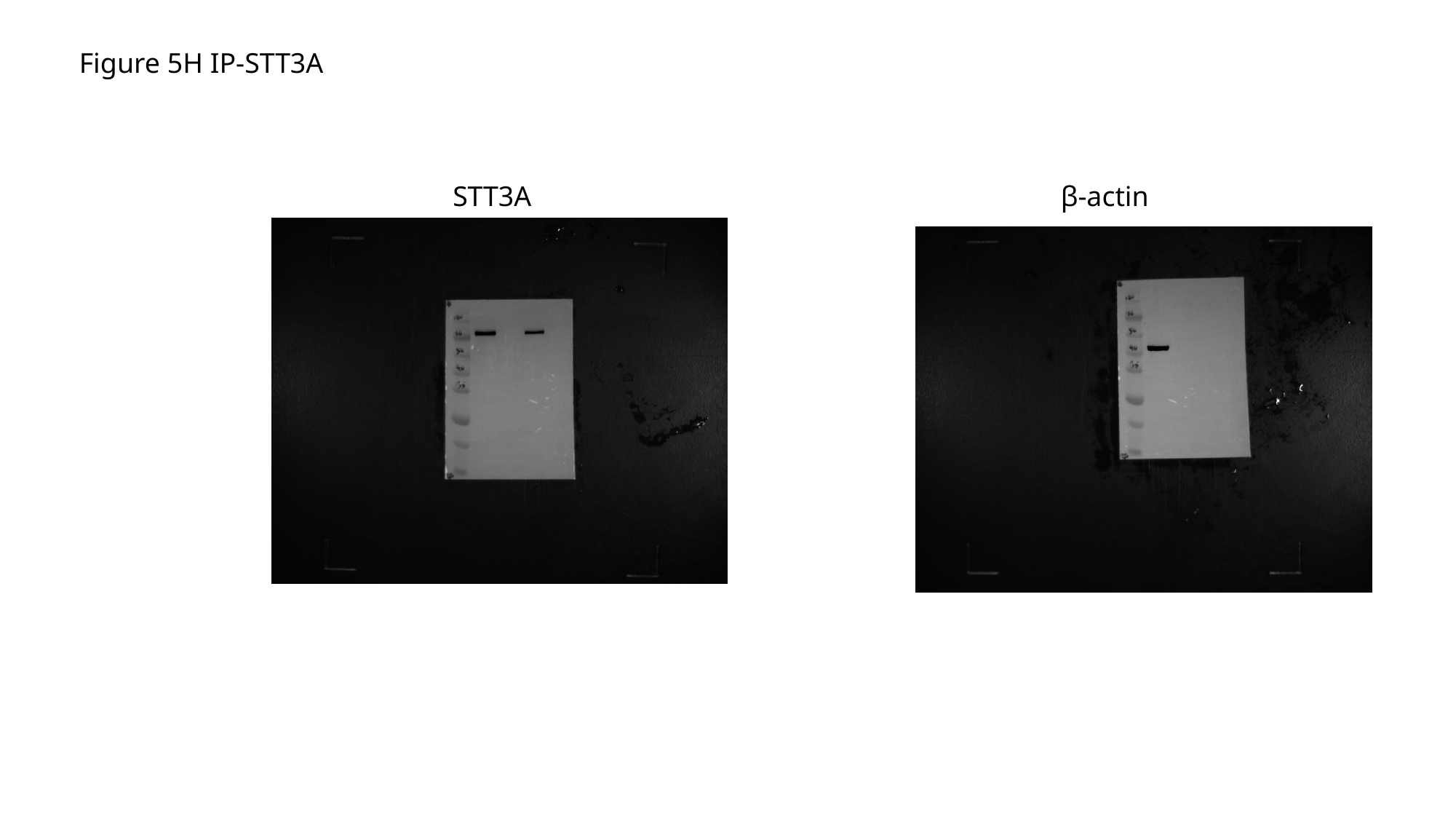

Figure 5H IP-STT3A
STT3A
β-actin

## Slide 31
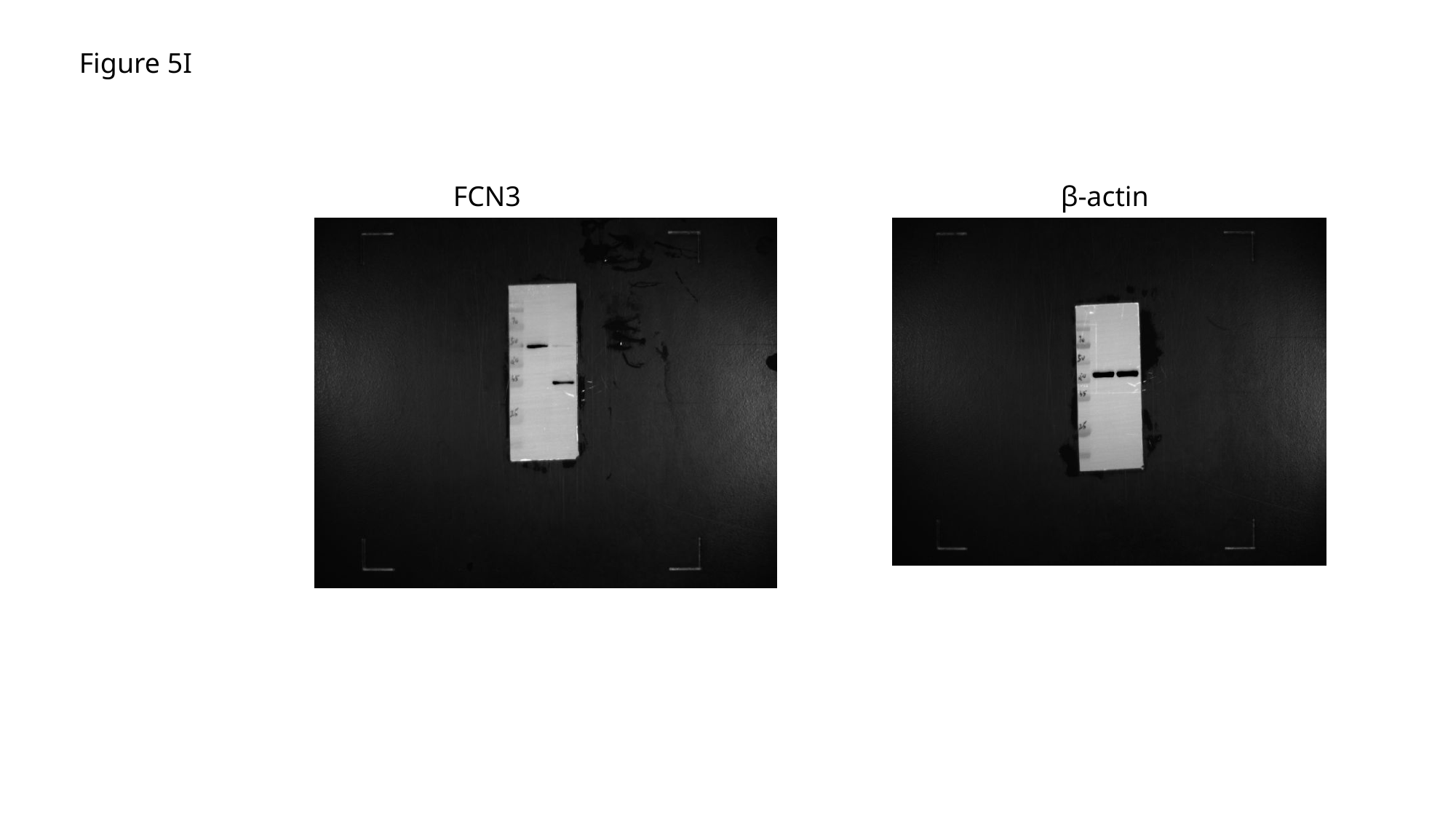

Figure 5I
FCN3
β-actin

## Slide 32
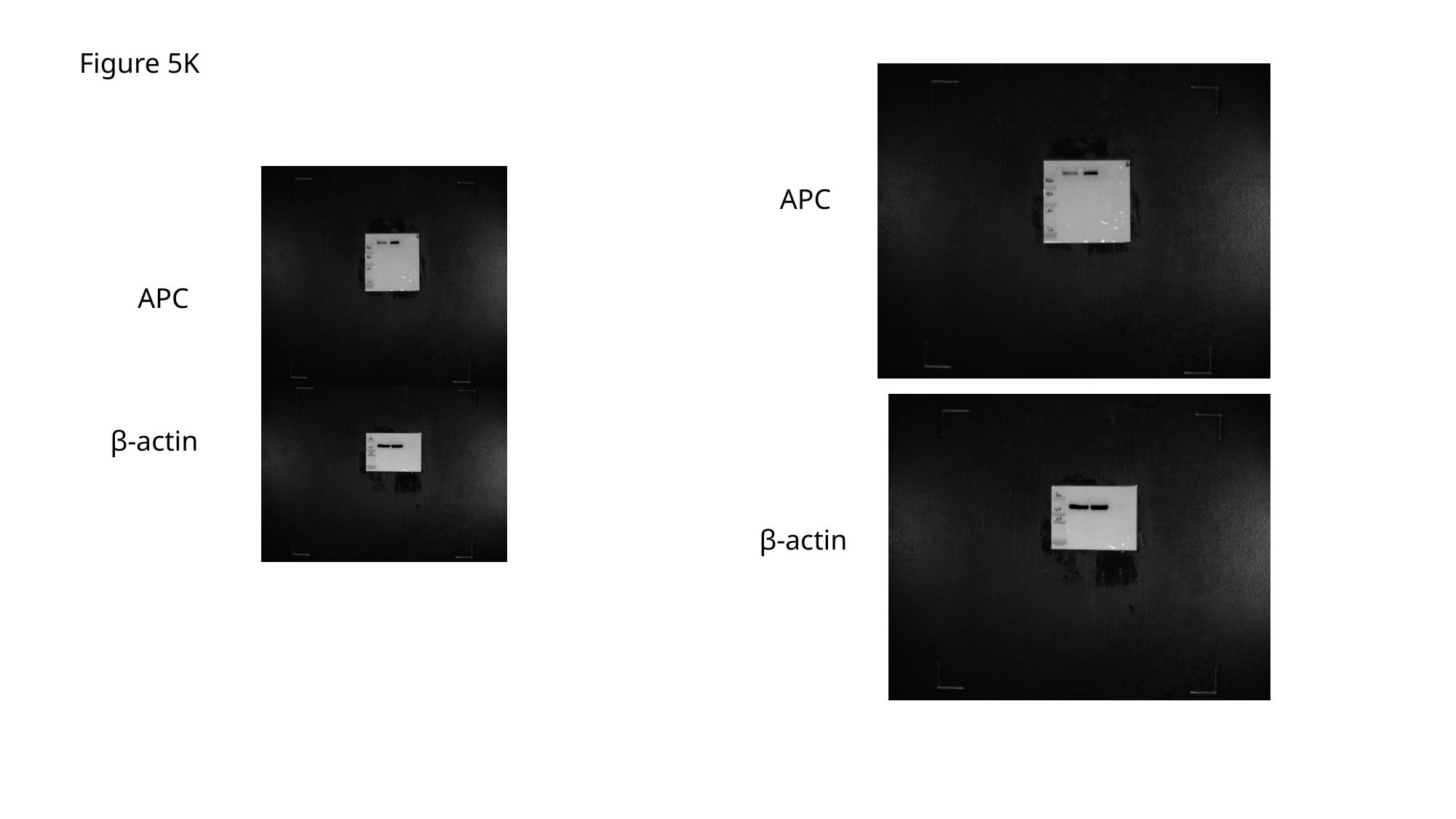

Figure 5K
APC
APC
β-actin
β-actin

## Slide 33
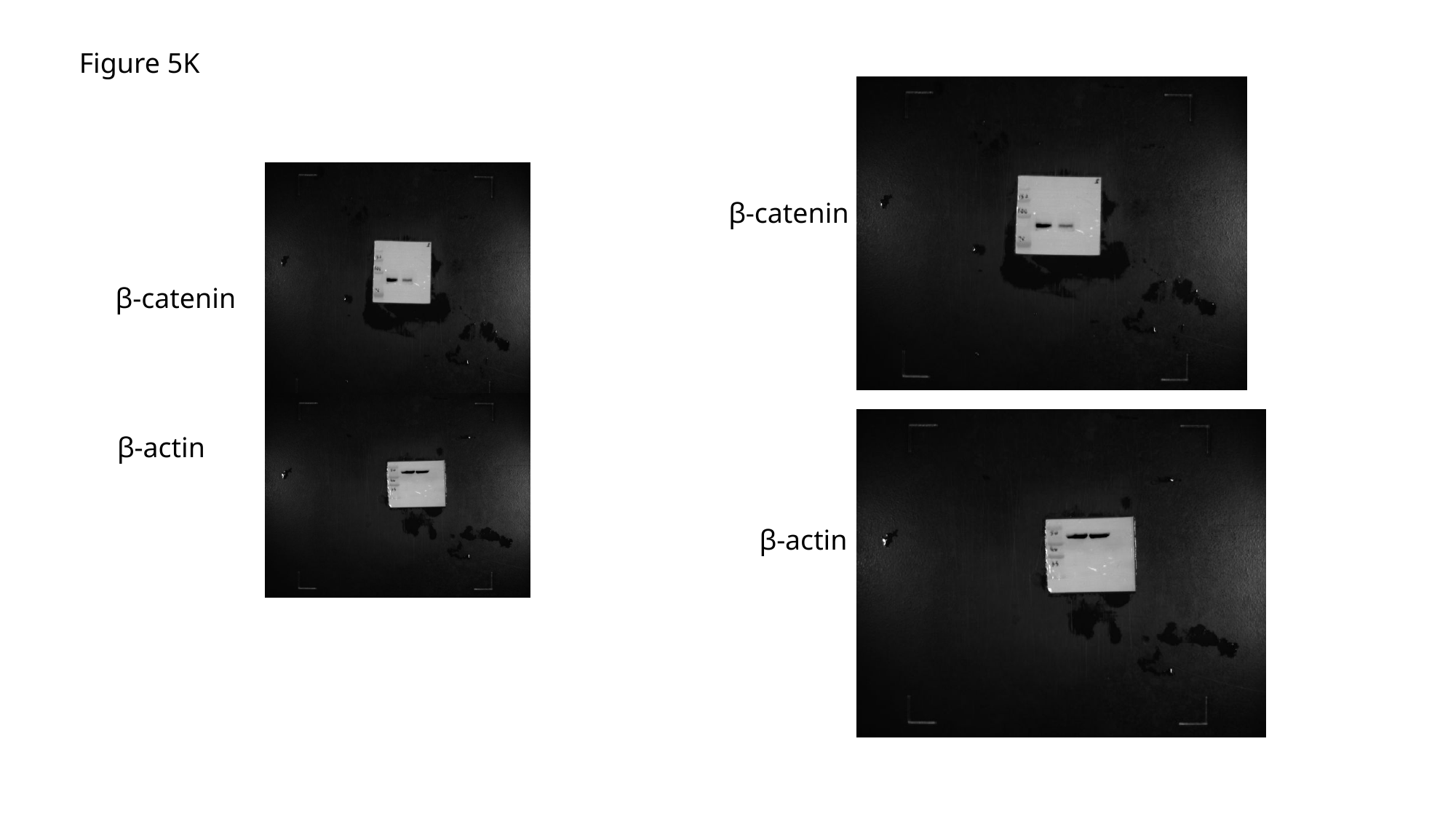

Figure 5K
β-catenin
β-catenin
β-actin
β-actin

## Slide 34
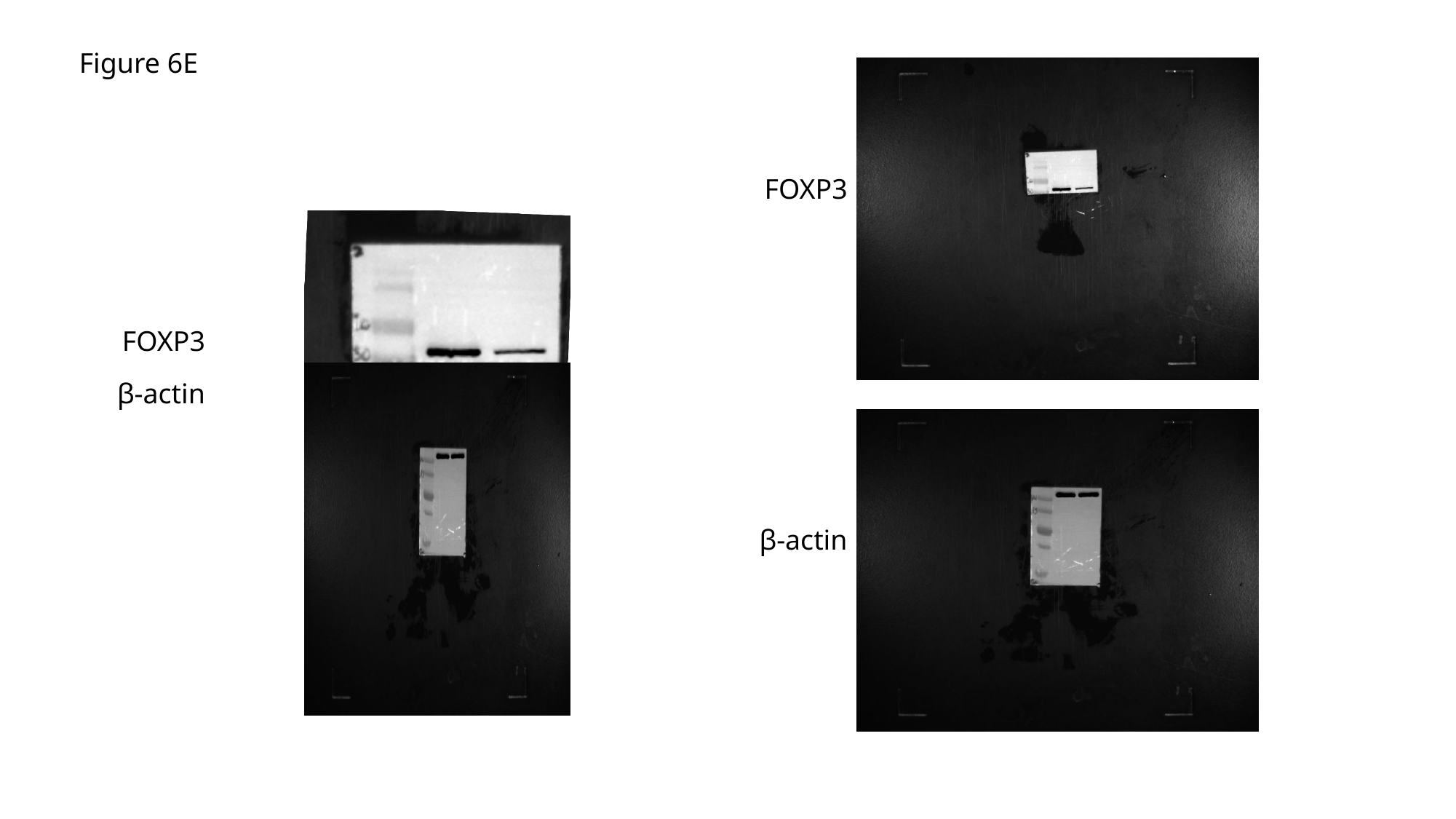

Figure 6E
FOXP3
FOXP3
β-actin
β-actin

## Slide 35
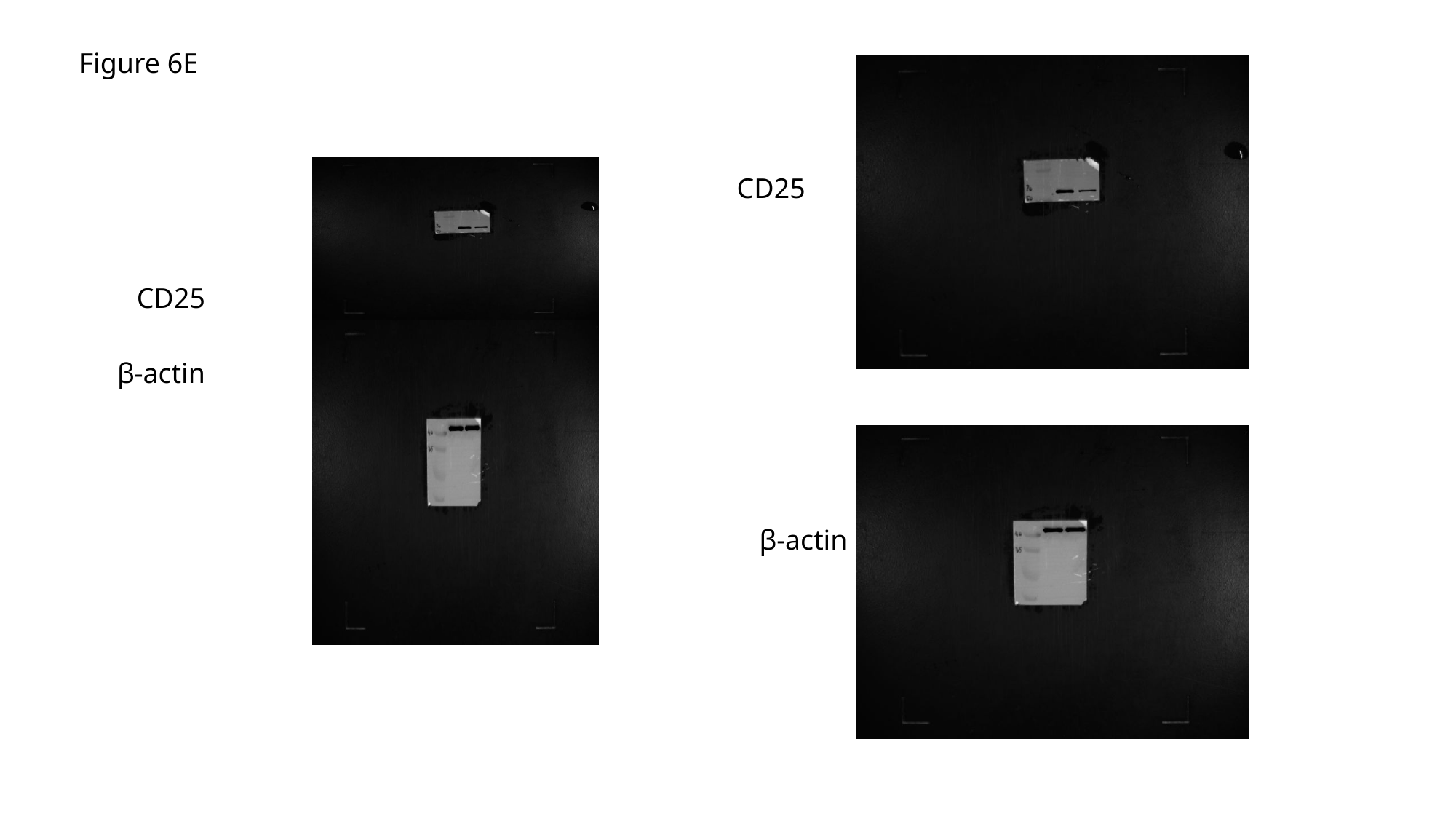

Figure 6E
CD25
CD25
β-actin
β-actin

## Slide 36
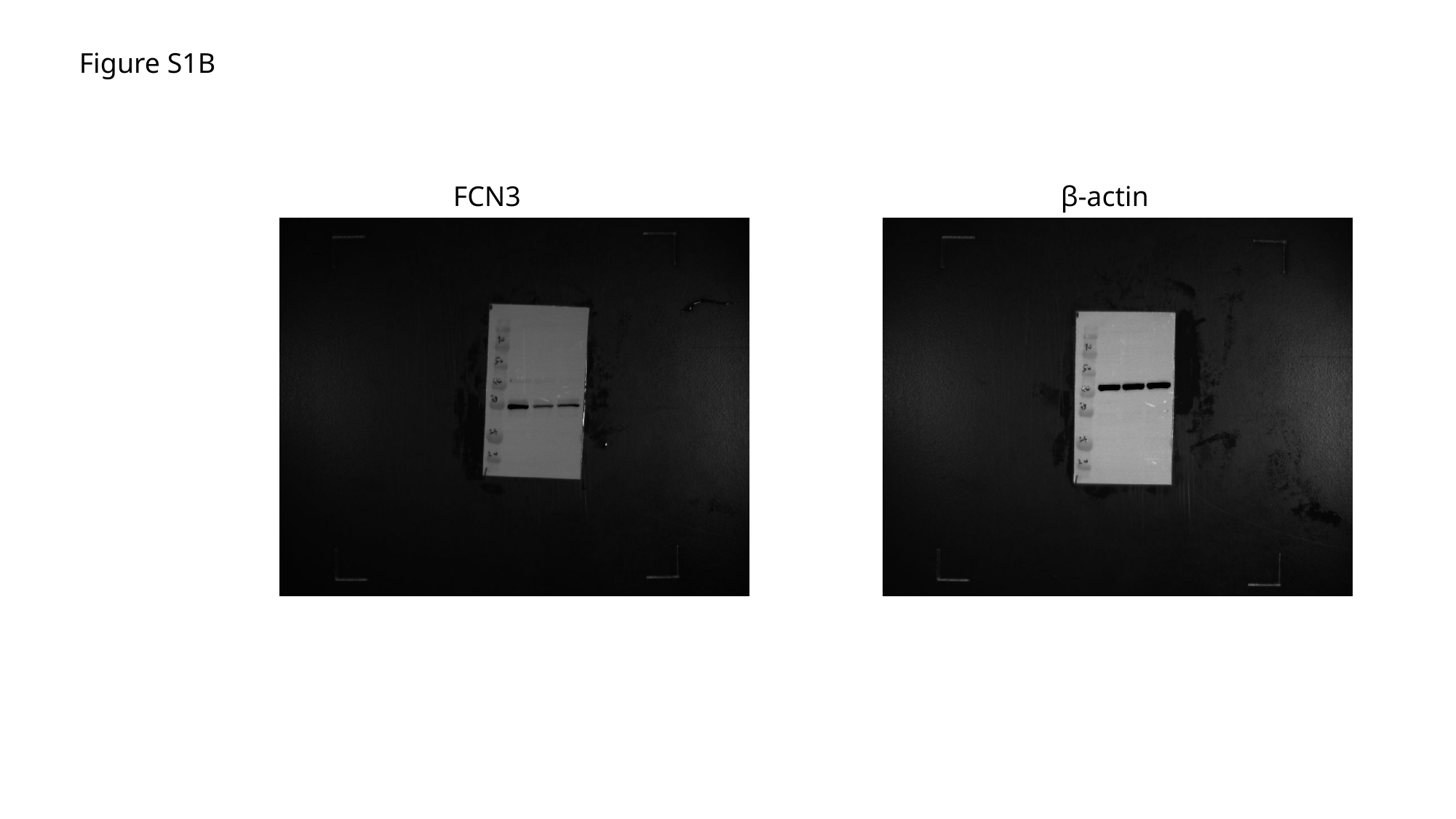

Figure S1B
FCN3
β-actin

## Slide 37
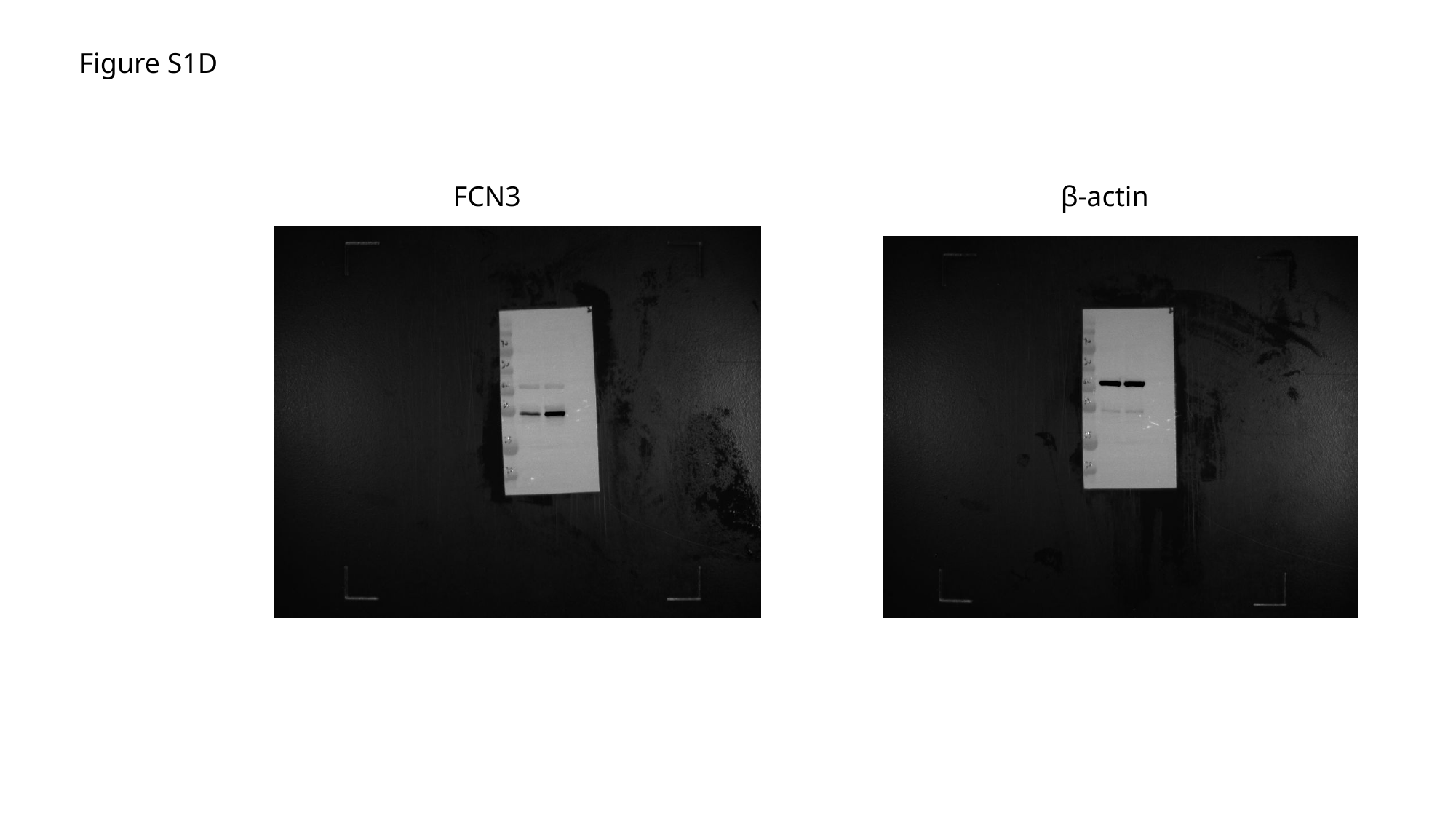

Figure S1D
FCN3
β-actin

## Slide 38
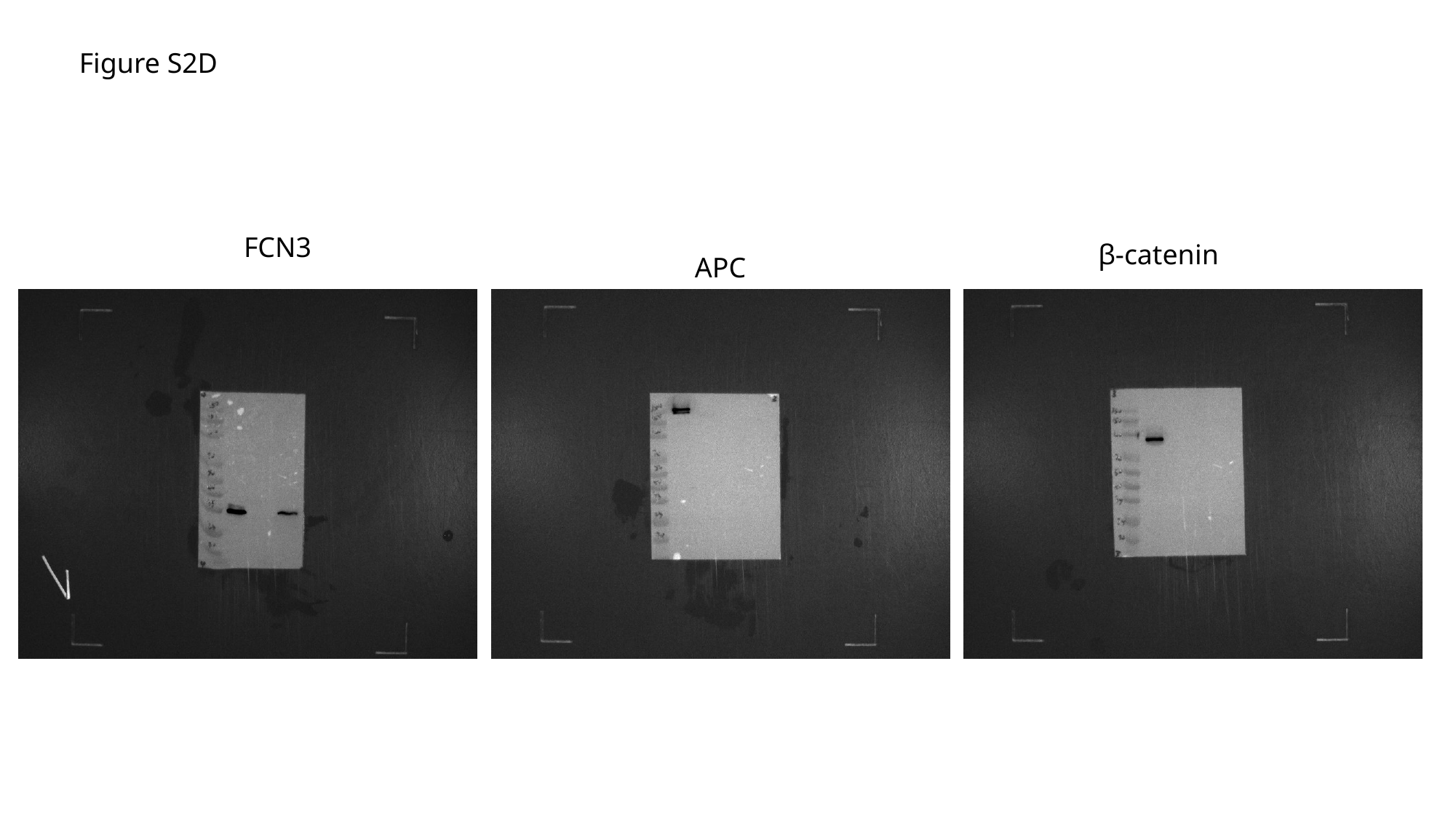

Figure S2D
FCN3
β-catenin
APC

## Slide 39
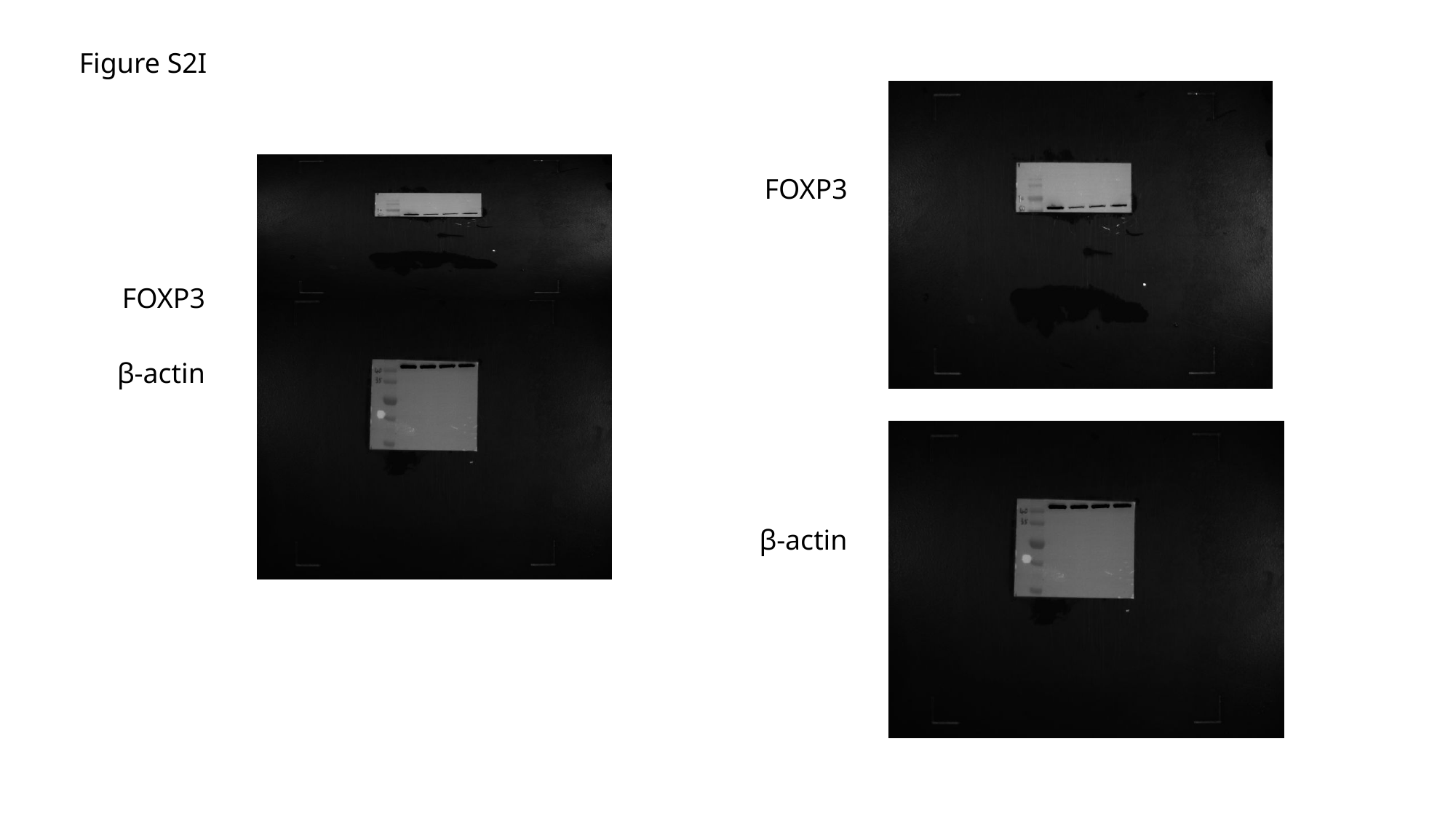

Figure S2I
FOXP3
FOXP3
β-actin
β-actin

## Slide 40
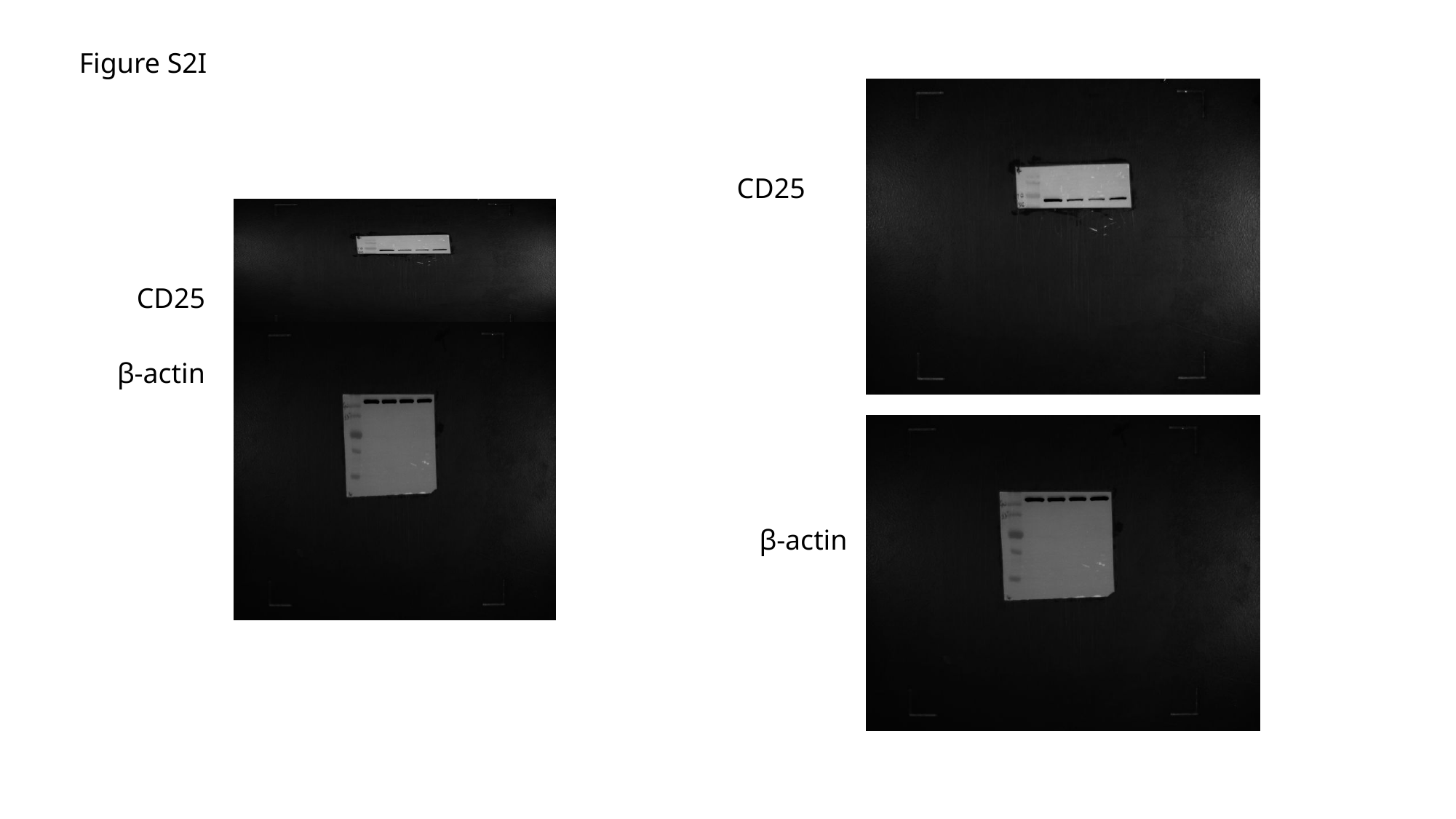

Figure S2I
CD25
CD25
β-actin
β-actin

## Slide 41
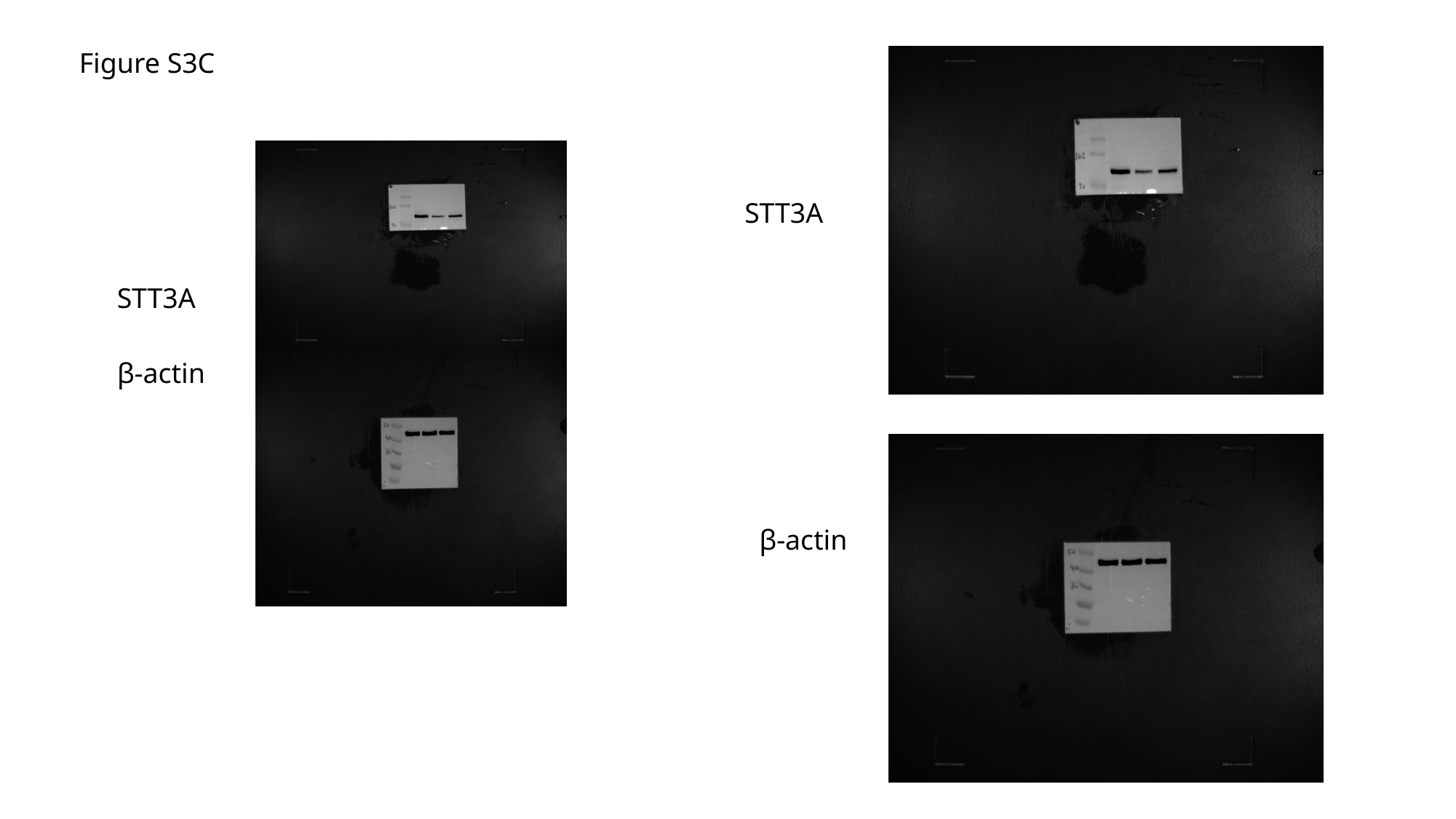

Figure S3C
STT3A
STT3A
β-actin
β-actin

## Slide 42
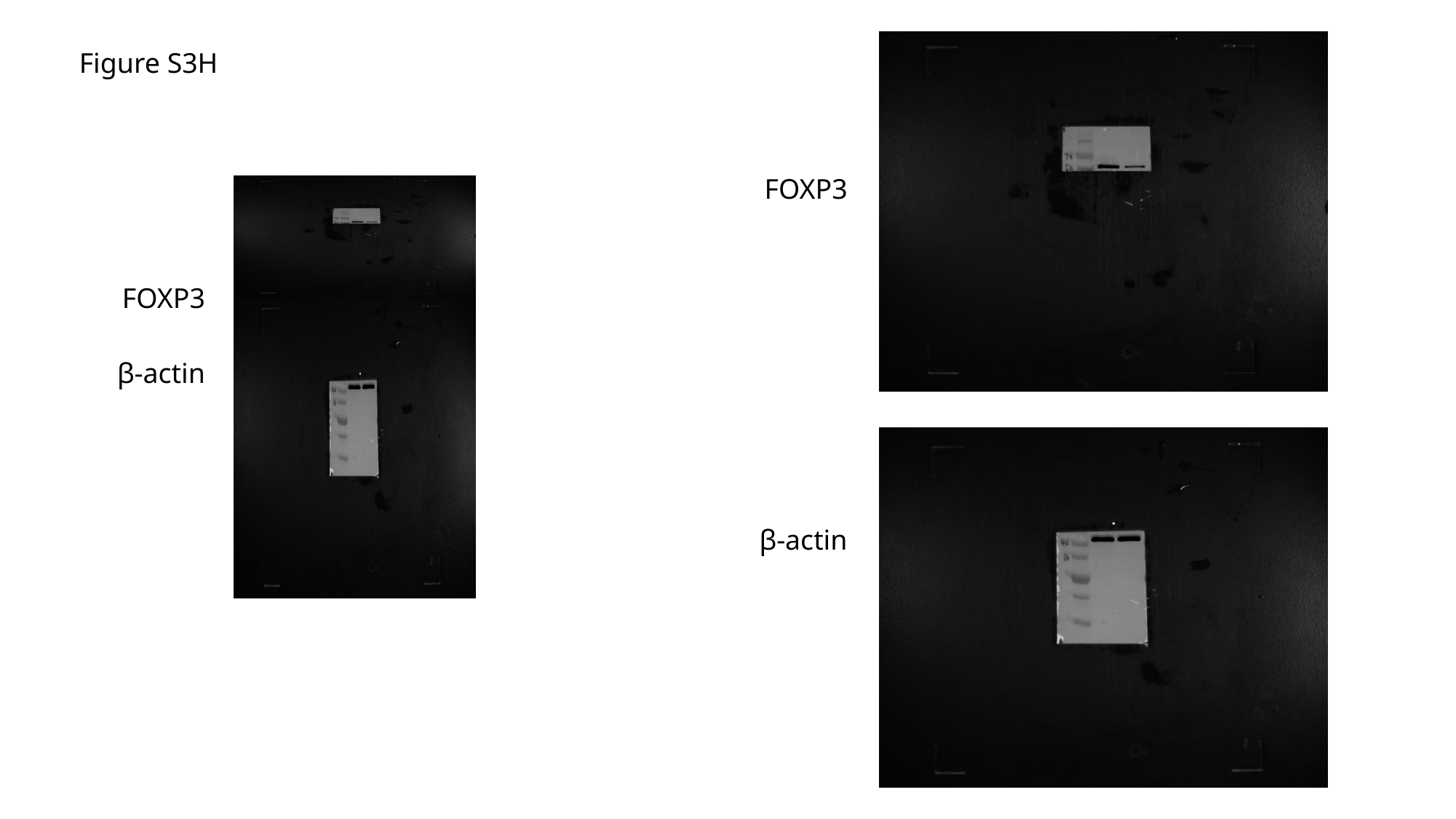

Figure S3H
FOXP3
FOXP3
β-actin
β-actin

## Slide 43
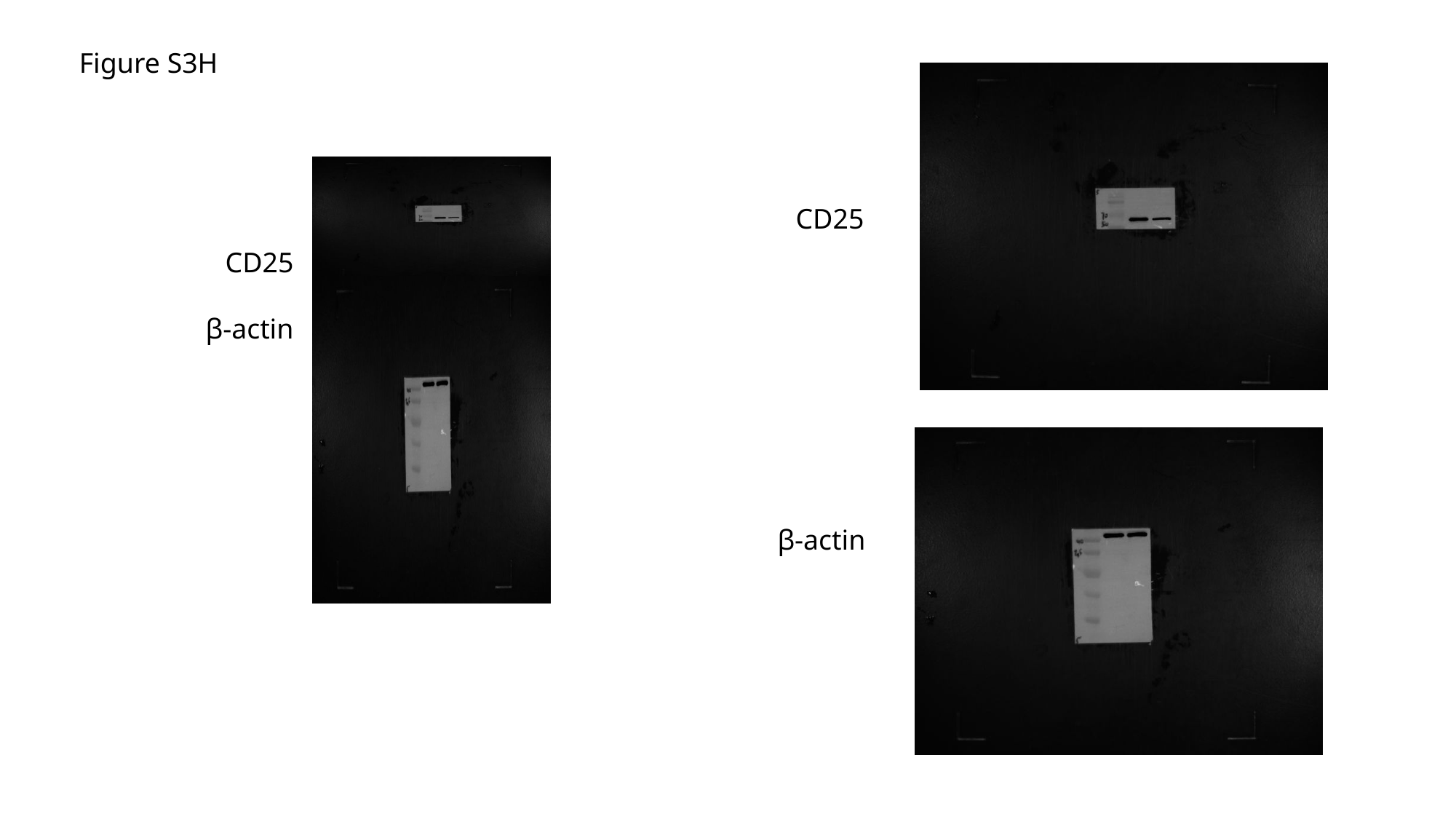

Figure S3H
CD25
CD25
β-actin
β-actin

## Slide 44
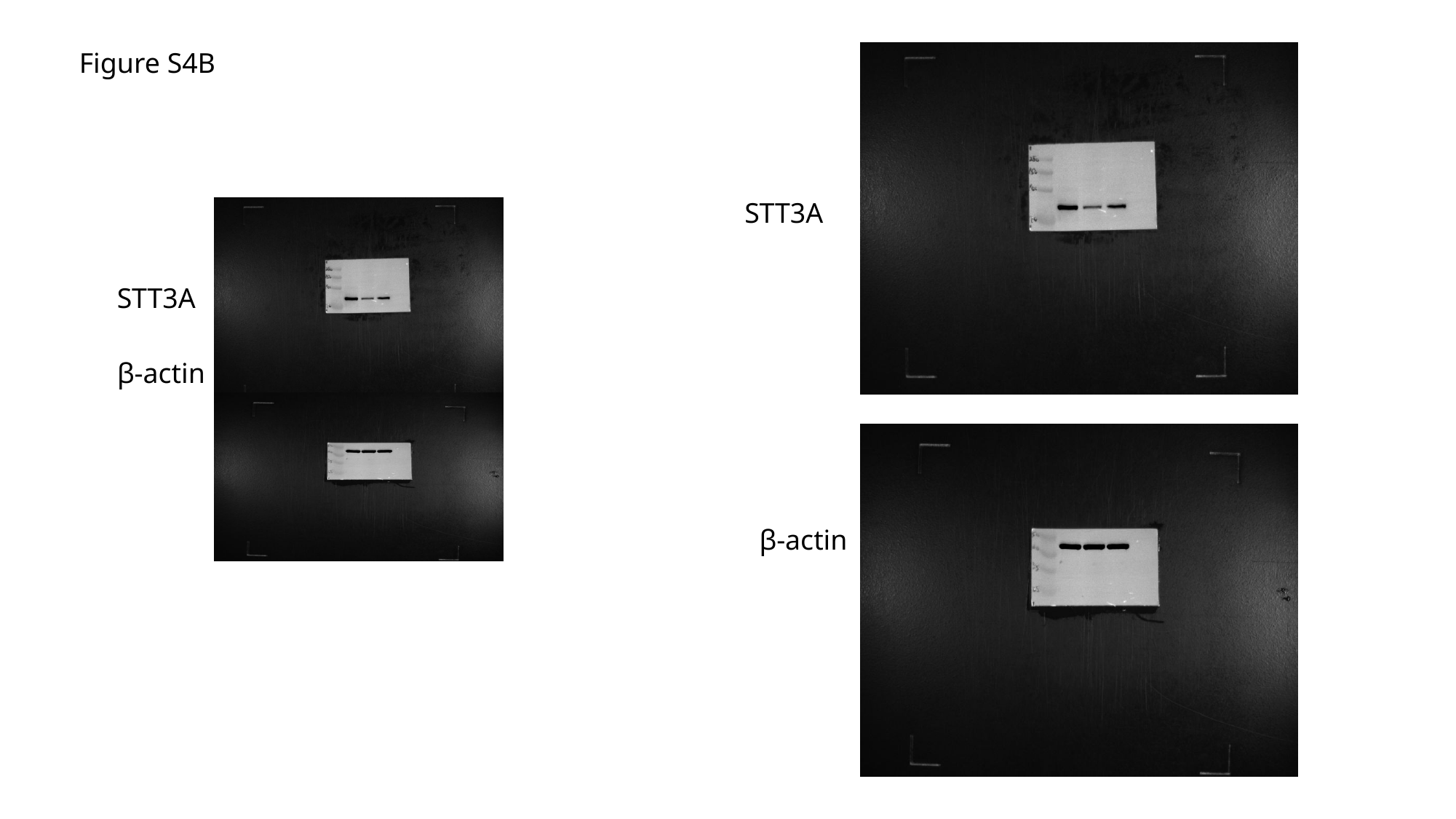

Figure S4B
STT3A
STT3A
β-actin
β-actin

## Slide 45
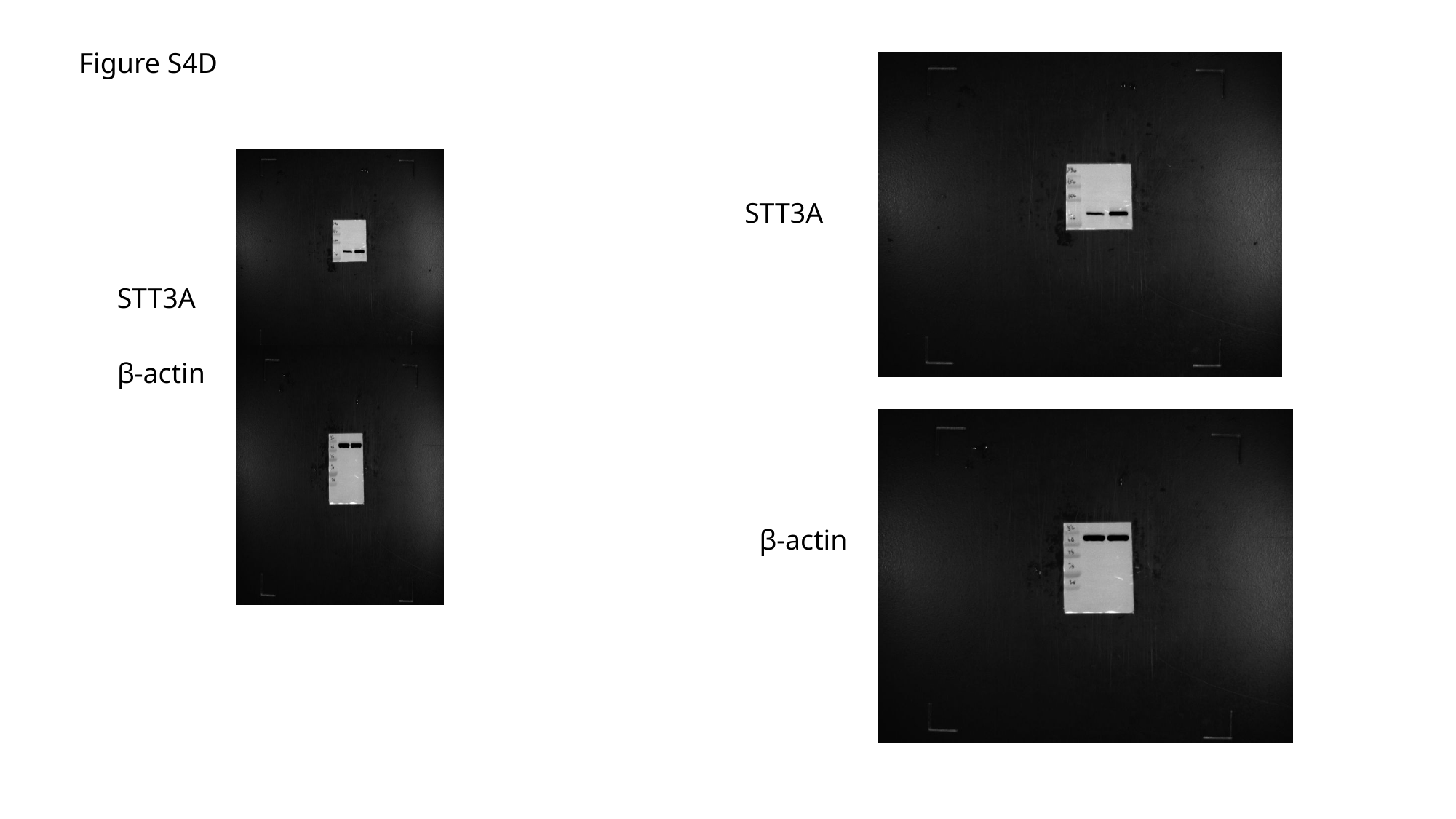

Figure S4D
STT3A
STT3A
β-actin
β-actin
